# Phyllosphere microbiomes uncovered: Research trends, geographic disparities, and key microbial players

**DOI:** 10.1590/1678-4685-GMB-2025-0083

**Published:** 2026-01-23

**Authors:** Isabel Cristina Cadavid, Érika Frydrych Capelari, Caroline Salvati, Rogerio Margis

**Affiliations:** 1 Universidade Federal do Rio Grande do Sul, Centro de Biotecnologia, Departamento de Biofísica, Porto Alegre, RS, Brazil.; 2 Universidade Federal do Rio Grande do Sul, Instituto de Biociências, Departamento de Genética, Porto Alegre, RS, Brazil.

**Keywords:** Phyllosphere, microbiome, Next-Generation Sequencing, Systematic review

## Abstract

In recent years, significant efforts have been made to understand the phyllosphere microbiome. To clarify current research trends and identify knowledge gaps, we conducted a systematic review of the literature and investigated the methodologies used to analyze microbiome communities associated with plants, along with the objectives of these studies. Applying systematic review principles, we assessed 333 reports from the Web of Science database for eligibility. Articles were included if they presented original research on microbiomes associated with phyllosphere tissues identified by next-generation sequencing. Of these, 268 reports, published from 2009 to March 2025, were retrieved. These reports were used to extract data in a controlled and methodical manner. The analyses identified the most frequently studied plant species and primary tissues, the geographical locations sampled, the variables investigated in the phyllosphere microbiome, and the methodologies and tools employed. A comparison of the number of studies on below-ground (n=1562) versus above-ground tissue (n=375) underscores the relatively unexplored nature of above-ground research. We notice a surprisingly low number of studies from the Southern Hemisphere. Additionally, through data mining, we identify the most dominant bacteria and fungi reported in phyllosphere studies. Based on these findings, we offer recommendations for future research on the phyllosphere.

## Introduction

Microorganisms can establish associations with plant tissues and have a beneficial effect on plant health and productivity. They are distributed in all plant tissues: below-ground (rhizosphere and root) and above-ground (phyllosphere) (Santoyo et al., 2017). The plant microbiome can promote nutrient solubilization, fix nitrogen, produce phytohormones, limit pathogen growth, enhance plant defense systems, control insect pests, and increase resilience to stress (Compant et al., 2005; Ryan et al., 2008; Santoyo *et al*., 2012). 

The phyllosphere encompasses the aerial parts of plants, including leaves, flowers, stems, fruits, and pollens (as reviewed by Sohrabi et al., 2023). Microorganisms residing on the surface of the phyllosphere are known as epiphytes, while those inside phyllosphere tissues, within intercellular spaces (apoplast) or plant cells, are called endophytes. The microbiota of the phyllosphere plays a crucial role in global carbon and nitrogen cycles, promotes plant growth, enhances stress tolerance, and aids in biocontrol against pathogens (Sohrabi *et al*., 2023).

Research in phyllosphere microbiology has a rich history, evolving significantly through different eras. Initially, before the metagenomics era, pioneering researchers focused on culturable bacteria and fungi inhabiting the phyllosphere (Yang et al., 2001; Perez et al., 2009; Yashiro et al., 2011). The advent of omic tools marked a major shift, enabling access to information on non-culturable microbial communities. The introduction of high-throughput sequencing technologies such as 454 pyrosequencing, ion torrent, and Illumina has revolutionized microbial ecology research in the phyllosphere. These technologies facilitate diverse approaches including sequencing of 16S ribosomal RNA (rRNA) genes, ribosomal DNA internal transcribed spacer (ITS) regions, shotgun metagenomics and metatranscriptomics, and whole-genome sequencing, thereby transforming our understanding of phyllosphere microbial communities (Vokou et al., 2019; Cai et al., 2024; Masuda et al., 2024).

Over the years, significant efforts have been dedicated to developing bioinformatic tools for analyzing microbiomes through DNA sequencing. These tools encompass a range of functionalities, including processing sequence libraries, performing quality analysis, clustering sequences, identifying taxa, assessing community functions and correlations, and analyzing community diversities. These advancements have greatly enhanced our ability to taxonomically identify and understand microbial communities, driving forward research in microbiome science (Schloss et al., 2009; Kuczynski et al., 2011).

**Figure 1 -:**
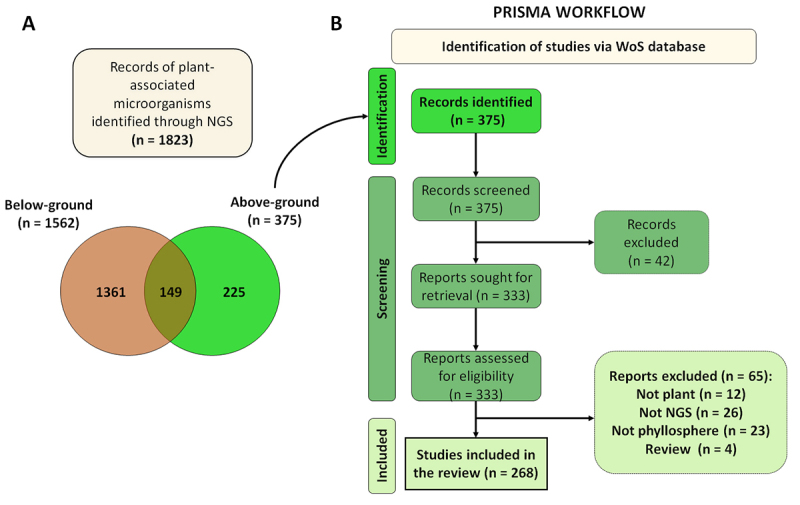
Overview of plant microbiome studies. a. Number of records related to plant microbiome studies using Next Generation Sequencing (NGS), highlighting below-ground tissues (root, rhizosphere) in brown and above-ground (phyllosphere or leaf) in green **b.** PRISMA workflow illustrating the identification, screening and inclusion of articles specifically related to the plant phyllosphere.

The objective of this study was to systematically review methodologies commonly used for microbiome data preparation and analysis in plant-bacterial and fungal interactions. We comprehensively compiled data on phyllosphere analyses using Next-Generation Sequencing (NGS) (Figure 1) and mapped the plant species, tissues, compartments (endophytic or epiphytic), geographical locations, and conditions (biotic and abiotic) that have been studied to date. By identifying knowledge gaps and understudied areas, this review provides a resource for novice researchers seeking foundational information on plant microbiomes and a guide for experts aiming to explore additional factors influencing plant-microbe relationships.

## Material and Methods

### Microbiome phyllosphere-literature search strategy and eligibility criteria

We examined the research landscape of plant microbiomes using the Web of Science (WOS) database. We searched for records that investigated plant-bacteria and fungal relationships using Next-Generation Sequencing (NGS) (refer to string search in Table S1, item a). Specifically, phyllosphere microbiome-related articles were searched using terms combined with Boolean operators to search by “title, abstract, keyword plus, and author keywords.” The search string is described in Table S1 (Item b) and referred to as above-ground tissues. The intention was to retrieve studies about phyllosphere bacterial and fungal microbes identified through next generation sequencing**;** we limited the search timeframe from January, 2009 up to March, 2025. Some articles were excluded based on specific criteria: if they were reviews, did not involve Next-Generation Sequencing (NGS), were not focused on the phyllosphere, or did not involve plants. The work was conducted in accordance with the Preferred Reporting Items for Systematic Reviews and Meta-Analyses statement (PRISMA) (Page et al., 2021). 

### Comparative analysis between above-ground and below-ground tissues

To provide an overview of the number of studies on above-ground versus below-ground plant tissues, we conducted searches in the Web of Science (WoS) database. Studies focusing on below-ground tissues, including the rhizosphere or roots and involved NGS and potentially encompassing the phyllosphere, as well, were identified (see string in Table S1, item c). Additional searches were conducted to determine the number of studies exclusively focused on each plant region: above-ground (Table S1, item d), and below-ground tissues (Table S1, item e). These data were graphically represented using a Venn diagram (Figure 1 a ).

### The dominant phyllosphere microbiome

We conducted a data mining analysis to identify the most dominant bacterial and fungal taxa reported in the phyllosphere. This process involved examining the full text of 269 research papers that specifically analyzed above-ground plant tissues, using a Python script developed for this purpose in this study (https://github.com/isacadavid9-boop/Microbiome-meta-analysis). 

### Plant phyllosphere works data extraction

All records found in the Web of Science database about the plant phyllosphere were sought for retrieval, downloaded, and analyzed using the *Sysrev* platform (Bozada et al., 2021). Each report was reviewed once by the review team. Data extracted consisted of year, title, plant species, tissue analyzed (leaf, stem, seed, flower, fruit, branches or trunk), microbiome analyzed (bacteria, fungi or other), sampling location (continent), growth environment (field, farm, garden, greenhouse), microorganism harvesting method (washing or grinding), DNA extraction protocol, DNA marker, plastid DNA discrimination method, sequencing platform, software for sequence analysis, taxonomic affiliation database and variables. If applicable, we also collected stress conditions and microbiome functional assignment. Data is available in the Table S2.

## Results and Discussion

### Exploring phyllosphere microbiome studies: Search results

To provide a general overview of the proportion of phyllosphere studies within the broader plant microbiome literature, we conducted a search focusing on records that explored microorganisms associated with both below-ground (root and rhizosphere) and above-ground (phyllosphere or leaf) tissues using Next-Generation Sequencing (NGS) methods, as detailed in Table S1 (item a). This search yielded a total of 1,823 articles related to the phyllosphere, rhizosphere, leaf, or root tissues (Figure 1a). A comparison between studies focusing on below-ground (n = 1562, Table S1 item b) and above-ground tissues (n = 375 Table S1 item c) revealed that the latter constituted approximately 20% of the total publications, indicating that research on the phyllosphere remains an underexplored field. Additional searches were conducted to determine the number of studies exclusively focused on each plant region: A total of 225 publications focused exclusively on above-ground tissues, whereas1,361 studies concentrated on below-ground tissues (Figure 1 a ), revealing a strong research bias toward the root-associated microbiome compared to the phyllosphere.

The initial search of phyllosphere microbiome studies yielded 375 records. From these, 42 records were excluded (only abstract was available), therefore 333 reports were retrieved and reviewed. During analysis, 65 reports were excluded and ultimately, 268 reports were included in the final analysis (Figure 1 b ).

### Phyllosphere microbiome: Geographic distribution and dominant microbial groups

**Figure 2 -:**
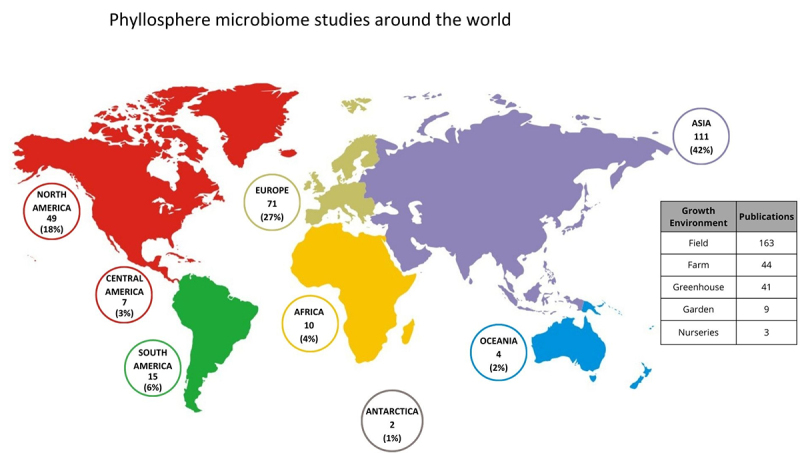
Overview of global phyllosphere studies. The world map shows the distribution of phyllosphere studies by continent (each continent color-coded), including the number of studies and their percentage of the total. To the right, the chart depicts the growth environments of the studied plants, along with the corresponding number of reports**.**

The examination of phyllosphere microbe sampling locations reveals a significant imbalance in the research focus, with 89% of studies concentrating on the Northern Hemisphere, heavily weighted toward Asia (42%) and North America (18%). In contrast, the Southern Hemisphere, comprising South America, Africa, Antarctica, and Oceania, remains underrepresented in the literature (Figure 2). 

These areas likely harbor unique microbial communities shaped by distinct climatic and ecological conditions, that remain poorly characterized. This discrepancy highlights the need for increased investigation in these less-studied regions to enhance the global understanding of phyllosphere microbiomes. 

Based on our revision, samples were typically collected from natural environments (e.g., field and farm) rather than controlled environments (e.g., greenhouse). Field environments are crucial for study because they capture the full complexity and functionality of microbial communities, offering valuable insights into ecological processes, plant resilience, and practical applications for agriculture and conservation (Li et al., 2024 a , b; Siegenthaler et al., 2024). On the other hand, greenhouse studies provide a valuable opportunity to explore and manipulate plant-microbe interactions in a controlled setting, which can help in identifying microbes that promote plant health or suppress plant pathogens, which can lead to improved agricultural practices and better understanding of microbial roles in plant health (Khoiri et al., 2024; Su et al., 2024). Together, these complementary approaches highlight the importance of integrating field realism with experimental control to advance phyllosphere microbiome research and its applications.

After assessing sampling locations, we analyzed the prevalence of key microbial groups to identify those that dominate the phyllosphere at a global scale. Bacteria and filamentous fungi are extensively studied members of the phyllosphere (Figure S1). However, research interest is growing in understanding the community composition of other microbial groups such as yeasts (Gouka et al., 2022), protists (Taerum et al., 2023), and viruses (Forero-Junco et al., 2022). Therefore, our investigation specifically targeted bacteria and fungi in our search strategy (Figure S1a). Through an examination of the entire sections of the publications (except for reference section), we identified the most dominant and abundant bacteria (Table 1) and fungi (Table 2) present in the phyllosphere microbiome. Among bacteria, Proteobacteria, Firmicutes, and Bacteroidota were the most frequently reported phyla, while *Pseudomonas*, *Bacillus*, *Sphingomonas*, and *Methylobacterium* were prominent at the genus level (Figure S2). Despite the underrepresentation of some continents in terms of sampling, we observed that the most dominant bacterial groups were present across all continents, suggesting that even limited sampling can reveal their global prevalence.

**Table 1 -:** Most dominant bacteria in phyllosphere.

	Bacteria	Publications #
**Phylum**	Proteobacteria, Pseudomonadota	120
	Firmicutes, Bacillota	78
	Bacteroidota, Bacteroidetes	78
	Acidobacteria	48
	Cyanobacteria	37
	Actinobacteriota, Actinomycetes, Actinomycetota	30
**Genus**	Pseudomonas	86
	Bacillus	76
	Sphingomonas	58
	Methylobacterium	48
	Rhizobium	44
	Pantoea	38
	Erwinia	28
	Staphylococcus	23
	Microbacterium	19
	Hymenobacter	19
	Klebsiella	18

**Table 2 -:** Most dominant fungi in phyllosphere.

	Fungi	Publications #
Phylum	Ascomycota	77
	Basidiomycota	67
Genus	Candida	51
	Alternaria	50
	Cladosporium	43
	Fusarium	39
	Aspergillus	34
	Penicillium	27
	Aureobasidium	26
	Phoma	24
	Cryptococcus	21
	Trichoderma	14
	Rhodotorula	12
	Lophodermium	3
	Stagonospora	4
	Arthrinium	2

For fungi, Ascomycota and Basidiomycota were dominant globally (Figure S2), with *Candida*, *Alternaria*, and *Cladosporium* as the most frequently reported genera (Table 2). These patterns agree with the findings from Sohrabi et al. (2023) and suggest that Proteobacteria, Firmicutes, Ascomycota, and Basidiomycota function as global phyllosphere generalists, while regional environmental conditions may shape the distribution of other microbial groups. However, the strong emphasis on dominant and abundant taxa in most phyllosphere studies means that rare taxa are often overlooked. Despite their low abundance, these rare microbes can play disproportionately important roles in community stability, functional diversity, and plant health, acting as reservoirs of functional potential, enhancing resilience under stress, and occasionally shifting to dominance when conditions change (Jia et al., 2022; Dong et al., 2024; Shao et al., 2024). In our systematic review, this reporting bias limited the possibility of conducting a robust meta-analysis of plant-specific microbiomes, as the available data primarily reflects the most generalist and widespread taxa. Expanding efforts to systematically document and compare rare taxa will therefore be crucial to uncover host-specific microbial signatures and to advance our understanding of the ecological and functional complexity of the phyllosphere.

### Microbiome assembly is influenced by host plant diversity, tissue types, and phyllosphere compartments

**Figure 3 -:**
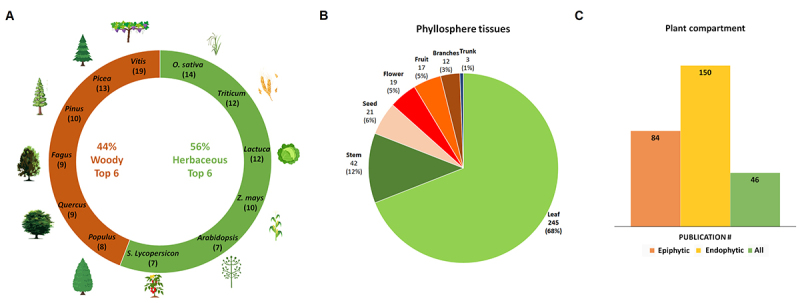
Publications by plant type, species, and phyllosphere compartments. **A**. Percentage of publications for woody and herbaceous plants including the six most-studied species in each category. The numbers in parentheses represent the absolute number of papers related to each species. **B.** Distribution of studies across phyllosphere tissues and compartment dominance in the analyzed studies. **C.** Distribution between epiphytic and endophytic compartments. Numbers represent both the count of studies and their percentage relative to the total.

Plant species and genetics shape the phyllosphere microbiome (Liu et al., 2023; Zhang et al., 2023); consequently, a diverse range of plant groups has been investigated to characterize the associated bacteria and fungi. In this review, herbaceous plants represented a larger proportion of research (56%) compared to woody plants (44%). Our compiled data reflects researchers’ strong interest in crops of high economic importance and experimental tractability (77% of the analyzed studies), such as rice, which dominated among herbaceous species. Likewise, grapevine was the most frequently studied woody plant, underscoring the emphasis placed on species of major agricultural relevance (Figure 3a , Table S3). Other, less-represented plant species are listed in Table S2. To advance our understanding of plant-microbe interactions in the phyllosphere, future research should prioritize diversification in host plant selection. Expanding the scope to include underrepresented plant taxa, especially non-domesticated and wild species, will be crucial. These plants often thrive in hostile or variable environments, and their associated microbiomes may harbor unique adaptive traits that can shed light on microbial contributions to stress tolerance, resilience, and ecological adaptation.

Moreover, the majority of research has concentrated on leaf tissue, which accounts for 68% of the studies, while other plant tissues such as stems, seeds, fruits, and flowers receive less attention. Trunk tissue is notably underrepresented, making up less than 1% of the studies (Figure 3 b ). Additionally, studies on endophytic compartments outnumber those on epiphytic compartments, suggesting that the latter remain comparatively less explored in the current literature, which may indicate opportunities for further research (Figure 3 c ). These tissues and compartments likely support distinct microbial communities with specialized roles that are vital for understanding the full spectrum of above-ground plant-microbe interactions.

Taken together, these patterns point to opportunities for more inclusive, ecologically informed, and tissue-diverse research. Such efforts will not only deepen our fundamental understanding of plant-microbe dynamics but also open new pathways for applied outcomes in sustainable agriculture, conservation, and ecosystem management.

### Diverse approaches to studying the plant phyllosphere microbiome

*Factors influencing phyllosphere microbiome assembly*

**Figure 4 -:**
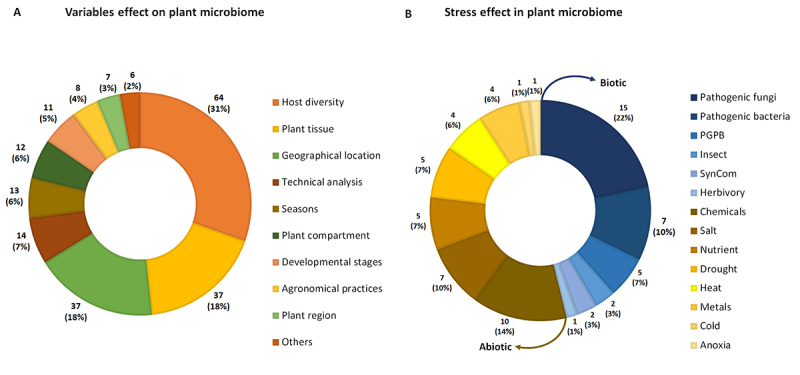
Variables assessed in phyllosphere microbiome studies. **A**. Factors influencing plant microbiome assembly. **B**. Biotic and abiotic stressors affecting plant microbiome.

Recent research has advanced beyond cataloging individual factors to reveal how these drivers interact to shape microbial community structure and function (Figure 4). Among these drivers, host-related traits accounted for 63% of the evaluated variables. These included species identity and genotype (collectively referred to as *host diversity* in Figure 4 a ), as well as tissue type, compartment, and developmental stage, which act as selective filters that regulate which microbes can colonize and persist. For example, plant genotypes can influence the production of secondary metabolites, structural barriers, or signaling molecules, which can selectively promote mutualistic microbes or inhibit pathogens (Chaudhry et al., 2021; Zhang et al., 2023).

Environmental variables (37% of the evaluated variables), including geographical location, seasonal dynamics, and agronomic practices, can interact with host selection, sometimes amplifying its effects (Taguali et al., 2025). Likewise, biotic stressors (46% of the evaluated stresses; Figure 4 b ), such as bacteria and fungi pathogens or herbivores can disrupt established microbial networks, often favoring opportunistic or stress-tolerant taxa. Pathogen pressure tends to reduce diversity but can also stimulate recruitment of beneficial taxa with protective functions (Gao et al., 2021). Abiotic challenges (54% of the evaluated stresses; Figure 4 b ), such as drought, salinity, nutrient imbalances, or heavy metals can impose strong selective pressures, reshaping community composition and functional potential. In response to these stresses, stress-specific microbiota are recruited and exhibit diverse protective functions, such as regulating osmotic pressure, secreting antibiotics, and other mechanisms (Liu et al., 2025). In parallel, manipulative approaches, such as inoculation with Plant Growth-Promoting Bacteria (PGPB) or deployment of Synthetic Communities (SynComs), serve as experimental tools to test causal links and explore microbiome engineering strategies. 

Overall, these studies highlight the complex interplay of multiple variables affecting plant microbiome dynamics and underscore the need for continued research to better understand these interactions. Understanding these multi-layered interactions will be key to designing microbiome-informed approaches for sustainable agriculture, ecosystem restoration, and climate resilience.

### Available tools and methodologies for plant microbiome analysis

*Methodologies for microbiome harvesting*

**Figure 5-:**
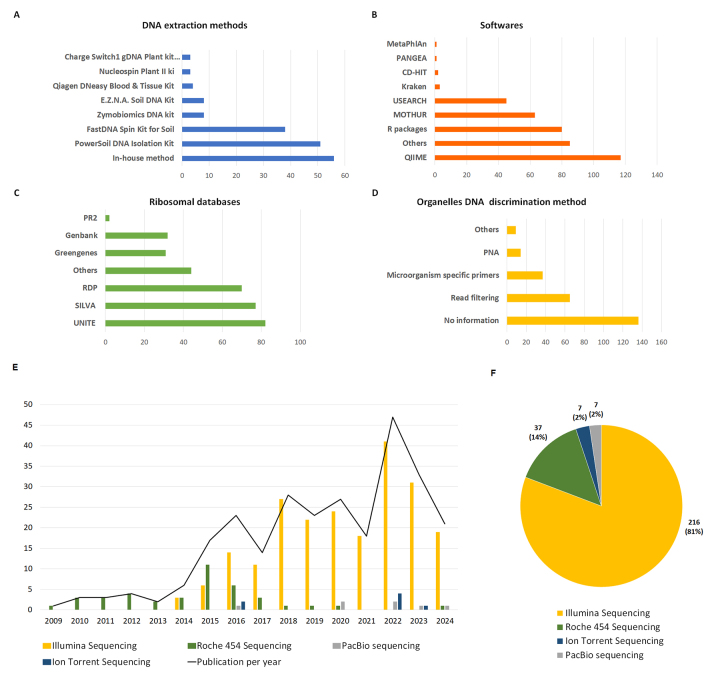
Common methodologies in phyllosphere microbiome studies. **A**. DNA extraction methods; **B**. Software used for sequence analyzes; **C**. Ribosomal databases; **D**. Methods for discriminating organelle DNA; **E**. Usage of sequencing platforms over time (2009-2024); **F**. Number of publications per sequencing platform.

To present the variety of methodologies used in phyllosphere microbiome studies, we extracted and compared key protocols used across literature (Figure 5). Microbial isolation strategies typically target either epiphytic or endophytic communities, or both, but differ in how they balance recovery efficiency and specificity. Epiphytes are commonly isolated by washing plant tissues in a surfactant-containing buffer (e.g., Triton X-100) with agitation to dislodge surface microbes (Dong et al., 2019; Yao et al., 2019; Li et al., 2023 a). In contrast, protocols aiming to recover both epiphytic and endophytic communities often rely on direct grinding of intact tissues(Li *et al*., 2023b), which increases yield but may blur the compartmental distinction. For endophyte-specific analyses, surface sterilization followed by tissue homogenization is standard practice to minimize contamination from surface-associated microbes (Zhou et al., 2024). 

*DNA extraction methods*

Following microbial isolation, a range of DNA extraction methods is employed across phyllosphere studies (Figure 5 a ). Commercial kits such as the PowerSoil™ DNA Isolation Kit (MoBio Laboratories, USA) are widely used, alongside in-house protocols like the cetyl-trimethyl ammonium bromide (CTAB) method. Each approach varies in its efficiency, cost, and capacity to lyse diverse microbial cells. Given the structural variability among microbial taxa, particularly between Gram-positive and Gram-negative bacteria, protocols combining mechanical disruption (e.g., bead-beating) with chemical lysis are generally more effective in maximizing DNA yield and diversity. Additionally, due to the presence of plant-derived compounds and other organic inhibitors in phyllosphere samples, protocols that include steps to reduce these contaminants are critical for downstream sequencing quality (Guo and Zhang, 2013; Yang et al., 2023; Liu et al., 2024). Therefore, the choice of extraction methods can significantly influence the representation of microbial communities and should be aligned with study objectives and sample characteristics.

*Amplicon and shotgun metagenomic analysis*

**Figure 6 -:**
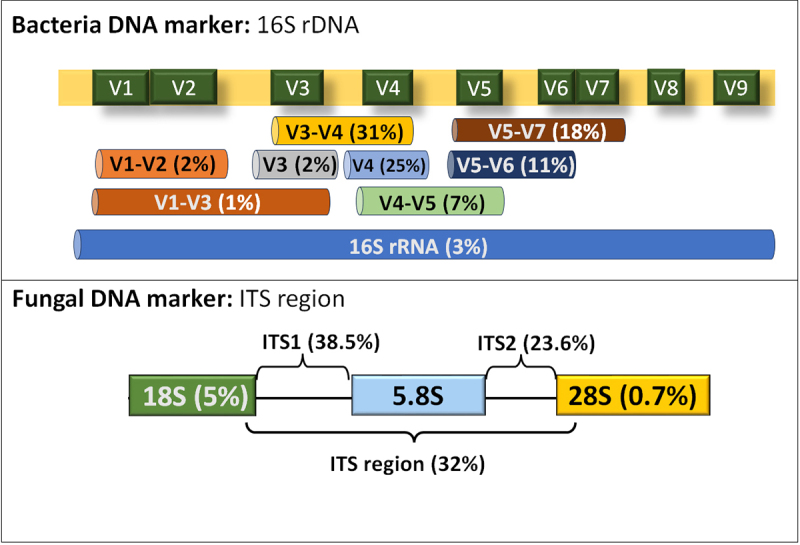
Molecular markers used in metagenomic analyses**. Top:** Bacterial 16S rRNA gene and its nine hypervariable regions (V1-V9) used for bacterial identification. **Bottom:** Eukaryotic ribosomal genes (18S, 5.8S, 28S) and ITS regions used for fungal identification. Percentages indicate the proportion of studies using each marker relative to the total.

The extracted genomic material is typically used for either amplicon-based sequencing or shotgun metagenomic analysis. In amplicon sequencing, the first step involves targeting and amplifying specific genetic markers, most commonly the 16S rRNA gene for bacteria, to enable taxonomic identification and assessment of community diversity. This gene contains nine hypervariable regions (V1-V9), and a series of experimental works has shown that a choice of 16S rRNA region can significantly impact estimates of taxonomic diversity of microbial communities (Yu et al., 2008; Klindworth et al., 2013; Yang et al., 2016; Hrovat et al., 2024). The V3-V4 region is often preferred in phyllosphere studies (31% of studies), followed by V4 (25%), V5-V7 (18%), and V5-V6 (11%) (Figure 6 a ). Despite using a full-length 16S rRNA gene is better for a species-level resolution, this region is less used (3% of reports). Smaller regions have been preferred because increasing read length for Illumina and 454 Life Sciences sequencers results in a decrease in read sequence quality. Each hypervariable region of the 16S rRNA gene has unique characteristics that can be advantageous depending on the specific research objectives. A recent study reported that the resolution of different variable regions varies across plant-associated bacteria and differs among 16 microbial genera (Hrovat *et al*., 2024). Their results indicate that the V1-V3 region is typically a better choice compared to the commonly used V3-V4 region for microbiome analysis in plant-associated genera.

The fungal rRNA is composed of coding and noncoding spacer regions. The coding region consists of 18S, 5.8S and 28S units along with several noncoding regions consisting mainly of internal transcribed spacers (ITSs) and intergenic sequences. ITS variable regions have been the most used gene target for fungal identification. Among ITS regions, ITS1 is utilized most frequently in phyllosphere studies (38.5% of reports), followed by ITS2 (23.6%). The preference for ITS1 is likely due to its superior performance compared to ITS2 in terms of species richness and taxonomic coverage, However, some taxa are detected exclusively by ITS2, and vice versa for ITS1. Neither barcode is perfect for distinguishing all species (Mbareche et al., 2020; Tedersoo et al., 2022). Consequently, the entire ITS region (ITS1-5.8S-ITS2) is also used in phyllosphere studies, as seen in 32% of the studies (Figure 6 b ). 

On the other hand, although the 18S rRNA gene was used for taxonomic purposes in 5% of the reports, ITS sequences are generally preferred for detailed fungal identification and diversity studies because of their higher resolution at the species level (Liu et al., 2015).

According to our review, 5% of the reports used a shotgun metagenomic approach. These kinds of phyllosphere microbiome studies started emerging from 2019. By sequencing the entire community’s DNA, researchers can infer not only the presence of microorganisms but also the metabolic pathways and functions of the microbial community, providing insights into their roles in the environment or host. This approach also offers high-resolution data and can distinguish between closely related species, not only from bacteria and fungi, but also archaea, virus and protists. For functional classification many softwares are available and have been used in this kind of research such as: FUNGuild (Nguyen et al., 2016), PICRUSt2 (Douglas et al., 2020), Piphillin (Narayan et al., 2020), HUMAnN2 (Franzosa et al., 2018) and Prokka (Seemann, 2014).

Overall, despite the growing interest in whole-genome data to explore the complexity of microbial communities, amplicon-based metagenomic studies remain a cost-effective method for understanding microbiome communities associated with the phyllosphere.

*DNA sequencing platforms*

Following DNA extraction and amplification, various sequencing platforms have been employed to analyze phyllosphere microbiomes, each with distinct advantages and limitations (Figure 5 e  and f). Early studies relied on Roche’s 454 pyrosequencing, which offered relatively long reads (~800 bp) but suffered from high error rates and high costs, leading to its discontinuation in 2016 (Tamaki et al., 2011). Since then, Illumina has become the predominant platform due to its high accuracy, cost-efficiency, and deep sequencing capacity, despite its short-read limitations (~150-300 bp), which can hinder taxonomic resolution of closely related taxa (Logares et al., 2014). Alternatives like Ion Torrent and PacBio have been adopted in fewer studies. Ion Torrent offers rapid sequencing and lower cost, but its susceptibility to indel errors reduces accuracy for some applications. In contrast, PacBio generates long, high-fidelity reads, enabling full-length 16S rRNA sequencing and improved taxonomic resolution, albeit at a higher cost and lower throughput. While less commonly used, these long-read platforms are valuable for resolving complex or novel microbial lineages that short-read technologies may miss (Loman et al., 2012).

To enhance the ecological interpretation of phyllosphere communities, future studies should consider integrating long-read sequencing strategies-such as PacBio or emerging Oxford Nanopore technologies-which allow for improved taxonomic and functional profiling. Combining these with high-throughput short-read platforms may offer a balanced approach to maximize both resolution and cost-effectiveness in microbiome research.

*Computational analysis*

Sequence pre-processing and analysis in phyllosphere microbiome studies typically involves a series of steps, including quality filtering (e.g., removal of low-quality, chimeric, and host organelle-origin reads), clustering into operational taxonomic units (OTUs) or denoising to generate amplicon sequence variants (ASVs), taxonomic classification, and diversity analysis. While these steps were once performed with a suite of separate tools, integrated pipelines such as QIIME2 and Mothur have become widely adopted, used in 30% and 17% of studies, respectively. R-based workflows (e.g., DADA2, Phyloseq) are also increasingly common, appearing in 19% of reports (Figure 5 b ). These tools streamline processing and improve reproducibility, although they differ in terms of flexibility, user interface, and community support.

The choice of reference databases is a critical factor that significantly influences taxonomic resolution and downstream phylogenetic interpretations. SILVA is the most widely used for bacterial identification due to its frequent updates and manual curation based on phylogenetic trees, making it a preferred choice in QIIME2. In contrast, Greengenes, though still popular for its integration in established pipelines, has not been updated since 2013, raising concerns about its taxonomic relevance. The Ribosomal Database Project (RDP) also supports bacterial and archaeal classification but may lack full-length gene coverage. For fungal communities, UNITE is the primary reference database, complemented by repositories such as GenBank and BOLD for ITS sequences (Figure 5 c ). The NCBI Taxonomy database offers a standardized nomenclature across kingdoms but may lack phylogenetic depth compared to specialized databases. Ultimately, the selection of database affects not only taxonomic assignments but also alpha and beta diversity estimates, given differences in reference sets and tree construction methods (Ceccarani and Severgnini, 2023). Regularly updated, curated databases are therefore essential for producing taxonomically accurate and phylogenetically meaningful results.

A particular challenge in plant-associated microbiome studies is the unintended amplification of plant organelle sequences, due to primer binding site conservation between bacterial genomes and chloroplast or mitochondrial DNA. Similarly, ITS primers can sometimes amplify both fungal and plant DNA. Studies have adopted several strategies to mitigate this bias: 28% used organism-specific primers during PCR, 22% employed peptide nucleic acid (PNA) clamps to block host DNA, and 42% relied on post-sequencing bioinformatic filtering of organelle-derived reads after taxonomic classification (Figure 5 d ). These combined approaches enhance microbial signal specificity and improve the reliability of downstream analyses.

*OTUs and ASVs based methods*

To analyze biodiversity from amplicon sequencing data, researchers often start by aggregating over 50,000 paired reads into sequences, such as those produced by Illumina MiSeq. These sequences are then grouped into clusters known as OTUs (Operational Taxonomic Units), typically defined by a 97% sequence identity threshold. This clustering approach helps reduce the dataset size and computational resources required for analysis, while also mitigating the impact of sequencing errors by merging erroneous sequences with correct ones.

Recently, there has been a shift towards using denoising methods that produce exact amplicon sequence variants (ASVs) rather than OTUs (Callahan et al., 2017; Caruso et al., 2019; Fasolo et al., 2024), since the development of algorithms such as DADA2 in 2016 (Callahan *et al*., 2016). These algorithms minimize the sequencing errors, and offers improved resolution and reproducibility over traditional OTU-based methods. Denoising techniques create an error model based on sequencing quality to differentiate true biological variation from sequencing errors. ASVs represent sequences that differ by as little as a single nucleotide, aiming to offer higher accuracy compared to the 97% identity OTUs. However, it is important to note that ASVs are not the same as “100% OTUs” due to differences in their construction methods (Chiarello et al., 2022).

Despite the advantages of ASVs, our review found that phyllosphere microbiome studies still predominantly use OTU-based methods (80%) over ASV-based approaches (15%), likely to facilitate comparison across earlier studies (Figure S1). OTUs have significantly contributed to building our current understanding of plant-associated microbiomes. However, given the growing body of evidence favoring ASVs for their higher resolution and reproducibility, we recommend that researchers carefully consider these findings when selecting their methodological approach. 

Both Fasolo et al. (2024) and Chiarello et al. (2022) highlight the significant impact of choosing between ASV- and OTU-based approaches in microbiome studies. Fasolo *et al*. (2024) demonstrated that OTU clustering, particularly at the traditional 97% threshold, underestimates species richness and alters ecological interpretations by introducing artificial patterns in alpha, beta, and gamma diversities. In contrast, ASVs provide higher resolution, more accurate diversity estimates, and better preserve ecological relationships. Complementing this, Chiarello *et al*. (2022) showed that the choice between OTUs and ASVs has a stronger effect on diversity metrics than other common preprocessing decisions, such as rarefaction or clustering thresholds. They emphasize that OTU methods inflate richness by generating spurious rare units, whereas ASV pipelines offer more consistent and biologically meaningful outputs. Together, these studies strongly support the transition toward ASV-based methodologies for robust and reproducible microbiome analyses (Callahan et al., 2017; Caruso et al., 2019; Fasolo *et al*., 2024).

## Perspectives

This systematic review provides a comprehensive overview of current research and highlights areas needing further investigation. Notably, there is a relative lack of studies focusing on above-ground tissues compared to below-ground tissues. Future research should therefore place greater emphasis on the microbiomes of phyllosphere components, such as leaves, stems, branches, trunks, fruits, and flowers, as these may host diverse microbial communities with distinct ecological functions. Additionally, there is a significant lack of research from the Southern Hemisphere. Future studies should aim to include a wider range of geographical locations to achieve a more global understanding of phyllosphere microbiomes. This broader approach will help uncover region-specific microbial communities and their interactions with the environment. While considerable research has concentrated on economically significant plants such as grapevines, rice, wheat, and wood-producing species, investigating microbes associated with other plant species in conserved ecosystems could yield valuable insights into microbial biodiversity. Furthermore, future studies should pay greater attention to rare microbial taxa, which are often overlooked in studies focusing on dominant and abundant species. Despite their low abundance, rare taxa can play critical roles in community stability, functional diversity, and host-specific interactions, and incorporating them into analyses could reveal unique microbial signatures and ecological functions that are otherwise missed. 

Current phyllosphere microbiome studies predominantly focus on community diversity and composition using amplicon sequencing methods. However, there is a need for more research into the functional roles of key bacterial and fungal species identified in these studies, utilizing shotgun sequencing methods. Incorporating multi-omics approaches, such as combining metagenomics with metabolomics and transcriptomics, could provide a more comprehensive view of the phyllosphere microbiome and its interactions with the plant host. Furthermore, with the recent increase in the use of ASV-based methods, it is important for future studies to compare ASV and OTU-based approaches in various contexts to assess their effectiveness and accuracy. This comparison will aid in standardizing methodologies for phyllosphere microbiome research. Addressing these areas will enhance our understanding of phyllosphere microbiomes and their roles in plant health and ecosystem dynamics.

## Supplementary material

Table S1 - Search strategy used to retrieve plant microbiome records in Web of Science database

Table S2 - Collected data from the included studies in the review.

Table S3 - Studied plants in terms of phyllosphere microbiome.

Figure S1 - Distribution of studied microorganisms and analytical approaches in phyllosphere microbiome research.

Figure S2 - Global distribution of the most dominant bacterial and fungal taxa in the phyllosphere across continents.

## Data Availability

The complete dataset supporting the findings of this study has been published in the article and in the ‘Supplementary Materials’ section
