# Supplementary material for: Phyllosphere microbiomes uncovered: Research trends, geographic disparities, and key microbial players
Source: Genet Mol Biol. 2026 Jan 23;49(Suppl 1):e20250083. doi: 10.1590/1678-4685-GMB-2025-0083 (PMC12893196; doi:10.1590/1678-4685-GMB-2025-0083)
Supplement: Table S1 - [file 1415-4757-GMB-49-s1-e20250083-s1.pdf]

Genetics and Molecular Biology
https://doi.org/10.1590/1678-4685-GMB-2025-0083

Supplementary Material to: Phyllosphere microbiomes uncovered: Research trends, geographic disparities, and key microbial players

Table S1. Search strategy for finding plant microbiome records in Web of Science database.

Topic	*Search Strategy*	*Query*	*Articles (#) in April 2025*	*link*
**(a) Microorganisms associated with above-ground or below-ground tissues** and identified by NGS	Microbiome + Phyllosphere or Rhizosphere or root + DNA sequencing	*((TS=(microbiome OR microbiota OR microbial communit* OR bacterial communit* OR fungal communit*)) AND (TS=(phyllospher* OR phylloplane OR rhizospher* OR rhizoplane) OR (TS=(leaf OR leaves OR root) AND TS=(epiphyt* OR endophyt*))) AND ALL=(NGS OR Illumina OR "454 pyrosequencing" OR "Roche 454" OR reads OR "DNA sequenc*"))*	1823	https://www.webofscience.com/wos/woscc/summary/20fbe880-ccac-402f-b348-8c6d1b0d9410-01574d1c58/relevance/1

**Topic**	***Search Strategy***	***Query***	***Articles (#) in April 2025***	***link***
**(b) Above-ground tissues** (may be have below-ground)	Microbiome + Phyllosphere + DNA sequencing	*((TS=(microbiome OR microbiota OR microbial communit* OR bacterial communit* OR fungal communit*)) AND (TS=(phyllospher* OR phylloplane) OR (TS=(leaf OR leaves) AND TS=(epiphyt* OR endophyt*))) AND ALL=(NGS OR Illumina OR "454 pyrosequencing" OR "Roche 454" OR reads OR "DNA sequenc*"))*	375	https://www.webofscience.com/wos/woscc/summary/189caa21-6e72-4e60-a2ac-1d427d266c58-01574d1795/relevance/1
**(c) Below-ground tissues** (may be have above-ground)	Microbiome + Rhizosphere or root + DNA sequencing	*((TS=(microbiome OR microbiota OR microbial communit* OR bacterial communit* OR fungal communit*)) AND (TS=(rhizosphere* OR rhizoplane) OR (TS=(root) AND TS=(epiphyt* OR endophyt*))) AND ALL=(NGS OR Illumina OR "454 pyrosequencing" OR "Roche 454" OR reads OR "DNA sequenc*"))*	1562	https://www.webofscience.com/wos/woscc/summary/b4fdf427-b591-415c-bdbd-4d61177dbb9b-01574d9343/relevance/1

Topic	*Search Strategy*	*Query*	*Articles (#) in April 2025*	*link*
**(d) Only above-ground** (excluding below-ground)	Microbiome + Phyllosphere + DNA sequencing + without (Rhizosphere or root)	*((((TS=(microbiome OR microbiota OR microbial communit* OR bacterial communit* OR fungal communit*)) AND (TS=(phyllospher* OR phylloplane) OR (TS=(leaf OR leaves) AND TS=(epiphyt* OR endophyt*))) AND ALL=(NGS OR Illumina OR "454 pyrosequencing" OR "Roche 454" OR reads OR "DNA sequenc*"))) NOT ALL=(root)) NOT ALL=(rhizosphere)*	225	https://www.webofscience.com/wos/woscc/summary/4f89ae5a-b185-4600-a72c-af7490ad85b6-01574d81e6/relevance/1
**(e) Only below-ground** (excluding above-ground)	Microbiome + Rhizosphere or root + DNA sequencing + without Phyllosphere	*((TS=(microbiome OR microbiota OR microbial communit* OR bacterial communit* OR fungal communit*)) AND (TS=(rhizospher* OR rhizoplane) OR (TS=(root) AND TS=(epiphyt* OR endophyt*))) AND ALL=(NGS OR Illumina OR "454 pyrosequencing" OR "Roche 454" OR reads OR "DNA sequenc*"))) NOT TS=(leaf)) NOT*	1361	https://www.webofscience.com/wos/woscc/summary/507c053e-3cf1-45e7-bb60-852cc19827be-01574d8f5a/relevance/1

Topic	*Search Strategy*	*Query*	*Articles (#) in April 2025*	*link*
		TS=(leaves)) NOT TS=(phyllosphere)		
**(f) Above-ground not soil** (excluding below-ground and soil)	Microbiome + Phyllosphere + DNA sequencing + without (Rhizosphere or root or soil)	*((((((TS=(microbiome OR microbiota OR microbial communit* OR bacterial communit* OR fungal communit*)) AND (TS=(phyllospher* OR phylloplane) OR (TS=(leaf OR leaves) AND TS=(epiphyt* OR endophyt*))) AND ALL=(NGS OR Illumina OR "454 pyrosequencing" OR "Roche 454" OR reads OR "DNA sequenc*"))) NOT TS=(root)) NOT TS=(rhizosphere)) NOT TS=(rhizoplane)) NOT TS=(soil)*	170	https://www.webofscience.com/wos/woscc/summary/9af462d9-16fa-4170-8c68-bda9fb7b8c9c-01574d974b/relevance/1

**Topic**	***Search Strategy***	***Query***	***Articles (#) in April 2025***	***link***
**(g) Above-ground and Below-ground tissues**	Microbiome+ Phyllosphere + DNA sequencing + Rhizosphere or root	*((TS=(microbiome OR microbiota OR microbial communit* OR bacterial communit* OR fungal communit*)) AND (TS=(phyllospher* OR phylloplane) OR (TS=(leaf OR leaves) AND TS=(epiphyt* OR endophyt*))) AND ALL=(NGS OR Illumina OR "454 pyrosequencing" OR "Roche 454" OR reads OR "DNA sequenc*")) AND TS=(rhizospher* OR rhizoplane OR root)*	149	https://www.webofscience.com/wos/woscc/summary/69933da0-4366-4bf6-9546-a9a9e8f056ff-01574d9acf/relevance/1
