# Supplementary material for: Phyllosphere microbiomes uncovered: Research trends, geographic disparities, and key microbial players
Source: Genet Mol Biol. 2026 Jan 23;49(Suppl 1):e20250083. doi: 10.1590/1678-4685-GMB-2025-0083 (PMC12893196; doi:10.1590/1678-4685-GMB-2025-0083)
Supplement: Table S2 - [file 1415-4757-GMB-49-s1-e20250083-s2.pdf]

Genetics and Molecular Biology
https://doi.org/10.1590/1678-4685-GMB-2025-0083

Supplementary Material to: Phyllosphere microbiomes uncovered: Research trends, geographic disparities, and key microbial players

Table S2 - Collected data from the included studies in the review.

Article ID Article URL User Name Stem type Life cicle Species Tissue Plant compartment Location Growth environment Stress Microbiome analyzed Bacterial DNA marker Shotgun Metagenomic Fungal DNA marker Microorganism isolation method DNA extraction protocol Sequencing platform Software for sequence analysis Taxonomic affiliation database Exclusion criteria Plant DNA discrimination method Functional assignment software Co-occurrence network Variables Network analysis software Year of publication Hightlights Sequence Analysis approach ITS primers name Title Authors

13632122 https://www.sysrev.com/p/104267/article/13632122 erika_frydrych Woody Perenne Theobroma cacaoL Leaf Epyphitic South America Greenhouse None Bacteria 16S rDNA V3 - V4 NO None Wash the tissue PowerSoil DNA Isolation Kit Illumina Sequencing QIIME Greengenes database None Microorganism specific primers None NO two different genotypes (catongo/susceptible and CCN51/resistant None 2018 Genotype CCN51 presented greater diversity of microorganisms in comparison to the Catongo genotype and the total community was different between both OTUs None Witches_ broom resistant genotype CCN51 shows greater diversity of symbiont bacteria in its phylloplane than susceptible genotype catongo.pdf Oliveira, Juliano; Peres Gramacho, Karina; Tícila De Souza, Katiúcia; Ferreira, Eduvirgens; Rezende, Rachel Passos.; Antônio, Pedro; Mangabeira, Oliveira; Pedro, Ricardo; Dias, Moreira; Couto, Francisco M.; Pirovani, Carlos Priminho.

13632124 https://www.sysrev.com/p/104267/article/13632124 carolinesalvati Woody Perenne Abies sp.|||Alnus sp.|||Larix sp.|||Picea sp. Leaf Epyphitic North America Field None Bacteria 16S rDNA V3 - V4 NO None Wash the tissue PowerSoil DNA Isolation Kit Illumina Sequencing QIIME||| Others SILVA database None read filtering None NO Plant species|||Geography None 2017 We show that only a small fraction (13%) of all detected OTUs constitute a metacommunity seed bank that is shared between all terrestrial and aquatic communities, but not by phyllosphere assemblages, which seem to recruit from a different taxa pool. Identifying the core seed bank of a complex boreal bacterial metacommunity.pdf Ruiz-González, Clara; Niño-García, Juan Pablo.; Kembel, Steven W.; Del Giorgio, Paul A.

13632126 https://www.sysrev.com/p/104267/article/13632126 carolinesalvati Woody Perenne Paeonia Sect. Moutan Root||| Leaf Endophitic Asia Garden None Bacteria 16S rDNA V3 - V4 NO None Grind the tissue Others Illumina Sequencing QIIME Ribosomal Database Project None read filtering None NO Plant Cultivars|||Plant tissue None 2017 both plant genotype and tissues contribute to the shaping of the bacterial communities associated with tree peony Illumina-based analysis of endophytic bacterial diversity of tree peony (Paeonia Sect. Moutan) roots and leaves.pdf Yang, Ruixian; Liu, Ping; Ye, Wenyu; Andreotte, Fernando

13632127 https://www.sysrev.com/p/104267/article/13632127 erika_frydrych Herbaceous Perenne Huperzia serrata Root||| Stem||| Leaf Endophitic Asia Field None Fungi none NO ITS2 Grind the tissue Others Illumina Sequencing QIIME||| R packages UNITE database None Microorganism specific primers None NO Different tissues None 2020 Fungal

communities are tissue-type and tissue-site specific OTUs Illumina-based analysis yields new insights into the diversity and composition of endophytic fungi in cultivated Huperzia serrata.pdf

13632128 https://www.sysrev.com/p/104267/article/13632128 carolinesalvati Woody Perenne Cynnamomum camphora Leaf Epyphitic Asia Field Chemicals Bacteria||| Fungi 16S rDNA V3 - V4 NO ITS Wash the tissue FastDNA Spin Kit for Soil Illumina Sequencing Others Greengenes database||| UNITE database None None None NO PAHs contamination level|||Ecosystem None 2022 The composition of Phyllosphere differed between the three urban categories which can be largely explained by the variation of phyllosphere PAH concentration and the surrounding land-use types Immune-mediated disease associated microbial community responded to PAH stress in phyllosphere of roadside greenspaces in Shanghai.pdf Tan, Haoxin; Liu, Xinxin; Yin, Shan; Zhao, Chang; Su, Lantian; Li, Xiaoxiao; Khalid, Muhammad; Setälä, Heikki; Hui, Nan

13632129 https://www.sysrev.com/p/104267/article/13632129 erika_frydrych Herbaceous Annual SpinaceaOleracea Leaf Epyphitic Europe Field None Bacteria 16S rDNA V3 - V4 NO None Others FastDNA Spin Kit for Soil Illumina Sequencing QIIME Greengenes database None None None NO . None 2018 . OTUs . Impact of chlorine dioxide disinfection of irrigation water on the epiphytic bacterial community of baby spinach and underlying soil.pdf Truchado, Pilar; ´a, Marı; Gil, Isabel; Suslow, Trevor; Allende, Ana

13632130 https://www.sysrev.com/p/104267/article/13632130 erika_frydrych Herbaceous Perenne Hedera helix|||Hedera hibernica Leaf Epyphitic Europe Other None Bacteria 16S rDNA V4 NO None Wash the tissue Others Illumina Sequencing R packages Greengenes database None read filtering None NO the effect of an urban environment on the bacterial composition None 2016 OTU richness and diversity of the phyllosphere bacterial community of ivy are alike at urban and non-urban locations, but their species composition differs significantly; Atmospheric particula matter exposure is an important driver of the community composition OTUs Impact of urban land use on the bacterial phyllosphere of ivy (Hedera sp.).pdf Smets, Wenke; Wuyts, Karen; Oerlemans, Eline; Wuyts, Sander; Denys, Siegfried; Samson, Roeland; Lebeer, Sarah

13632131 https://www.sysrev.com/p/104267/article/13632131 erika_frydrych Herbaceous Annual Spinacia oleracea Leaf All North America Field Salt Bacteria 16S rDNA V4 NO None Wash the tissue PowerSoil DNA Isolation Kit Illumina Sequencing QIIME||| MOTHUR||| USEARCH||| R packages Greengenes database None read filtering Piphillin NO seasonal effects and temporal variabillities driving bacterial community composition and function|||increasing salinity and season None 2021 microbiomes were significantly affected by seadon, but not salinity OTUs None Influence of seasonal changes and salinity on spinach phyllosphere bacterial functional assemblage.pdf

13632132 https://www.sysrev.com/p/104267/article/13632132 carolinesalvati Herbaceous Perenne Solanum lycopersicum Fruit||| Flower Epyphitic North America Farm Biotic Bacteria 16S rDNA V1 - V3 NO None Wash the tissue Others Illumina Sequencing Others||| QIIME Greengenes database||| Ribosomal Database Project None read filtering None NO Plant tissue|||Insect exclusion None 2018 Insects may introduce or augment variability in bacterial diversity associated with tomato flowers and potentially green fruit surfaces Insect exclusion limits variation in bacterial microbiomes of tomato flowers.pdf Allard, S M.; Ottesen, A R.; Brown, E W.; Micallef, Shirley A.

13632135 https://www.sysrev.com/p/104267/article/13632135 carolinesalvati Herbaceous Perenne Brassica oleracea var.italica Flower Epyphitic Asia Farm||| Other None Bacteria 16S rDNA V5 - V6 NO None Wash the tissue PowerSoil DNA Isolation Kit Roche 454 Sequencing QIIME Greengenes database None Microorganism specific primers None NO Cultivation method|||Pre/post-harvest None 2018 the dominant bacterial taxa persist and prevail in the phyllosphere of broccoli during the postharvest stage Postharvest processing decreases the richness of bacterial taxa in the phyllosphere of broccoli.pdf Kim, M.-S; Bae, J.-W; Park, E.-J; Park, Eun-Jin; Bae, Jin-Woo

13632139 https://www.sysrev.com/p/104267/article/13632139 erika_frydrych Herbaceous Perenne Cucurbita moschata Leaf Epyphitic Asia Field Biotic Fungi none NO ITS1 Wash the tissue FastDNA Spin Kit for Soil Illumina Sequencing R packages||| Others||| MOTHUR UNITE database None Microorganism specific primers None NO different severity of powdery mildew disease None 2018 The pathogen caused an increase in the fungal community richness at first and then a decrease when disease pressure was higher OTUs Pumpkin powdery mildew disease severity influences the fungal diversity of the phyllosphere.pdf Zhang, Zhuo; Luo, Luyun; Tan, Xinqiu; Kong, Xiao; Yang, Jianguo; Wang, Duanhua; Zhang, Deyong; Jin, Decai; Liu, Yong

13632140 https://www.sysrev.com/p/104267/article/13632140 carolinesalvati Herbaceous Annual Oryza sativa subsp. japonica) Leaf Endophitic Asia Farm Nutrient||| Anoxia Bacteria 16S rDNA V4 - V5 NO None Grind the tissue||| Wash the tissue In-house method Roche 454 Sequencing QIIME Ribosomal Database Project None read filtering None NO Plant development stage|||Nitrogen

fertilization condition None 2015 The difference in the bacterial community structure between the different growth stages was greater than the difference resulting from the elevated CO 2 levels and nitrogen fertilization treatments. Response of leaf endophytic bacterial community to elevated CO2 at different growth stages of rice plant.pdf Hazard, Christina; Jia, Zhongjun; Ren, Gaidi; Zhang, Huayong; Lin, Xiangui; Zhu, Jianguo

13632141 https://www.sysrev.com/p/104267/article/13632141 carolinesalvati Herbaceous Annual Sinapis alba Leaf||| Rhizosphere||| Stem|||Root Epyphitic||| Endophitic Europe None Biotic||| Metals Bacteria 16S rDNA V5 - V6 NO None Grind the tissue PowerSoil DNA Isolation Kit||| In-house method Illumina Sequencing Others SILVA database None Microorganism specific primers None NO Plant growth-promoting bacteria (Pseudomonas sp.) None 2020 Bioaugmentation of the soil caused a diverse shift in the bacterial communities in the rhizo- and endo-sphere of white mustard compared to control Response of rhizospheric and endophytic bacterial communities of white mustard (Sinapis alba) to bioaugmentation of soil with the Pse.pdf Płociniczak, Tomasz; Pacwa-Płociniczak, Magdalena; Kwaśniewski, Mirosław; Chwiałkowska, Karolina; Piotrowska-Seget, Zofia

13632142 https://www.sysrev.com/p/104267/article/13632142 erika_frydrych Herbaceous Annual Oryza sativa Leaf||| other Endophitic Asia Field heat Bacteria 16S rDNA V4 - V5 NO None Grind the tissue FastDNA Spin Kit for Soil Roche 454 Sequencing QIIME||| USEARCH||| Others||| R packages Ribosomal Database Project None Microorganism specific primers None NO Response of soil, leaf endosphere and phyllosphere bacterial communities to elevated CO2|||Responde to soil temperature None 2015 Soil bacterial communities were more resistant to the tested climate change factors compare with foliar bacterial communities; Temperature was more important in shaping the structure of foliar bacterial communities compared with CO2 . OTUs Response of soil, leaf endosphere and phyllosphere bacterial communities to elevated CO2 and soil temperature in a rice paddy.pdf Ren, Gaidi; Zhu, Chunwu; Saiful, M; Tokida, Takeshi; Sakai, Hidemitsu; Nakamura, Hirofumi; Usui, Yasuhiro; Zhu, Jianguo; Hasegawa, Toshihiro; Jia, Zhongjun; Alam, M S.; Sakai, : H.

13632144 https://www.sysrev.com/p/104267/article/13632144 carolinesalvati Herbaceous Annual Fragaria ananassa Leaf Epyphitic Asia Farm||| Greenhouse Biotic Bacteria||| Fungi 16S rDNA V3 - V4 NO ITS Wash the tissue PowerSoil DNA Isolation Kit Illumina Sequencing Others SILVA database||| UNITE database None None None NO Ecosystem|||Biologia Control Agent (Bacillus subtilis) None 2016 Phyllosphere microbiota differed significantly between plants grown in open field and under protection but were not greatly affected by introduced BCA Dispersal of Bacillus subtilis and its effect on strawberry phyllosphere microbiota under open field and protection conditions.pdf Wei, Feng; Hu, Xiaoping; Xu, Xiangming

13632145 https://www.sysrev.com/p/104267/article/13632145 erika_frydrych Herbaceous Perenne Gentiana rigescens Leaf||| Stem||| Flower|||Root Endophitic Asia Other None Fungi none NO ITS Wash the tissue PowerSoil DNA Isolation Kit Roche 454 Sequencing QIIME||| R packages UNITE database None None None NO culture-dependent and culture-independent techniques|||e cytotoxicity of endophytic fungi against four human cancer cell lines None 2020 Activity assays indicated that endophytes were a promising natural source of potential anticancer agents OTUs ITS5 and ITS4 Distribution and diversity of endophytic fungi in Gentiana rigescens and cytotoxic activities.pdf Xu, Li-Li; Ling, Xiao-Feng; Zhao, Shu-Juan; Wang, Ru-Feng; Wang, Zheng-Tao

13632146 https://www.sysrev.com/p/104267/article/13632146 erika_frydrych Herbaceous Annual Vicia faba Root||| Leaf Endophitic Europe Field None Bacteria 16S rDNA V3 NO None Others DNeasy® PowerPlant® Pro KiIllumina Sequencing R packages SILVA database None Others None NO ; None 2018 ; OTUs ; Distribution of antibiotic resistance genes in soils and crops. A field study in legume plants (Vicia faba L.) grown under different.pdf Cerqueira, Francisco; Matamoros, Víctor; Bayona, Josep; Elsinga, Goffe; Hornstra, Luc M.; Piña, Benjamin

13632148 https://www.sysrev.com/p/104267/article/13632148 erika_frydrych Woody Perenne Fagus sylvatica Leaf Endophitic Europe Field None Fungi none NO ITS Wash the tissue Charge Switch1 gDNA Plant kit (Invitrogen) Illumina Sequencing R packages UNITE database None None None NO two different altitudes in the German Alps and from a managed tree nursery None 2016 Diversity and composition correlated significantl; n the mycobiome was more diverse at lower than at higher elevation, whereas fungal diversity was lowest in the artificial habitat of the tree nursery; OTUs V9G (de Hoog GS et al., 1998) and ITS4 (White et al 1990) Diversity and Composition of the Leaf Mycobiome of Beech (Fagus sylvatica) Are Affected by Local Habitat Conditions and Leaf Biochemistry.pdf Unterseher, Martin; Siddique, Abu Bakar.; Brachmann, Andreas; Peršoh, Derek

13632149 https://www.sysrev.com/p/104267/article/13632149 isacadavid9 Herbaceous Perenne Limonium sinense Leaf|||Root||| Rhizosphere||| Bulk soil Endophitic Asia Field Salt Bacteria 16S rDNA V5 - V7 NO None Grind the tissue PowerSoil DNA Isolation Kit Illumina Sequencing QIIME||| MOTHUR SILVA database None Microorganism specific primers None NO Plant tissue None 2018 We identified 49 representative bacterial strains belonging to 17 genera across all samples, with Glutamicibacteras the most dominant , enus. All Glutamicibacterisolates showed

multiple potential plant growth pro OTUs None Diversity of Bacterial Microbiota of Coastal Halophyte Limonium sinense and Amelioration of Salinity Stress Damage by Symbiotic Plant.pdf Qin, Sheng; Feng, Wei-Wei; Zhang, Yue-Ji; Wang, Tian-Tian; Xiong, You-Wei; Xing, Ke

13632150 https://www.sysrev.com/p/104267/article/13632150 erika_frydrych Herbaceous Perenne Artemisia argyi Leaf Endophitic Asia Field None Fungi none NO ITS1 Grind the tissue Others Illumina Sequencing MOTHUR NCBI None Microorganism specific primers FUNGuild NO five A. argyi varieties|||grown in different cultivation areas None 2021 QA and BA had rich fungal community structure and diversity, presenting differences in the fungal species and distribution. the majority of the fungal species were detected in the leaves of A. argyi, with few major pathogenic fungi and very few beneficial biocontrol fungi. OTUs Diversity of Endophytic Fungal Community in Leaves of Artemisia argyi Based on High-throughput Amplicon Sequencing.pdf

13632153 https://www.sysrev.com/p/104267/article/13632153 erika_frydrych Herbaceous Perenne Atractylodes macrocephala Bulk soil|||Root||| Stem||| Leaf||| other All Asia Field None Fungi none NO ITS1 Wash the tissue E.Z.N.A. Soil DNA Kit Illumina Sequencing Others||| USEARCH Ribosomal Database Project None None None NO The effects of continuous cropping on the endophytic and rhizospheric fungi None 2020 Continuous cropping was found to decrease fungal diversity inside plant roots, stems, leaves and tubers; The structure and diversity of rhizospheric and endophytic fungal communities were altered by root-rot disease OTUs ITS1 -Fand ITS2-F Diversity of rhizosphere and endophytic fungi in Atractylodes macrocephala during continuous cropping.pdf Zhang, Qiaoyan; Qin, Luping; Aramayo, Rodolfo; Zhu, Bo; Wu, Jianjun; Ji, Qingyong; Wu, Wei; Dong, Shihui; Yu, Jiayan

13632154 https://www.sysrev.com/p/104267/article/13632154 erika_frydrych Woody Annual Vitis labruscana Leaf Endophitic North America Field None Bacteria||| Fungi 16S rDNA V3 - V4 NO 18S rRNA Others PowerSoil DNA Isolation Kit Illumina Sequencing R packages SILVA database||| UNITE database None None PICRUSt NO . None 2018 . OTUs . “Concord” grapevine nutritional status and chlorosis rank associated with fungal and bacterial root zone microbiomes.pdf Lewis, R W.; Letourneau, M K.; Davenport, J R.; Sullivan, Tarah S.

13632155 https://www.sysrev.com/p/104267/article/13632155 erika_frydrych Woody Annual Fagus sylvatica Leaf Endophitic Europe Field None Fungi none NO ITS Grind the tissue Charge Switch1 gDNA Plant kit (Invitrogen) Illumina Sequencing QIIME||| USEARCH||| R packages UNITE database None None None NO The impact of habitat and substratum conditions on leaf-inhabiting fungal communities.|||Three different altitudes None 2016 Significant correlation of community composition with elevation was observed; The mycobiome was little affected by the physiological state of the leaves, because only a partial shift of taxonomic composition was observed from vital towards clearly senescent leaves OTUs ITS1F/ITS4 (White et al., 1990) and V9G/ITS4 (de Hoog and Gerrits van den Ende, 1998) A cost-effective and efficient strategy for Illumina sequencing of fungal communities A case study of beech endophytes identified ele.pdf Siddique, A B.; Unterseher, M; Orn, Bj€; Lindahl, D; Ernst-Moritz-Arndt

13632156 https://www.sysrev.com/p/104267/article/13632156 erika_frydrych Herbaceous Perenne Acanthus ilicifolius Leaf Endophitic Asia Field None Fungi none NO ITS Grind the tissue Neasy Plant Mini Kit Illumina Sequencing QIIME||| R packages||| Others UNITE database None None None NO the diversity of fungi associated with leaves using both isolation and metabarcoding approaches None 2019 Many of the fungi identified were plant pathogens and may eventually cause diseases in the host OTUs ITS1/ITS4 (White et al., 1990) A highly diverse fungal community associated with leaves of the mangrove plant Acanthus ilicifolius var. xiamenensis revealed by isol.pdf Pang, Ka-Lai; Chi, Wei-Chiung; Chen, Weiling; He, Chih-Chiao; Guo, Sheng-Yu; Cha, Hyo-Jung; Tsang, Ling Ming.; Ho, Tsz Wai.

13632157 https://www.sysrev.com/p/104267/article/13632157 isacadavid9 Woody||| Herbaceous Annual||| Perenne Zea mays, Pinus taeda , Spartina alternifora Leaf Endophitic North America Greenhouse None Bacteria 16S rDNA V5 - V6 NO None Grind the tissue PowerSoil DNA Isolation Kit Illumina Sequencing QIIME Greengenes database None PNA||| blocking oligonucleotide None NO PCR clamps use effect in bacteria reads production None 2020 PCR clamps significantly reducing the proportion of plant reads, yielded 20 times more prokaryotic reads and tripled the number of detected OTUs compared to a commonly used V5-V6 PCR OTUs None A novel PCR-clamping assay reducing plant host DNA.pdf Lef Èvre, Emilie; Gardner, Courtney M.; Gunsch, Claudia K.; Sessitsch, Angela

13632158 https://www.sysrev.com/p/104267/article/13632158 isacadavid9 Herbaceous Perenne Andromeda polifolia, Ledum palustre Leaf Endophitic North America Other None Fungi none NO ITS Grind the tissue PowerPlant Pro DNA isolation kit(MoBio)) Illumina Sequencing USEARCH||| Others NCBI None read filtering None NO culture and non culture isolation methods from Herbarium samples None 2018 through NGS was possible to retrieve a rich community, by culture only one endophyte OTUs ITS1f and ITS4 A novel proof of concept for capturing the diversity of endophytic fungi preserved in herbarium specimens.pdf

13632161 https://www.sysrev.com/p/104267/article/13632161 erika_frydrych Herbaceous Annual Lactuca sativa Leaf Epyphitic Europe Other None Bacteria 16S rDNA V4 NO None Wash the tissue In-house method Roche 454 Sequencing QIIME||| Others Greengenes database None None None NO its not clear if results gained from single samples are representative of the community composition|||intra-sample variation None 2015 γ-Proteobacteria were most abundant OTUs ANALYSIS OF THE BACTERIAL EPIPHYTIC MICROBIOTA OF OAK LEAF LETTUCE WITH 16S RIBOSOMAL RNA GENE ANALYSIS.pdf

13632162 https://www.sysrev.com/p/104267/article/13632162 erika_frydrych Herbaceous Annual (Lactuca sativa Leaf All Asia Field None Bacteria 16S rDNA V5 - V6 NO None Wash the tissue In-house method Illumina Sequencing MOTHUR||| QIIME SILVA database None Microorganism specific primers None YES To investigate the microbiota of lettuce during different seasons Cytoscape 2018 The number of total bacterial was greater in lettuce collected in July than in that collected in April, with reduced diversity; The bacterial compositions varied according to the site and season of sample collection; Potential pathogenic species showed season-specific differences OTUs None Analysis of the Microbiota on Lettuce (Lactuca sativa L.) Cultivated in South Korea to Identify Foodborne Pathogens.pdf Yu, Yeon-Cheol; Yum, Su-Jin; Jeon, Da-Young; Jeong, Hee-Gon

13632163 https://www.sysrev.com/p/104267/article/13632163 erika_frydrych Herbaceous Perenne Vitis vinifera other Epyphitic Europe Field None Bacteria||| Fungi 16S rDNA V1 - V2 NO ITS Grind the tissue PowerSoil DNA Isolation Kit Roche 454 Sequencing Others Others None None None NO different years|||different sampling dates None 2016 Viticultural management system had no significant effect on abundance of fungi or bacteria in both years and at all three sampling dates; No distinct fungal or bacterial communities were associated with the different maturation stages or management systems OTUs ITS1 and ITS2 (Buée M, et al 2009) Are Epiphytic Microbial Communities in the Carposphere of Ripening Grape Clusters (Vitis vinifera L.) Different between Conventional.pdf Kecskeméti, Elizabeth; Berkelmann-Löhnertz, Beate; Reineke, Annette

13632164 https://www.sysrev.com/p/104267/article/13632164 erika_frydrych Herbaceous Annual Lactuca sativa Leaf Endophitic Asia Other None Bacteria 16S rDNA V4 - V5 NO None Wash the tissue FastDNA Spin Kit for Soil Illumina Sequencing QIIME Others None Microorganism specific primers None YES abundance and diversity of ARGs|||the microbial communities in conventionally and organically produced lettuce Cytoscape 2016 134 ARGs were detected in the phyllosphere and leaf endophyte of the samples; Difference in the microbial communities between OPL and CPL, and a lower diversity of both phyllosphere and leaf endophytic bacteria in OPL than in CPL; The profile of ARGs is affected by bacterial community compositions; OTUs Does organically produced lettuce harbor higher abundance of antibiotic resistance genes than conventionally produced.pdf Zhu, Bokai; Chen, Qinglin; Chen, Songcan; Zhu, Yong-Guan

13632165 https://www.sysrev.com/p/104267/article/13632165 erika_frydrych Herbaceous Perenne pueraria montana Leaf Endophitic North America Field None Fungi none NO ITS2 Wash the tissue Others Illumina Sequencing MOTHUR||| VSEARCH||| R packages UNITE database None read filtering None NO impact of multiple environmental parameters on folias endophyte communities None 2020 Communities were diverse and structured by many factors but location, genotype and traffic were the strongest drivers of community composition; Several OTUs were positively and strongly associated with pathogen OTUs nexF-N[3]-fITS7 (Ihrmark, K et al, 2012) and nexR-N[3]-ITS4 (White, et al, 1990) Drivers of Foliar Fungal Endophytic Communities of Kudzu (Pueraria montana var. lobata) in the Southeast United States.pdf

13632166 https://www.sysrev.com/p/104267/article/13632166 erika_frydrych Herbaceous Perenne Origanum vulgar Leaf||| Stem||| Flower Epyphitic||| Endophitic Europe Farm None Bacteria 16S rDNA V1 - V2||| 16S rDNA V3 NO None Wash the tissue Qiagen DNeasy Blood & Tissue Kit||| FastDNA Spin Kit for Soil Illumina Sequencing USEARCH||| QIIME||| Others NCBI||| Others None Microorganism specific primers None NO s (EOs). Combined culture-dependent and -independent approache None 2018 e. Epiphyte bacteria were more abundant than the endophyte one; . The number of presumptive lactic acid bacteria increased throughout oregano life cycle, according to the plant organ; highest epiphyte bacterial diversity at early vegetative and full-flowering; OTUs None Dynamic and Assembly of Epiphyte and Endophyte Lactic Acid Bacteria During the Life Cycle of Origanum vulgare L..pdf Francesca, Nicola; Puglisi, Edoardo; Cagno, Raffaella Di.; Pontonio, Erica; Tarraf, Waed; Filannino, Pasquale; De Mastro, Giuseppe; Gobbetti, Marco

13632167 https://www.sysrev.com/p/104267/article/13632167 erika_frydrych Herbaceous Perenne Halophila stipulacea Leaf|||Root||| Rhizome Epyphitic Asia Field None Bacteria 16S rDNA V4 - V5 NO None Others PowerSoil DNA Isolation Kit Roche 454 Sequencing MOTHUR Ribosomal Database Project None read filtering None NO depict variations in morphology, biochemistry and epiphytic bacterial communities along a depth grad|||different tissues None 2017 A different ecological status of H. stipulacea at the edges of the gradient (4–28 m), where plants showed not only

marked differences in morphology and biochemistry, but also the most distinct associated bacterial consortium; OTUs None Ecophysiological Plasticity and Bacteriome Shift in the Seagrass Halophila stipulacea along a Depth Gradient in the Northern Red Sea.pdf Ainley, Lara Beth.; Winters, Gidon; Rotini, Alice; Mejia, Astrid Y.; Costa, Rodrigo; Migliore, Luciana

13632169 https://www.sysrev.com/p/104267/article/13632169 erika_frydrych Woody Perenne Pinus massoniana Leaf|||Root||| Stem Endophitic Asia Greenhouse None Bacteria 16S rDNA V5 - V7 NO None Wash the tissue FastDNA Spin Kit for Soil Illumina Sequencing MOTHUR||| R packages Ribosomal Database Project None None None NO different disease-resistant species|||different tissues None 2021 There were no obvious differences in the composition of the endophytic bacterial community of different disease-resistant P. massoniana in the leaves, but there were obvious differences in the roots, stems and treetops. OTUs none Endogenous bacterial community structure of Pinus massoniana with differing resistance to pine wilt disease.pdf Lu, Wei; Zhao, Xiao-Jia; Tan, Jia-Jin

13632171 https://www.sysrev.com/p/104267/article/13632171 erika_frydrych Woody Perenne Pyrus ussuriensi Fruit||| Flower||| Leaf||| Stem|||Root Endophitic Asia Field None Bacteria 16S rDNA V3 - V4 NO None Grind the tissue In-house method||| FastDNA Spin Kit for Soil Illumina Sequencing MOTHUR||| R packages SILVA database None None PICRUSt NO y, the structure of endophytic bacterial communities associated with different tissues and soil o None 2018 Samples from three different soils had significant differences in microbial communities structure. Redundancy analysis showed that the bacterial community structure correlated significantly with soil properties. OTUs None Endophytic bacterial communities of Jingbai Pear trees in north China analyzed with Illumina sequencing of 16S rDNA.pdf Ren, Fei; Dong, Wei; Yan, Dong-Hui

13632172 https://www.sysrev.com/p/104267/article/13632172 carolinesalvati Herbaceous Annual Glycine max L. Merrill Leaf Endophitic South America Greenhouse None Bacteria 16S rDNA V3 - V4 NO None Grind the tissue PowerSoil DNA Isolation Kit Illumina Sequencing MOTHUR Ribosomal Database Project None read filtering PICRUSt NO Plant genotype None 2018 The obtainment of the GM event 1Ea2939 showed minimum effects on the microbial community and in the potential for chemical-genetic communication Endophytic bacterial microbiome associated with leaves of genetically modified (AtAREB1) and conventional (BR 16) soybean plants.pdf Montanari-Coelho, Katiúscia Kelli.; Tenório Costa, Alessandra; Polonio, Julio Cesar.; Azevedo, João Lúcio.; Regina, Silvana; Marin, Rockenbach; Fuga-Pagliarini, Renata; Fujita, Yasunari; Yamaguchi-Shinozaki, Kazuko; Nakashima, Kazuo; Pamphile, João Alencar.; Lima, Alexandre

13632174 https://www.sysrev.com/p/104267/article/13632174 erika_frydrych Woody Perenne Fraxinus excelsior Leaf|||Root||| Seed Endophitic Europe Field None Fungi none NO ITS1 Others Others Illumina Sequencing QIIME||| R packages UNITE database None None None NO . None 2021 . OTUs . Assessing Genotypic and Environmental Effects on Endophyte Communities of Fraxinus (Ash) Using Culture Dependent and Independent DNA Sequencing.pdf

13632175 https://www.sysrev.com/p/104267/article/13632175 erika_frydrych Herbaceous Perenne Eighteen rose varieties other Endophitic Asia Field None Bacteria 16S rDNA V3 - V4 NO None Wash the tissue|||Grind the tissue E.Z.N.A. Soil DNA Kit Illumina Sequencing Others||| R packages Others None Others PICRUSt NO 18 roses varities None 2020 Each endophyte sample contained multiple KEGG functional modules related to human metabolism and health OTUs None Assessment of endophytic bacterial diversity in rose by high-throughput sequencing analysis.pdf Xia, Ao-Nan; Liu, Jun; Kang, Da-Cheng; Zhang, Hai-Guang; Zhang, Ru-Hua; Liu, Yun- Guo

13632177 https://www.sysrev.com/p/104267/article/13632177 carolinesalvati Herbaceous Annual Diplotaxis tenuifolia|||Lactuca sativa Leaf Epyphitic Europe Farm None Bacteria 16S rDNA V6-V8||| 16S rDNA V4 NO None Wash the tissue Neasy Plant Mini Kit Illumina Sequencing||| Sanger Sequencing MOTHUR SILVA database||| Ribosomal Database Project None read filtering None NO Season|||Plant species|||geography None 2015 the effect on bacterial colonization of leaves, at least in part must, be sea- sonally driven rather than driven solely by leaf maturity. Bacterial Communities Associated with Surfaces of Leafy Greens Shift in Composition and Decrease in Richness over Time.pdf Merete, Wiken; Dees, Erik; Lysøe, Berit; Nordskog, May Bente.; Brurberg; Dees

13632178 https://www.sysrev.com/p/104267/article/13632178 erika_frydrych Herbaceous Annual Arabidopsis thaliana Root||| Leaf All North America Field None Bacteria 16S rDNA V5 - V7 NO None Grind the tissue PowerSoil DNA Isolation Kit Roche 454 Sequencing MOTHUR||| R packages SILVA database||| Ribosomal Database Project None read filtering None NO different tissues None 2013 In the roots, richness is higher in the epiphytic communities compared to the endophytic compartment while the reverse is true for the leaves; leaf and root endophytic

compartments do not differ in richness, diversity and evenness, but differ in community composition; OTUs none Bacterial Communities Associated with the Leaves and the Roots of Arabidopsis thaliana.pdf Bodenhausen, Natacha; Horton, Matthew W.; Bergelson, Joy

13632179 https://www.sysrev.com/p/104267/article/13632179 erika_frydrych Herbaceous Perenne Solanum lycopersicum L. Leaf||| Fruit|||Root Endophitic Asia Greenhouse None Bacteria 16S rDNA V3 - V4 NO None Grind the tissue||| Wash the tissue FastDNA Spin Kit for Soil Illumina Sequencing MOTHUR Ribosomal Database Project None None None NO bacterial size and community from rhizosphere, phyllosphere and endosphere|||of roots, stems, leaves, fruits and seeds None 2019 The bacterial communities from the soil-type samples (rootzone soil and rhizosphere) showed the highest richness and diversity. The lowest bacterial diversity occurred in the phyllospheric samples, while the lowest richness occurred in the endosphere. OTUs none Bacterial communities in the rhizosphere, phyllosphere and endosphere of tomato plants.pdfDong, Chun-Juan; Wang, Ling-Ling; Li, Qian; Shang, Qing-Mao

13632181 https://www.sysrev.com/p/104267/article/13632181 erika_frydrych Herbaceous Perenne Nepenthes albomarginata|||Nepenthes ampullaria|||Nepenthes mirabilis|||Nepenthes hirsuta Leaf All Asia Field None Bacteria 16S rDNA V5 - V6 NO None Others PowerSoil DNA Isolation Kit Roche 454 Sequencing R packages Greengenes database||| SILVA database||| Ribosomal Database Project None None None NO Comparing pitcher fluids and bacterial community compositionto another pitcher plant, Sarracenia. None 2015 t the bacterial communities of both opened and unopened pitchers were most similar to that of Sarracenia and to that in the phyllosphere OTUs none Bacterial diversity and composition in the fluid of pitcher plants of the genus Nepenthes.pdf Takeuchi, Yayoi; Chaffron, Samuel; Salcher, Michaela M.; Shimizu-Inatsugi, Rie; Kobayashi, Masaki J.; Diway, Bibian; Von Mering, Christian; Pernthaler, Jakob; Shimizu, Kentaro K.

13632182 https://www.sysrev.com/p/104267/article/13632182 erika_frydrych Woody Perenne poplar Bulk soil|||Root||| Stem||| Leaf All Europe Field None Bacteria 16S rDNA V5 - V6 NO None Wash the tissue PowerSoil DNA Isolation Kit||| In-house method Illumina Sequencing Others Others None None None NO . None 2018 . OTUs . Bacterial diversity associated with poplar trees grown on a Hg-contaminated site Community characterization and isolation of Hg-resistant plant growth-promoting bacteria.pdf Durand, Alexis; Maillard, François; Alvarez-Lopez, Vanessa; Guinchard, Sarah; Bertheau, Coralie; Valot, Benoit; Blaudez, Damien; Chalot, Michel

13632185 https://www.sysrev.com/p/104267/article/13632185 carolinesalvati Herbaceous Perenne Glycine max|||Brassica napus|||Phaseolus vulgaris Leaf||| Bulk soil All North America Farm None Bacteria 16S rDNA V5 - V7 NO None Grind the tissue Others|||E.Z.N.A. Soil DNA Kit Illumina Sequencing MOTHUR||| QIIME Ribosomal Database Project||| Greengenes database None None PICRUSt YES Season|||Plant Species Others 2014 The leaf microbiota at the beginning of the season is very strongly influenced by the soil microbiota but, as the season progresses, it becomes significantly less diverse Seasonal Community Succession of the Phyllosphere Microbiome.pdf

13632186 https://www.sysrev.com/p/104267/article/13632186 erika_frydrych Herbaceous Perenne Cymodocea nodosa|||Cymodocea cylindracea Leaf||| Stem Epyphitic Europe Field Biotic Bacteria 16S rDNA V4 NO None Wash the tissue In-house method Illumina Sequencing MOTHUR||| R packages SILVA database None None None NO . None 2021 . OTUs . Seasonal Dynamics of Epiphytic Microbial Communities on Marine Macrophyte Surfaces.pdf Bisbal, Antonio Busquets.; Tomas, Fiona; Korlevi, Marino; Markovski, Marsej; Zhao, Zihao; Herndl, Gerhard J.; Najdek, Mirjana

13632187 https://www.sysrev.com/p/104267/article/13632187 fickgustavo Woody Perenne grapevine Leaf Epyphitic Africa Farm Biotic||| Metals Bacteria||| Fungi 16S rDNA V3 - V4 NO ITS Wash the tissue FastDNA Spin Kit for Soil Illumina Sequencing QIIME||| Others NCBI||| UNITE database||| Greengenes database None PNA None NO microbial change treated with copper sulphate and Lactobacillus plantarum MW-1 None 2020 Only the family of Lactobacillaceae systematically changed between treatments, while the bacterial community remained relatively stable over time. ASVs ITS1 and ITS2 (Del Frari, G et al. 2019) Seasonal epiphytic microbial dynamics on grapevine leaves under biocontrol and copper fungicide treatments.pdf Gobbi, Alex; Kyrkou, Ifigeneia; Filippi, Elisa; Ellegaard-Jensen, Lea; Hansen, Lars Hestbjerg.

13632188 https://www.sysrev.com/p/104267/article/13632188 erika_frydrych Herbaceous Annual Suaeda salsa Seed Endophitic Asia Field None Fungi none NO ITS1||| ITS2 Grind the tissue Neasy Plant Mini Kit Roche 454 Sequencing MOTHUR||| R packages NCBI||| UNITE database None Others None NO seed fungal endophytes present in a coastal plant None 2016 extremely low species richness; OTUs ITS1F/ITS2, fITS7/ITS4 and ITS-1F/ITS4 Seed endophytic microbiota in a coastal plant and phytobeneficial properties of the fungus Cladosporium cladosporioides.pdf Qin, Yuan; Pan, Xueyu; Yuan, Zhilin

13632189 https://www.sysrev.com/p/104267/article/13632189 erika_frydrych Herbaceous Annual maize|||rice|||switchgrass|||Brachiaria decumbens|||wheat|||sugarcane|||barley|||sorghum|||tomato|||coffee|||pea|||Brachypodium distachyon|||common bean|||cassava|||soybean|||sunflower|||Arabidopsis thaliana Seed||| Rhizosphere|||Root||| other Endophitic North America||| South America Field||| Farm None Bacteria||| Fungi 16S rDNA V4 NO ITS1 Others Others Illumina Sequencing USEARCH Ribosomal Database Project None PNA others NO . None 2021 . OTUs . Seed-Transmitted Bacteria and Fungi Dominate Juvenile Plant Microbiomes.pdf Prakash Verma, Jay; Johnston-Monje, David; Gutiérrez, Janneth P.; Becerra Lopez-Lavalle, Luis Augusto.

13632190 https://www.sysrev.com/p/104267/article/13632190 isacadavid9 Woody Perenne Picea abies Leaf All Europe Field None Fungi none Yes Other Grind the tissue In-house method Illumina Sequencing Others Others None read filtering None NO . None 2015 . Genome . Serendipitous Meta-Transcriptomics The Fungal Community of Norway Spruce (Picea abies).pdf

13632191 https://www.sysrev.com/p/104267/article/13632191 isacadavid9 Woody Perenne Mussaenda shikokiana Leaf All Asia Field None Fungi none NO ITS2 Grind the tissue In-house method Illumina Sequencing QIIME||| MOTHUR||| R packages UNITE database None None FUNGuild YES geographical altitude R packages||| Gephi 2018 . OTUs ITS1F (Gardes & Bruns, 1993) and ITS4 (White et al., 1990) Shifts in community composition and co-occurrence patterns of phyllosphere fungi inhabiting Mussaenda shikokiana along an elevation gradient.pdf Zhang, Dianxiang; Qian, Xin; Chen, Liang; Guo, Xiaoming; He, Dan; Shi, Miaomiao

13632193 https://www.sysrev.com/p/104267/article/13632193 isacadavid9 Herbaceous Annual Triticum aestivum L Leaf Epyphitic Asia Greenhouse None Bacteria 16S rDNA V4 - V5 NO ITS Others Others Illumina Sequencing MOTHUR Ribosomal Database Project None None None NO . None 2016 . OTUs ITS1-F1737 and ITS2-R2043 Shifts of microbial communities of wheat (Triticum aestivum L.) cultivation in a closed artificial ecosystem.pdf Qin, Youcai; Fu, Yuming; Dong, Chen; Jia, Nannan; Liu, Hong

13632194 https://www.sysrev.com/p/104267/article/13632194 erika_frydrych Herbaceous Perenne Mussaenda kwangtungensis Rhizosphere|||Root||| Leaf Endophitic Asia Field None Fungi none NO ITS2 Grind the tissue PowerSoil DNA Isolation Kit Illumina Sequencing MOTHUR||| QIIME||| R packages UNITE database||| Ribosomal Database Project None read filtering FUNGuild YES Different tissues Gephi 2019 Rhizosphere soil exhibited the highest level of fungal diversity, whereas the lowest level was found in the leaf endosphere OTUs ITS1F (Gardes and Bruns, 1993), ITS4 (White et al., 1990) and fITS7 (Ihrmark et al., 2012) Leaf and Root Endospheres Harbor Lower Fungal Diversity and Less Complex Fungal Co-occurrence Patterns Than Rhizosphere.pdf Jeewon, Rajesh; Kaushik, Nutan; Alves, Danielle Karla.; Silva, Da; Zheng, Yong; Qian, Xin; Li, Hanzhou; Wang, Yonglong; Wu, Binwei; Wu, Mingsong; Chen, Liang; Li, Xingchun; Zhang, Ying; Wang, Xiangping; Shi, Miaomiao; Guo, Liangdong; Zhang, Dianxiang

13632195 https://www.sysrev.com/p/104267/article/13632195 isacadavid9 Woody Perenne Fagus sylvatica Leaf|||Root||| Bulk soil All Europe Field None Fungi none NO ITS1 Grind the tissue Neasy Plant Mini Kit Roche 454 Sequencing Others NCBI_fungi ITS database None None None NO elevation None 2014 . OTUs ITS1F and reverse ITS2 (Leaf and Root-Associated Fungal Assemblages Do Not Follow Similar Elevational Diversity Patterns.pdf Coince, Aurore; Cordier, Tristan; Lengelle, Juliette; Defossez, Emmanuel; Vacher, Corinne; ´cile Robin, Ce; ˆt Marc ¸ais, Benoı; Bue, Marc

13632196 https://www.sysrev.com/p/104267/article/13632196 erika_frydrych Herbaceous Annual||| Perenne Spinacia oleracea|||Diplotaxis tenuifolia Leaf Endophitic Europe Field Nutrient Bacteria||| Fungi Other NO ITS Grind the tissue Others Illumina Sequencing MOTHUR SILVA database None None None NO Four levels of N fertilizer|||Culture-independentandculture- dependent analyzing techniques None 2019 Plant species, leaf mineral content, and annual variations affects the microbial diversity in the phyllosphere; alpha diversity of bacterial communities decreased in response to increasing nitrogen fertilizer dose, whereas viable counts showed no differences; OTUs ITS7F (Ihrmark et al., 2012) and ITS4R (Whiteetal.,1990) Leaf mineral content govern microbial community structure in the phyllosphere of spinach (Spinacia oleracea) and rocket (Diplotaxis.pdf Darlison, Julia; Mogren, Lars; Rosberg, Anna Karin.; Grudén, Maria; Minet, Antoine; Liné, Clarisse; Mieli, Morgane; Bengtsson, Torbjörn; Håkansson, Åsa; Uhlig, Elisabeth; Becher, Paul G.; Karlsson, Maria; Alsanius, Beatrix W.

13632197 https://www.sysrev.com/p/104267/article/13632197 erika_frydrych Herbaceous Perenne Solanum lycopersicum Leaf Endophitic Asia Field None Bacteria||| Fungi 16S rDNA V4 NO ITS1 Grind the tissue In-house method Illumina Sequencing Others||| R packages Others None PNA None NO eight varieties of grafted tomato plants None 2019

both bacterial and fungal community structures did not significantly differ among tomato plants with different rootstock genotypes. Leaf microbiome structures can be affected/unaffected by rootstock genotypes in grafted crop plants OTUs Leaf-associated microbiomes of grafted tomato plants.pdf Toju, Hirokazu; Okayasu, Koji; Notaguchi, Michitaka

13632199 https://www.sysrev.com/p/104267/article/13632199 isacadavid9 Herbaceous Annual Messerschmidia sibirica Rhizosphere||| Leaf||| Stem|||Root||| Bulk soil Endophitic Asia Field Salt Bacteria 16S rDNA V5 - V7 NO None Grind the tissue PowerSoil DNA Isolation Kit Illumina Sequencing QIIME||| MOTHUR||| R packages SILVA database None Microorganism specific primers None NO . None 2017 . OTUs . llumina-Based Analysis of Endophytic and Rhizosphere Bacterial Diversity of the Coastal Halophyte &ITMesserschmidia sibirica&IT.pdf

13632200 https://www.sysrev.com/p/104267/article/13632200 isacadavid9 Woody Perenne Castanea henryi, Castanopsis sclerophylla, Choerospondias axillaris, Nyssa sinensis, Sapindus saponaria, Triadica sebifera Leaf All Asia Field Biotic Fungi none NO ITS Grind the tissue Others Illumina Sequencing Others||| VSEARCH NCBI None None FUNGuild NO Fungal infestion None 2021 . OTUs TS1F primer and the reverse ITS4 primer Local Tree Diversity Suppresses Foliar Fungal Infestation andDecreases Morphological but Not Molecular Richness in aYoung Subtropical Forest.pdf

13632201 https://www.sysrev.com/p/104267/article/13632201 isacadavid9 Herbaceous Annual Galium album Leaf All Europe Field heat Bacteria 16S rDNA V5 - V6 NO None Grind the tissue Others Illumina Sequencing Others SILVA database None Microorganism specific primers None NO . None 2018 . OTUs . Long-Term Warming Shifts the Composition of Bacterial Communities in the Phyllosphere of Galium album in a Permanent Grassland Field-Experiment.pdf Arora, Naveen Kumar.; Campisano, Andrea; Glaeser, Stefanie P.; Aydogan, Ebru L.; Moser, Gerald; Müller, Christoph; Kämpfer, Peter

13632202 https://www.sysrev.com/p/104267/article/13632202 erika_frydrych Woody Perenne Pinus sylvestris other Endophitic Europe Field None Fungi none NO ITS2 Others In-house method PacBio sequencing Others Ribosomal Database Project None None others NO . None 2020 . OTUs||| ASVs||| Genome . Managed and Unmanaged Pinus sylvestris Forest Stands Harbour Similar Diversity and Composition of the Phyllosphere and Soil Fungi.pdf Ūratė Lynikienė, J; Marčiulynienė, Diana; Marčiulynas, Adas; Ūras Gedminas, Art; Vaičiukynė, Miglė; Menkis, Audrius

13632203 https://www.sysrev.com/p/104267/article/13632203 erika_frydrych Woody Annual Quercus macrocarpa Leaf All North America Field None Fungi none NO ITS1 Grind the tissue UltraClean Soil DNA Isolation Kit Roche 454 Sequencing Others Others None Microorganism specific primers None NO fungal richness, diversity and community composition|||among trees located within and outside a small urban center None 2009 The richness of the hyperdiverse phyllosphere communities could not be reliably estimated; as nearly one-half of the molecular OTUs were singletons. The fungal communities within and outside the urban center differed in richness and diversity, which were lower within the urban development. OTUs (ITS1F; Gardes & Bruns, 1993) or universal (ITS2; White et al., 1990 Massively parallel 454 sequencing indicates hyperdiverse fungal communities in temperate Quercus macrocarpa phyllosphere.pdf Jumpponen, A; Jones, K L.

13632204 https://www.sysrev.com/p/104267/article/13632204 erika_frydrych Herbaceous Annual Hydrilla verticillata Leaf Epyphitic Asia Field None Bacteria 16S rDNA V3 - V4 NO None Wash the tissue PowerSoil DNA Isolation Kit||| Power Biofilm DNA isolation kit Illumina Sequencing R packages Others None read filtering None YES Increase of H 2 O 2 and malondialdehyde (MDA) content and decrease of soluble proteins concentration Gephi 2020 Bacterial community in deciduous layer sediments was more suscep- tible than in epiphytic biofilms during decay process. OTUs None Bacterial succession in epiphytic biofilms and deciduous layer sediments during Hydrilla verticillata decay A field investigation.pdf Si, Tingting; Chen, Hezhou; Qiu, Zheng; Zhang, Lisha; Ohore, Ebiotubo; Zhang, Songhe

13632205 https://www.sysrev.com/p/104267/article/13632205 erika_frydrych Woody Annual Vitis vinifera Leaf||| Flower|||Root Endophitic Europe Other None Fungi none NO ITS1 Others In-house method Illumina Sequencing QIIME UNITE database None Others FUNGuild NO . None 2018 . OTUs . Biodiversity of fungi on Vitis vinifera L. revealed by traditional and high-resolution culture-independent approaches.pdf Jayawardena, Ruvishika S.; Purahong, Witoon; Zhang, Wei; Wubet, Tesfaye; Li, Xinghong; Liu, Mei; Zhao, Wensheng; Hyde, Kevin D.; Liu, Jianhua; Yan, Jiye; Luteobubalina, A; Physalacriaceae, Kile; Root, P; Terreus Thom, A; Frisvad, Samson &.; De Bary, G; Arnaud; Zebrina, C; Mycosphaerellaceae, Pass *.; Ex, Kunze; Braun, U; Crous; Mujica, Chile; Chrysosporium,); Ulfig,

Guarro; Onygenaceae; Viticola Prunet, Cladochytrium; Bensch, Crous; Asperulatum Bensch, C; Vries; Limoniforme Bensch, C; Oxysporum, C; Curtis, M A.; Ramotenellum, C; Schub, K; Braun; Cladosporiaceae; Rectoides Bensch, C; Shin, H D.; Tenellum, C; Berlandieri Viala, C; Gardner, D E.; Phillips, A J L.; Alves, A; Vilgalys, Desm).; Johnson, Jacq; Wei, C T.

13632206 https://www.sysrev.com/p/104267/article/13632206 isacadavid9 Woody Perenne Magnolia grandiflora Leaf Epyphitic North America Field None Bacteria 16S rDNA V4 NO None Wash the tissue PowerSoil DNA Isolation Kit Illumina Sequencing MOTHUR Others None read filtering None NO . None 2016 . OTUs . Biogeographic Patterns Between Bacterial Phyllosphere Communities of the Southern Magnolia (Magnolia grandiflora) in a Small Forest.pdf Stone, Bram W G.; Jackson, Colin R.

13632209 https://www.sysrev.com/p/104267/article/13632209 erika_frydrych Herbaceous Annual Bromus tectorum Root||| Leaf Endophitic North America Field None Fungi none NO ITS2 Wash the tissue Others Illumina Sequencing QIIME||| USEARCH||| R packages UNITE database||| Ribosomal Database Project None None None NO Differente tissue None 2019 In leaves, the communities assembling from the various inoculum sources were not significantly different from each other and, in roots, they were only slightly different from each other; OTUs ITS4 Fun and 5.8S Fun primers (Taylor et al. 2016) Biotic fltering of endophytic fungal communities in Bromus tectorum.pdf Ricks, Kevin D.; Koide, Roger T.

13632210 https://www.sysrev.com/p/104267/article/13632210 erika_frydrych Herbaceous Perenne Sorghastrum nutans Leaf Endophitic North America Field None Bacteria 16S rDNA V5 - V6 NO None Grind the tissue Neasy Plant Mini Kit Illumina Sequencing USEARCH||| QIIME Greengenes database None blocking oligonucleotide None NO A blocking primer set was developed to reduce amplification of non-target plant DNA. None 2015 DNA can confound molecular studies of bacterial endophytes; It is presented blocking primers that greatly increased efficiency of Illumina-based bacterial amplification; OTUs none Blocking primers reduce co-amplification of plant DNA when studying bacterial endophyte communities..pdf Arenz, Brett E.; Schlatter, Dan C.; Bradeen, James M.; Kinkel, Linda L.

13632211 https://www.sysrev.com/p/104267/article/13632211 isacadavid9 Woody Perenne Pinus sylvestris Leaf All Europe Field Biotic Fungi none NO ITS Grind the tissue In-house method Roche 454 Sequencing Others NCBI_fungi ITS database None None None NO geographic location, tissue health, tree age, stand establishment None 2016 fungal community composition changes both along geographical location as well as with needle health status and needle age OTUs Changes in fungal community of Scots pine (Pinus sylvestris) needles along a latitudinal gradient in Sweden.pdf Millberg, Hanna; Boberg, Johanna; Stenlid, Jan; Dighton, John

13632212 https://www.sysrev.com/p/104267/article/13632212 carolinesalvati Herbaceous Annual Spinacia oleracea Leaf Epyphitic North America Farm Cold Bacteria 16S rDNA V4 NO None Wash the tissue Others Roche 454 Sequencing Others Ribosomal Database Project None None None NO Temperature|||Incubation time None 2011 Growth inhibition of the generaEscherichiawas achieved at 4°C but not at 10°C storage, thus highlighting the importance of temperature in fresh pack- aged spinach. Changes in spinach phylloepiphytic bacteria communities following minimal processing and refrigerated storage described using pyroseq.pdf Lopez-Velasco, G; Welbaum, G E.; Boyer, R R.; Mane, S P.; Ponder, Monica A.

13632213 https://www.sysrev.com/p/104267/article/13632213 carolinesalvati Herbaceous Perenne Glehnia littoralis Root||| Leaf||| Stem Endophitic Asia Field None Bacteria 16S rDNA V3 - V4 NO None Others Others Illumina Sequencing QIIME||| Others SILVA database None None None NO Plant Tissue None 2020 Bacterial communities of different tissues are unique, which could facilitate understanding the diversity of endophytic bacteria in G. littoralis Characteristics and Diversity of Endophytic Bacteria in Endangered Chinese Herb Glehnia littoralis Based on Illumina Sequencing.pdf

13632214 https://www.sysrev.com/p/104267/article/13632214 isacadavid9 Woody Perenne Populus balsamifera, Quercus spp., Fagus sylvatica, Metrosideros polymorpha Leaf All None Field None Fungi none NO ITS1 Others Others Roche 454 Sequencing Others||| R packages NCBI None None None NO . None 2013 . OTUs ITS1-F and ITS4, ITS1-F and ITS2, Meta-analysis of deep-sequenced fungal communities indicates limited taxon sharing.pdf Meiser, Anjuli; Schmitt, Imke

13632215 https://www.sysrev.com/p/104267/article/13632215 fickgustavo Woody Perenne Olea europaea Leaf||| Fruit||| Flower All Europe Farm None Fungi none NO ITS2 Grind the tissue Others Roche 454 Sequencing QIIME UNITE database None read filtering None NO OTUs that may represent pathogenic or non-pathogenic None 2015 the existence of a complex fungal consortium including both phytopathogenic and potentially antagonistic microorganisms that can have a significant impact on olive productions. OTUs universal fungal primers ITS3-ITS4

Metabarcoding Analysis of Fungal Diversity in the Phyllosphere and Carposphere of Olive (Olea europaea).pdf Abdelfattah, Ahmed; Li, Maria Giulia.; Nicosia, Destri; Cacciola, Santa Olga.; Droby, Samir; Schena, Leonardo

13632216 https://www.sysrev.com/p/104267/article/13632216 isacadavid9 Herbaceous Annual Zea mays Stem||| Leaf||| Seed||| Flower Endophitic Africa Farm None Bacteria 16S rDNA V5 - V6 NO None Grind the tissue Others Illumina Sequencing QIIME SILVA database None None None NO Bt and non-Bt maize, developmental stages None 2017 . OTUs . Metagenomic analyses of bacterial endophytes associated with the phyllosphere of a Bt maize cultivar and its isogenic parental line from South Africa.pdf

13632217 https://www.sysrev.com/p/104267/article/13632217 isacadavid9 Woody Perenne paulownia elongata x paulownia fortunei Leaf|||Root||| Rhizosphere Endophitic Europe Field None Bacteria||| Fungi 16S rDNA V3 - V4 NO ITS1 Grind the tissue FastDNA Spin Kit for Soil Illumina Sequencing R packages Ribosomal Database Project None None None NO . None 2019 . ASVs . Metagenomic Analysis of Bacterial and Fungal Community Composition Associated with Paulownia elongata x Paulownia fortunei.pdf

13632218 https://www.sysrev.com/p/104267/article/13632218 isacadavid9 Herbaceous Annual Brassica rapa Leaf All Asia Farm None Bacteria 16S rDNA V5 - V6 NO None Grind the tissue In-house method Illumina Sequencing QIIME Ribosomal Database Project None Microorganism specific primers None NO . None 2018 . OTUs . Metagenomic Approach to Identifying Foodborne Pathogens on Chinese Cabbage.pdf

13632219 https://www.sysrev.com/p/104267/article/13632219 carolinesalvati Herbaceous Perenne Aloe vera Root||| Leaf||| Stem Endophitic Asia Greenhouse None Bacteria 16S rDNA V3 - V4 NO None Wash the tissue Others Illumina Sequencing MOTHUR SILVA database None None None NO Plant Tissue None 2015 NGS has captured effectively the microbiota in aloe vera tissues, improving our understanding of the microbial-plant interactions Metagenomics study of endophytic bacteria in Aloe vera using next-generation technology.pdf Akinsanya, Mushafau Adewale.; Goh, Joo Kheng.; Siew, Ping; Lim; Su, Adeline; Ting, Yien

13632220 https://www.sysrev.com/p/104267/article/13632220 erika_frydrych Woody Perenne Picea abies Root||| other||| Bulk soil Endophitic Europe Field None Bacteria||| Fungi 16S rDNA V4 NO ITS1 Wash the tissue In-house method Illumina Sequencing QIIME||| VSEARCH||| R packages Ribosomal Database Project||| SILVA database None PNA None NO sensitivity of microbial community composition to the growing season and nutrient additions.|||changes in the microbial community after application of a complete mineral nutrient mixture None 2018 phyllosphere microbiota was compositionally distinct from the belowground communities and phyllosphere diversity increased significantly over the growing season but was not influenced by the improved nutrient status of the trees; OTUs s ITS1-F/ITS2 (White et al., 1990) Microbial community response to growing season and plant nutrient optimisation in a boreal Norway spruce forest.pdf

13632221 https://www.sysrev.com/p/104267/article/13632221 erika_frydrych Woody Annual Paulownia spp Leaf Endophitic Europe Field None Bacteria 16S rDNA V3 - V4 NO None Grind the tissue FastDNA Spin Kit for Soil Illumina Sequencing Others Greengenes database None Microorganism specific primers None NO two different plantations|||structural and functional diversity None 2018 microbial populations had an intensive and high metabolic potential; comparisons of carbon source utilization and the diversity showed differences in the microbial comunity of composition OTUs Microbial diversity of Paulownia spp..pdf

13632222 https://www.sysrev.com/p/104267/article/13632222 isacadavid9 Herbaceous Perenne Lactuca sativa Leaf|||Root Epyphitic North America Greenhouse Other Bacteria, Fungi 16S rDNA V4 NO ITS Wash the tissue Others Illumina Sequencing Others UNITE database||| Greengenes database None None None NO . None 2020 . OTUs . Microbiological and Nutritional Analysis of Lettuce Crops Grown on the International Space Station.pdf Gioia, Francesco Di.; Chitarra, Walter; Massa, Gioia D.; Khodadad, Christina L M.; Hummerick, Mary E.; Spencer, Lashelle E.; Dixit, Anirudha R.; Richards, Jeffrey T.; Romeyn, Matthew W.; Smith, Trent M.; Wheeler, Raymond M.

13632223 https://www.sysrev.com/p/104267/article/13632223 isacadavid9 Herbaceous Perenne Osmanthus fragrans Leaf Epyphitic Asia Field Salt Bacteria Other NO None Grind the tissue Others Illumina Sequencing QIIME SILVA database None None others NO . None 2017 . OTUs . Modified RNA-seq method for microbial community and diversity analysis using rRNA in different types of environmental samples.pdf Yan, Yong-Wei; Zou, Bin; Zhu, Ting; Hozzein, Wael N.; Quan, Zhe-Xue

13632224 https://www.sysrev.com/p/104267/article/13632224 erika_frydrych Woody Annual Cassiope tetragona|||Saxifraga cespitosa|||Saxifraga oppositifolia|||Silene acaulis Leaf||| Stem Endophitic None Field None Fungi none NO ITS Wash the tissue PowerMax Soil DNA Isolation Kit Roche 454 Sequencing QIIME Others None read filtering None YES four vascular plant species in the High Arctic Others 2015 Both the climate and host-related fac- tors might shape the fungal communities associated with the four Arctic plant species in this region. OTUs ITS1F and ITS4 (White TJ et al 1990) Endophytic Fungal Communities Associated with Vascular Plants in the High Arctic Zone Are Highly Diverse and Host-Plant Specific.pdf Zhang, Tao; Yao, Yi-Feng

13632225 https://www.sysrev.com/p/104267/article/13632225 erika_frydrych Woody Perenne Vitis vinifera|||Vitis amurensis Leaf Endophitic Asia Other None Fungi none NO ITS Grind the tissue Neasy Plant Mini Kit Illumina Sequencing QIIME||| USEARCH||| Others NCBI||| UNITE database None Microorganism specific primers None NO two genotypes|||young x mature None 2020 Young leaves have significantly higher diversity and richness than that in mature leaves in both cultivars. Endophytic fungal diversity was greater in wild grapevines than in cultivated grapevines in both young and mature leaves. OTUs Endophytic fungal community in grape is correlated to foliar age and domestication.pdf Fan, Yanchun; Gao, Linlin; Chang, Pingping; Li, Zhi

13632229 https://www.sysrev.com/p/104267/article/13632229 isacadavid9 Woody Perenne Populus trichocarpa×Populus maximowiczii Root||| Leaf||| Bulk soil||| Stem||| Branches Epyphitic||| Endophitic Europe Field Metals Fungi none NO ITS Grind the tissue PowerSoil DNA Isolation Kit||| In-house method Illumina Sequencing MOTHUR||| R packages UNITE database None None None YES Tissue Cytoscape 2017 The highest diversity estimated by the Shannon index was found for soil communities, which was negatively affected by soil Hg con- centration OTUs ITS1f and ITS2 Environmental Metabarcoding Reveals Contrasting Belowground and Aboveground Fungal Communities from Poplar at a Hg Phytomanagement Site.pdf Durand, Alexis; Maillard, François; Foulon, Julie; Gweon, Hyun S.; Benoit Valot, &; Chalot, Michel

13632230 https://www.sysrev.com/p/104267/article/13632230 erika_frydrych Herbaceous Perenne Solanum lycopersicum Leaf|||Root||| Fruit||| Rhizosphere||| Stem||| other Epyphitic||| Endophitic Asia Greenhouse None Fungi none NO 18S rRNA Grind the tissue FastDNA Spin Kit for Soil Illumina Sequencing MOTHUR||| Others SILVA database None read filtering None NO Different tissues None 2021 Soil communities were the richest and most abundant, while the endophytes showed the lowest richness and diversity. Epiphytes showed similar distribution patterns in the stems and leaves, but among the endophytes, distinct fungal orders were enriched in different tissues. OTUs Epiphytic and Endophytic Fungal Communities of Tomato Plants.pdf Dong, Chunjuan; Wang, Lingling; Li, Qian; Shang, Qingmao

13632232 https://www.sysrev.com/p/104267/article/13632232 erika_frydrych Woody Perenne Euonymus japonicus Leaf Epyphitic Asia Field Biotic Bacteria||| Fungi 16S rDNA V5 - V7 NO ITS Grind the tissue FastDNA Spin Kit for Soil Illumina Sequencing MOTHUR||| USEARCH SILVA database||| UNITE database None Others None YES diseased (DL) and apparently healthy leaves (HL) Cytoscape 2019 HL harbored greater bacterial and fungal diversity than DL; bacterial and fungal communities in DL exhibited different structures from those in HL; the relative abundance of several bacterial phyla and fungal phyla were altered by powdery mildew; OTUs gITS7 and ITS4 (Ihrmark et al. 2012). Euonymus japonicus phyllosphere microbiome is significantly changed.pdf Zhang, Zhuo; Kong, Xiao; Jin, Decai; Yu, Hao; Zhu, Xun; Su, Xiaofeng; Wang, Pei; Zhang, Ruiyong; Jia, Minghong; Deng, Ye

13632234 https://www.sysrev.com/p/104267/article/13632234 carolinesalvati Woody Perenne Fagus sylvatica Leaf||| Stem||| other All Europe Field None Fungi none NO ITS1 Grind the tissue In-house method Roche 454 Sequencing QIIME NCBI_fungi ITS database None None None NO Plant Tissue|||Geography|||Plant Height None 2012 Within a single tree canopy, dissimilarity between fungal assemblages generally increased with distance between leaves. At the stand scale, dissimilarity between fungal assemblages was correlated with the genetic distance rather than the geographic distance between trees. Spatial variability of phyllosphere fungal assemblages genetic distance predominates over geographic distance in a European beech stand (Fagus syluatica).pdf Cordier, Tristan; Robin, Ecile; Capdevielle, Xavier; Desprez-Loustau, Marie-Laure; Vacher, Corinne; Griffith, Gareth W.

13632235 https://www.sysrev.com/p/104267/article/13632235 isacadavid9 Herbaceous Annual Cannabis sativa Rhizosphere|||Root||| Leaf||| Flower Endophitic North America Greenhouse None Bacteria||| Fungi 16S rDNA V4 NO ITS Grind the tissue Neasy Plant Mini Kit Illumina Sequencing QIIME SILVA database||| UNITE database None None PICRUSt NO Plant cultivars, growth stage None 2020 bes spatio- temporal and cultivar-dependent variations in the fungal and bacterial microbiome ofC. sativa, and details strong cultivar-dependent variance in the belowground microb ASVs ir ITS1F/ITS2 (Gardes and Bruns, 1993; Caporaso et al., 201 Spatio-Temporal and Cultivar-Dependent Variations in the Cannabis Microbiome.pdf Labbé, Jessy L.; Filion, Martin; Comeau, Dominique; Novinscak, Amy; Joly, David L.

13632236 https://www.sysrev.com/p/104267/article/13632236 carolinesalvati Herbaceous Annual Triticum aestivum Leaf All Europe Farm None Fungi none NO ITS1 Grind the tissue Others Roche 454 Sequencing QIIME UNITE database||| NCBI_fungi ITS database None None None NO Geography|||Plant Genotype|||Growth stage|||Plant height|||Disease None 2017 . Analysis of read abundances showed that geographical location had a major effect in shaping the mycobiome in the total dataset, but also leaf position, growth stage and cultivar were important drivers of fungal community Spatiotemporal Variation and Networks in the Mycobiome of the Wheat Canopy.pdf Bakkeren, Guus; Nicolaisen, Mogens; Sapkota, Rumakanta; Jørgensen, Lise N.

13632237 https://www.sysrev.com/p/104267/article/13632237 erika_frydrych Woody Perenne Quercus macrocarpa Leaf Endophitic North America Field None Fungi none NO ITS Others Others Roche 454 Sequencing R packages Others None None None NO . None 2011 . OTUs . Species abundance distributions and richness estimations in fungal metagenomics - lessons learned from community ecology.pdf S E H E R, R; I J U M P P O N E N, A R.; R T I N S C H N I T T L E R, M A.; Unterseher, Martin

13632240 https://www.sysrev.com/p/104267/article/13632240 erika_frydrych Herbaceous Annual Oryza sativa Root||| Leaf Endophitic South America Greenhouse Drought Bacteria functional genes NO None Grind the tissue In-house method Roche 454 Sequencing USEARCH||| QIIME Ribosomal Database Project||| NCBI None Others None NO The flooding effect on the abundance and diverstity of diazotrophic communities on rice plants None 2015 nifH gene was significantly more abundant in roots after flooding, whereas the nifH gene copy numbers in leaves were unaffected and remained low; t root communities of replicate plots were more similar and diverse after flooding than before flooding; OTUs none Strong shift in the diazotrophic endophytic bacterial community inhabiting rice (Oryza sativa) plants after flooding.pdf Ferrando, Lucía; Fern Ández Scavino, Ana; Átedra De Microbiología, C; Sessitsch, Angela

13632241 https://www.sysrev.com/p/104267/article/13632241 isacadavid9 Woody Perenne Phyllostachys edulis Root||| Rhizome||| Rhizosphere||| Leaf Endophitic Asia Field None Bacteria 16S rDNA V5 - V7 NO None Grind the tissue Others Illumina Sequencing MOTHUR Others None None None NO Plant tissue of flowering and non flowering plants None 2021 root and rhizosphere soil revealed different structures of microbial communities between bamboo that is flowering and not flowering, core microorganisms, Flavobacterium, Bacillus and Stenotrophomonas played an important role in the absorption of N elements, which may affect the flowering time OTUs None Structural variability and differentiation of niches in the rhizosphere and endosphere bacterial microbiome of moso bamboo (Phyllostachys edulis).pdf Yuan, Zong-Sheng; Liu, Fang; Liu, Zhen-Yu; Huang, Qiu-Liang; Zhang, Guo-Fang; Pan, Hui

13632242 https://www.sysrev.com/p/104267/article/13632242 carolinesalvati Woody Perenne Shorea leprosula Leaf Endophitic Asia Field None Fungi none NO ITS1 Grind the tissue In-house method Ion Torrent Sequencing Others NCBI_fungi ITS database None None None NO geography None 2016 These findings indicate that highly diverse fungal OTUs form spatially structured communities even within a tropical plantation stand of single tree species. Structure of phyllosphere fungal communities in a tropical dipterocarp plantation A massively parallel next-generation sequencing analysis.pdf Izuno, Ayako; Tanabe, Akifumi S.; Toju, Hirokazu; Yamasaki, Michimasa; Indrioko, Sapto; Isagi, Yuji

13632243 https://www.sysrev.com/p/104267/article/13632243 carolinesalvati Herbaceous Annual Arabidopsis thaliana Leaf All Europe Greenhouse Biotic Bacteria 16S rDNA V5 - V7 NO None Grind the tissue FastDNA Spin Kit for Soil Illumina Sequencing USEARCH||| Others Others None None None NO synthetic community None 2019 m, Rhodococcus) have the greatest potential to affect community structure as keystone species. Synthetic microbiota reveal priority effects and keystone strains in the arabidopsis phyllosphere.pdf

13632245 https://www.sysrev.com/p/104267/article/13632245 isacadavid9 Woody Perenne Vitis vinifera Fruit Epyphitic Africa Field None Fungi none NO ITS1 Wash the tissue Zymobiomics DNA kit Illumina Sequencing Others NCBI None None None NO sites, phenological stages, vine positions within a vineyard None 2017 Fungal population diversity varies with different phenological table grape growth stages and is further influenced by site and vine position within a specific vineyard OTUs ITS1 and ITS2 (White et al.1990) Characterization of fungal communities of developmental stages in table.pdf Carmichael, P C.; Siyoum, N; Chidamba, L; Korsten, Lise

13632246 https://www.sysrev.com/p/104267/article/13632246 isacadavid9 Herbaceous Perenne Ocimum basilicum Leaf Epyphitic Europe Farm None Bacteria 16S rDNA V1 - V3 NO None Wash the tissue In-house method||| Others Roche 454 Sequencing R packages||| Others Ribosomal Database Project None None None NO Sample preparation None 2015 Washing samples produces lower number of bacteria than maceration OTUs None Characterization of the Bacterial Community Naturally Present on Commercially Grown Basil Leaves Evaluation of Sample Preparation Prior to Culture-Independent Techniques.pdf

13632247 https://www.sysrev.com/p/104267/article/13632247 isacadavid9 Herbaceous Annual Oryza sativa Leaf All Asia Field None Bacteria||| Archea Other Yes None Grind the tissue In-house method Illumina Sequencing Kraken||| Bracken NCBI None read filtering iVikodak YES Plant genotype, environment, SNPs Gephi||| R packages 2020 the structure of the rice leaf microbiome is modulated by multiple interactions among host, microbes, and environment Genome None Characterization of the Leaf Microbiome from Whole-Genome Sequencing Data of the 3000 Rice Genomes Project.pdf Roman-Reyna, Veronica; Pinili, Dale; Borja, Frances N.; Quibod, Ian L.; Groen, Simon C.; Alexandrov, Nickolai; Mauleon, Ramil; Oliva, Ricardo

13632248 https://www.sysrev.com/p/104267/article/13632248 isacadavid9 Woody Perenne Vitis vinifera Trunk All Europe Field Biotic Fungi none NO ITS1 Grind the tissue FastDNA Spin Kit for Soil Illumina Sequencing QIIME UNITE database None None None NO tissue location, syntomatic and non-syntomatic tissues Others 2019 Differences in diversity and unique presence of certain fungi in specific areas of the plants were found, g that leaf symptoms are not directly linked with the fungal communities in the w ASVs ITS1F2 – ITS2 (Gaylarde et al., 2017) Characterization of the Wood Mycobiome of Vitis vinifera in a Vineyard Affected by Esca. Spatial Distribution of Fungal Communities a.pdf Carlucci, Antonia; Mugnai, Laura; Del, Giovanni; Frari, Giovanni Del.; Gobbi, Alex; Aggerbeck, Marie Rønne.; Oliveira, Helena; Hansen, Lars Hestbjerg.; Ferreira, Ricardo Boavida.

13632249 https://www.sysrev.com/p/104267/article/13632249 erika_frydrych Woody Perenne Castanea mollissima Leaf||| other Endophitic Asia Farm None Bacteria||| Fungi 16S rDNA V3 - V4 NO ITS2 Grind the tissue In-house method Illumina Sequencing Others||| R packages UNITE database||| SILVA database None None None NO symptomatic and asymptomatic chestnut trees None 2020 The asymptomatic samples had a higher fungal and bacterial diversity than symptomatic ones. OTUs Chinese chestnut yellow crinkle disease influence microbiota composition of chestnut trees.pdf Ren, Fei; Dong, Wei; Shi, Shengqing; Dou, Guiming; Yan, Dong-Hui

13632250 https://www.sysrev.com/p/104267/article/13632250 isacadavid9 Herbaceous Annual Coriandrum sativum Leaf Epyphitic North America Garden Biotic Bacteria 16S rDNA V1 - V3NO None Wash the tissue Others Roche 454 Sequencing|||Illumina Sequencing QIIME Ribosomal Database Project None read filtering None NO Before and after salmonella culture aplication None 2015 Time zero uncultured samples had an abundance of Proteobacteria while the 24-hour enriched samples were composed mostly of Gram-positive Firmicutes OTUs None Cilantro microbiome before and after nonselective pre-enrichment for Salmonella using 16S rRNA and metagenomic sequencing.pdf Jarvis, Karen G.; White, James R.; Grim, Christopher J.; Ewing, Laura; Ottesen, Andrea R.; Beaubrun, Jean-Gilles; Pettengill, James B.; Brown, Eric; Hanes, Darcy E.

13632251 https://www.sysrev.com/p/104267/article/13632251 carolinesalvati Woody Perenne 79 especies Leaf Endophitic Central America Field None Fungi none NO ITS Grind the tissue Others Illumina Sequencing USEARCH||| Others UNITE database None None None NO Climate|||Season None 2021 endophyte richness varies negatively with temperature seasonality. Endophyte community structure and taxonomic composition reflect both temperature sea- sonality and climate Climate and seasonality drive the richness and composition of tropical fungal endophytes at a landscape scale.pdf Oita, Shuzo; Ibáñez, Alicia; Lutzoni, François; Miadlikowska, Jolanta; Geml, József; Lewis, Louise A.; Hom, Erik F Y.; Carbone, Ignazio; U'ren, Jana M.; Arnold, & A Elizabeth.

13632252 https://www.sysrev.com/p/104267/article/13632252 carolinesalvati Herbaceous Perenne Atractylodes lancea Root||| Leaf Endophitic Asia Greenhouse None Bacteria 16S rDNA V3 - V4 NO None Grind the tissue FastDNA Spin Kit for Soil Illumina Sequencing Others Ribosomal Database Project None read filtering None NO Plant tissue None 2018 First glimpse of the inhabiting bacterial microbiota that transmit between inside of plant leaf and root compartments. Colonization cues of leaf- and root-inhabiting bacterial microbiota of Atractylodes lancea derived in vitro and in vivo.pdf Jia, Yong; Fu, Wanqiu; Xu, Man; Zhou, Jiayu; Xu, Risheng; Dai, Chuanchao

13632254 https://www.sysrev.com/p/104267/article/13632254 carolinesalvati Woody||| Herbaceous Annual||| Perenne 133 species Root||| Leaf Endophitic Asia Field None Bacteria||| Fungi 16S rDNA V4 NO ITS1 Grind the tissue In-house method Illumina Sequencing Others Others None PNA None NO Plant species|||Season|||Nativeness|||Stem type|||Mycorrhizal type of host plants None 2019 Some prokaryotes and Fungi have host preference and are involved in survival, growth, or environmental resistance of host plants. Factors Influencing Leaf- and Root-Associated Communities of Bacteria and Fungi Across 33 Plant Orders in a Grassland.pdf Timm, Collin M.; Toju, Hirokazu; Kurokawa, Hiroko; Kenta, Tanaka

13632255 https://www.sysrev.com/p/104267/article/13632255 isacadavid9 Herbaceous Perenne Saccharum spp. Leaf||| Bulk soil|||Root||| Stem All Oceania Field None Bacteria||| Fungi 16S rDNA V3 - V4 NO ITS2 Grind the tissue PowerPlant Pro DNA isolation kit(MoBio)) Illumina Sequencing MOTHUR Greengenes database||| UNITE database None PNA||| read filtering None YES plant compartment, growing region, crop age, variety, Yellow Canopy Syndrome Cytoscape 2018 We detected a coreset of microbiota and identified members of the

coremicrobiome that were influenced by YCS incidence. OTUs FITS7-ITS4R, Ihrmarket al., 2012 Field study reveals core plant microbiota and relative importance of their drivers.pdf Hamonts, Kelly; Trivedi, Pankaj; Garg, Anshu; Janitz, Caroline; Grinyer, Jasmine; Holford, Paul; Botha, Frederik C.; Anderson, Ian C.; Singh, Brajesh K.

13632256 https://www.sysrev.com/p/104267/article/13632256 erika_frydrych Woody Annual Quercus robur|||Carpinus betulus|||Castanea sativa|||Vitis vinifera Leaf Endophitic Europe Field None Fungi none NO ITS2 Grind the tissue Others Illumina Sequencing VSEARCH||| USEARCH||| R packages UNITE database None read filtering None NO short-distance dispersal events between vineyard and forest patches shape foliar fungal communities.|||four locations None 2016 foliar fungal communities in vineyards and forest patches differentiate over the growing season; the richness of foliar fungal communities in grapevine decreased; The composition of airborne communities did not differ between habitats. OTUs fITS7 and ITS4 (Ihrmark et al., 2012) Foliar fungal communities strongly differ between habitat patches in a landscape mosaic.pdf Fort, Thomas; ´cile Robin, Ce; Capdevielle, Xavier; Delie, Laurent; Vacher, Corinne

13632257 https://www.sysrev.com/p/104267/article/13632257 isacadavid9 Herbaceous Annual Zea mays Leaf Epyphitic Europe||| North America Field Drought Bacterial, Fungi functional genes Yes Functional genes Wash the tissue PowerSoil DNA Isolation Kit Illumina Sequencing Others MetaPhlAn2 None read filtering HUMAnN2 NO Drought and normal treated plants None 2022 Maizephyllospherefunctionalprofilesrevealedawide varietyofmetabolicandregulatoryprocessesthatdifferedindroughtandnormalwaterconditions Genome Functional gene categories differentiate maize leaf drought-related microbial epiphytic communities.pdf Methe, Barbara A.; Hiltbrand, David; Roach, Jeffrey; Xu, Wenwei; Gordon, Stuart G.; Goodner, Brad W.; Stapleton, Ann E.

13632260 https://www.sysrev.com/p/104267/article/13632260 erika_frydrych Woody Perenne Quercus petraea Leaf Endophitic Europe Field None Fungi none NO ITS1 Grind the tissue PowerSoil DNA Isolation Kit Roche 454 Sequencing Others Others None Microorganism specific primers None NO development of the fungal community over 24 months of litter decomposition|||differente leaves stages|||activity of cellulolytic enzymes None 2012 Fungal diversity was lowest at leaf senescence, Basidiomycota phylum increased with time, Some fungi associated with living tree leaves are also found in association with decomposing leaf litter; litter decomposition is mediated by various fungal taxa OTUs Fungal community on decomposing leaf litter undergoes rapid successional changes.pdf

13632261 https://www.sysrev.com/p/104267/article/13632261 isacadavid9 Woody Perenne Pinus heldreichii Leaf All Europe Field None Fungi none NO ITS2 Grind the tissue In-house method Illumina Sequencing Others NCBI None None None NO Sampling sites None 2020 The community composition varied among different sites, but in this respect two sites at higher altitudes (harsh growing conditions) were separated from three sites at lower altitudes (milder growing conditions OTUs gITS7 and ITS4 Fungal Diversity in the Phyllosphere of Pinus heldreichii H. Christ-An Endemic and High-Altitude Pine of the Mediterranean Region.pdf Cengage, Suas A.; Licenciadas, Especificamente; Se, Isentam; De, Responsabilidade; Sobre, Qualquer; Garantia, Expressa; Ou Implícita, Sem; Limitações; Propósito, U M.; Persoh, Derek; Segert, Julia; Zigan, Anja; Rambold, Gerhard

13632263 https://www.sysrev.com/p/104267/article/13632263 carolinesalvati Woody Perenne Vitis riparia|||Vitis vinifera|||Vitis labrusca Leaf Endophitic North America Field Nutrient Fungi none NO ITS Grind the tissue Neasy Plant Mini Kit Roche 454 Sequencing QIIME||| USEARCH UNITE database None None None NO Plant genotypes|||Biotic stress None 2017 Wild plants have higher microbiome richness and supported a range of taxa not seen in conventional vineyards. Several isolates were inhibitory to grapevine pathogens Fungal endophytes of wild and hybrid Vitis leaves and their potential for vineyard biocontrol.pdf Vor, Jana; Baldrian, Petr; Vor ˇı ´s ˇkova ´, J

13632264 https://www.sysrev.com/p/104267/article/13632264 carolinesalvati Woody Perenne Avicennia marina|||Rhizophora stylosa Leaf||| Branches All Oceania Field None Fungi none NO ITS1||| ITS2 Grind the tissue Others Roche 454 Sequencing CD-HIT||| PANGEA Others None read filtering None NO aerieal x submerged parts of the plant|||Plant Specie None 2011 Genotype modulates microbiome in both aerial and intertidal parts of the trees Multiple markers pyrosequencing reveals highly diverse and host-specific fungal communities on the mangrove trees Avicennia marina and Rhizophora stylosa.pdf Arfi, Yonathan; Bue, Marc; Marchand, Cyril; Levasseur, Anthony; Record, Eric

13632265 https://www.sysrev.com/p/104267/article/13632265 isacadavid9 Woody Perenne Acacia hindsii Leaf Epyphitic Central America Field Biotic Bacteria 16S rDNA NO None Wash the tissue Others Roche 454 Sequencing QIIME Ribosomal Database Project None None None NO presence/absence of mutualistic or parasitic ants None 2014 indirect defence of mutualistic ants also covers the protection from bacterial plant pathogens OTUs none Mutualistic ants as an indirect defence against leaf pathogens.pdf Gonz Alez-Teuber, Marcia; Kaltenpoth, Martin; Boland, Wilhelm

13632266 https://www.sysrev.com/p/104267/article/13632266 erika_frydrych Woody Perenne Fraxinus excelsior Leaf||| other Endophitic Europe Field None Fungi none NO ITS2 Others E.Z.N.A. Plant DNA Kit Illumina Sequencing QIIME||| USEARCH||| R packages UNITE database None None FUNGuild NO . None 2021 . OTUs . Mycobiome of Fraxinus excelsior With Different Phenotypic Susceptibility to Ash Dieback.pdf

13632267 https://www.sysrev.com/p/104267/article/13632267 erika_frydrych Woody Perenne Fagus sylvatica Branches Endophitic Europe None None Fungi none NO ITS Others Charge Switch1 gDNA Plant kit (Invitrogen) Illumina Sequencing QIIME||| USEARCH Others None None None NO . None 2021 . OTUs . Mycobiomes of Young Beech Trees Are Distinguished by Organ Rather Than by Habitat, and Community Analyses Suggest Competitive Interactions Among Twig Fungi.pdf Ruotsalainen, Anna Liisa.; Hietala, Ari Mikko.; Siddique, Abu Bakar.; Biella, Paolo; Unterseher, Martin; Albrectsen, Benedicte Riber.

13632269 https://www.sysrev.com/p/104267/article/13632269 carolinesalvati Herbaceous Annual Triticum aestivum L.|||Triticum spelta L. Seed|||Root||| Leaf Endophitic Europe Greenhouse||| Farm None Bacteria 16S rDNA V3 - V4 NO None Grind the tissue PowerSoil DNA Isolation Kit Illumina Sequencing Others Ribosomal Database Project None None None YES Plant genotype|||Ecosystem Cytoscape 2020 The seed-born microbiome is not statistically significantly dependent on the wheat cultivars New Insight into the Composition of Wheat Seed Microbiota.pdf

13632270 https://www.sysrev.com/p/104267/article/13632270 isacadavid9 Herbaceous Perenne Colobanthus quitensis, Deschampsia antarctica Root||| Leaf||| RhizosphereEpyphitic||| Endophitic Antartica Field None Bacteria||| Archea 16S rDNA V4 NO None Grind the tissue||| Wash the tissue PowerSoil DNA Isolation Kit||| Zymobiomics DNA kit Illumina Sequencing MOTHUR||| QIIME||| R packages Greengenes database None read filtering FAPROTAX YES Plant compartment WGCNA 2020 Microbime had a niche differentation by plant comparment, and some taxa were biomarker for each niche OTUs None Niche Differentiation in the Composition, Predicted Function, and Co-occurrence Networks in Bacterial Communities Associated With Antarctic Vascular Plants.pdf

13632271 https://www.sysrev.com/p/104267/article/13632271 isacadavid9 Woody Perenne Metrosideros polymorpha, Cestrum nocturnum, Leaf Epyphitic North America Nurseries Biotic Fungi none NO ITS1 Others In-house method Illumina Sequencing QIIME Others None Microorganism specific primers None NO . None 2017 . OTUs ITS1F Not just browsing an animal that grazes phyllosphere microbes facilitates community heterogeneity.pdf O'rorke, Richard; Tooman, Leah; Gaughen, Kapono; Holland, Brenden S.; Amend, Anthony S.

13632274 https://www.sysrev.com/p/104267/article/13632274 carolinesalvati Woody Perenne Acacia raddiana|||Acacia tortilis Leaf Endophitic|||Epyphitic Asia Field Drought Bacteria 16S rDNA V1 - V3||| 16S rDNA V5 - V7 NO None Wash the tissue|||Grind the tissue PowerSoil DNA Isolation Kit Illumina Sequencing QIIME Greengenes database None read filtering None NO Geography|||Season|||Plant Species None 2021 nly a fewbacterial families dominate both epiphyte and endophyte communities; Epiphytic is more diverse que endophytic microbiome Temporal and Spatial Changes in Phyllosphere Microbiome of Acacia Trees Growing in Arid Environments.pdf Kumar, Upendra; Al Ashhab, Ashraf; Meshner, Shiri; Alexander-Shani, Rivka; Dimerets, Hana; Brandwein, Michael; Bar-Lavan, Yael; Winters, Gidon

13632275 https://www.sysrev.com/p/104267/article/13632275 carolinesalvati None Perenne Myrtillocactus geometrizans|||Opuntia robusta Stem||| Rhizosphere|||Root||| Bulk soil Epyphitic||| Endophitic Central America Field Drought Bacteria||| Archea||| Fungi 16S rDNA V4 NO ITS2 Wash the tissue|||Grind the tissue In-house method Illumina Sequencing Others Others None PNA None NO Plant Species|||Geography None 2016 Remarkably, bacterial, and archaeal diversity was higher in the phyllosphere than in the rhizosphere of Cacti, while the opposite was true for fungi. Influence of the plant host did only play a larger role in the fungal communities of the stem endosphere. The Cacti Microbiome Interplay between Habitat Filtering and Host-Specificity.pdf Prithiviraj, Bharath; Agler, Matthew; Partida-Martínez, Laila P.; Garrido, Etzel; Fonseca-García, Citlali; Coleman-Derr, Devin; Visel, Axel; Tringe, Susannah G.

13632276 https://www.sysrev.com/p/104267/article/13632276 carolinesalvati Woody Annual Fagus sylvatica Leaf All Europe Field None Fungi none NO ITS1 Grind the tissue Others Roche 454 Sequencing QIIME||| Others NCBI_fungi ITS database None None None NO geography (elevation) None 2012 Composition of fungal assemblages varied significantly between elevation sites, in terms of both the relative abundance and the presence-absence of species The composition of phyllosphere fungal assemblages of European beech (Fagus sylvatica) varies significantly along an elevation gradient.pdf

13632278 https://www.sysrev.com/p/104267/article/13632278 erika_frydrych Herbaceous Perenne Arabidopsis thaliana Seed||| other||| Leaf Epyphitic Europe Greenhouse None Bacteria 16S rDNA V5 - V7 NO None Grind the tissue Qiagen DNeasy Blood & Tissue Kit Roche 454 Sequencing MOTHUR||| R packages Ribosomal Database Project||| SILVA database None Microorganism specific primers None NO the effect of different growth substrates|||different tissues None 2015 Seed and radicle endophytic assemblages were similar; The leaf endophytic assemblage was mainly derived from the environment and not from the seed OTUs The effects of the growth substrate on cultivable and total endophytic assemblages of Arabidopsis thaliana.pdf Truyens, Sascha; Beckers, Bram; Thijs, Sofie; Weyens, Nele; Cuypers, Ann; Vangronsveld, Jaco

13632279 https://www.sysrev.com/p/104267/article/13632279 carolinesalvati Herbaceous Annual Arabidopsis thaliana Leaf Epyphitic Europe Greenhouse Biotic Bacteria 16S rDNA V5 - V7 NO None Wash the tissue PowerSoil DNA Isolation Kit Illumina Sequencing MOTHUR SILVA database||| Ribosomal Database Project None read filtering None NO Plant genotype|||Resistance to fungal pathogen None 2015 bdg-mutant plants are resistant to fungal pathogen Botrytis cinerea due to phyllosphere; lacs2.3 are resistant due to endogenous mechanisms The microbiome of the leaf surface of Arabidopsis protects against a fungal pathogen.pdfRitpitakphong, Unyarat; Falquet, Laurent; Vimoltust, Artit; Berger, Antoine; Etraux, Jean-Pierre M.; L'haridon, Floriane

13632280 https://www.sysrev.com/p/104267/article/13632280 isacadavid9 Woody Perenne Picea glauca Leaf All North America Field None Fungi none NO ITS2 Grind the tissue Others Illumina Sequencing Others Ribosomal Database Project||| UNITE database None None FUNGuild NO phenotypic tree traits, genetic variation, climate variables None 2019 Alpha-diversity declined with increasing mean annual precipitation and decreasing temperature, no effect was observed of the host genotype, The needle mycobiome of Picea glauca - A dynamic system reflecting surrounding environment and tree phenological traits.pdf Würth, David G.; Dahl, Mathilde Borg.; Trouillier, Mario; Wilmking, Martin; Unterseher, Martin; Scholler, Markus; Sørensen, Søren; Mortensen, Martin; Schnittler, Martin

13632281 https://www.sysrev.com/p/104267/article/13632281 isacadavid9 Woody Perenne Fraxinus excelsior, Sorbus aucuparia Leaf All North America Field Biotic Fungi none NO ITS Grind the tissue Neasy Plant Mini Kit PacBio sequencing MOTHUR UNITE database None None None NO Geopraphy, Season, Disease symtoms, washing treatment, tree species None 2020 the sampling site (country) was the most important factor The Relationship between Fungal Diversity and Invasibility of a Foliar Niche—The Case of Ash Dieback.pdf Agan, Ahto; Drenkhan, Rein; Adamson, Kalev; Tedersoo, Leho; Solheim, Halvor; Børja, Isabella; Matsiakh, Iryna; Timmermann, Volkmar; Nagy, Nina Elisabeth.; Hietala, Ari Mikko.

13632282 https://www.sysrev.com/p/104267/article/13632282 isacadavid9 Woody Perenne Brachythecium fendleri, Pinus leiophylla Leaf Endophitic North America Field None Fungi none NO ITS Grind the tissue Neasy Plant Mini Kit Roche 454 Sequencing MOTHUR NCBI None read filtering None NO tissue storage, primers, filtering method effect in diversity None 2014 storage in silica gel markedly limits the recovery of sequence data and yields a small fraction of the diversity Tissue storage and primer selection influence pyrosequencing-based inferences of diversity and community composition of endolichenic.pdf U'ren, Jana M.; Riddle, Jakob M.; Monacell, James T.; Carbone, Ignazio; Miadlikowska, Jolanta; E L I Z A B E T H A R N O L D, A

13632283 https://www.sysrev.com/p/104267/article/13632283 isacadavid9 Woody Perenne Malus domestica Leaf All Asia Greenhouse Biotic Fungi none NO None Grind the tissue Others Illumina Sequencing Others NCBI None read filtering None NO Gymnosporangium yamadae inoculation None 2020 G, yamadae infection may modify the fungal community composition Transcriptome Analysis of Apple Leaves Infected by the Rust Fungus Gymnosporangium yamadae at Two Sporulation Stages.pdf

13632284 https://www.sysrev.com/p/104267/article/13632284 isacadavid9 Woody Perenne Pseudowintera colorata Leaf|||Root||| Stem Endophitic Oceania Field None Bacteria 16S rDNA V3 - V4 NO None Grind the tissue Others Illumina Sequencing QIIME Greengenes database None None PICRUSt NO Plant tissue None 2020 owing tissue type as the main factor influencing the similarity and richness of endophytic bacteria Community Structure, Diversity and Potential of Endophytic Bacteria in the Primitive New Zealand Medicinal Plant Pseudowintera colora.pdf Purushotham, Neeraj; Jones, Eirian; Monk, Jana; Ridgway, Hayley

13632286 https://www.sysrev.com/p/104267/article/13632286 carolinesalvati Woody Perenne Populus balsamifera Leaf All North America Garden None Fungi none NO ITS1||| ITS2 Grind the tissue Others Roche 454 Sequencing Others NCBI_fungi ITS database None None None NO DNA Marker None 2012 ITS2 may be more variable and recovers more of the molecular diversity. we demonstrate that both ITS1 and ITS2 reveal similar patterns in community structure when analyzed in a community ecology context. Comparison of ITS1 and ITS2 rDNA in 454 sequencing of hyperdiverse fungal communities.pdf Bazzicalupo, Anna L.; Os B Alint, Mikl; Schmitt, Imke; Orn Lindahl, Bj€

13632287 https://www.sysrev.com/p/104267/article/13632287 carolinesalvati Herbaceous Annual Allium tuberosum Root||| Leaf||| Bulk soil Endophitic Asia Field None Bacteria 16S rDNA V3 - V4 NO None Grind the tissue Others Illumina Sequencing MOTHUR||| Others||| USEARCH SILVA database None None None NO Tissue None 2014 The rhizosphere bacterial community was significantly different from the endophytic bacterial communities. Endophytic leaf and roots communities are slightly similar. Comparison of rhizosphere and endophytic microbial communities of Chinese leek through high-throughput 16S rRNA gene Illumina sequencing.pdf Huang, Yong-Hong

13632290 https://www.sysrev.com/p/104267/article/13632290 carolinesalvati Herbaceous Annual Triticum aestivum L. (cv.'Hondia')|||Triticum spelta L. (cv. 'Rokosz') Root||| Leaf||| other Endophitic Europe Farm None Bacteria 16S rDNA V1 - V3 NO None Grind the tissue PowerSoil DNA Isolation Kit Illumina Sequencing Others Ribosomal Database Project None read filtering None NO Plant species|||Plant tissue None 2019 Pseudomonas was the only endophytic genus in both wheat species during all lifecycle Culture-independent analysis of an endophytic core microbiome in two species of wheat Triticum aestivum L. (cv. `Hondia') and the first report of microbiota in Triticum spelta L. (cv. `Rokosz').pdf Ikeda, Anzu; Matsuoka, Shunsuke; Masuya, Hayato; Mori, Akira S.; Hirose, Dai; Osono, Takashi; Matsuoka, Á S.; Osono, Á T.

13632291 https://www.sysrev.com/p/104267/article/13632291 carolinesalvati Herbaceous Annual Oryza sativa L Leaf Endophitic Asia Farm None Bacteria 16S rDNA V3 - V4 NO None Grind the tissue In-house method||| Others Illumina Sequencing||| Sanger Sequencing QIIME||| Others SILVA database None read filtering None NO Plant genotype (aromatic and non-aromatic)|||Geography None 2021 rice leaf endophytic microbiomes in mountain zonewere found clustered separately from that of plateau-zone. Deciphering core-microbiome of rice leaf endosphere Revelation by metagenomic and microbiological analysis of aromatic and non-aromatic genotypes grown in three geographical zones.pdf Kumar, Mukesh; Kumar, Aundy; Sahu, Kuleshwar Prasad.; Patel, Asharani; Reddy, Bhaskar; Sheoran, Neelam; Charishma, Krishnappa; Rajashekara, Hosahatti; Bhagat, Someshwar; Rathour, Rajeev

13632293 https://www.sysrev.com/p/104267/article/13632293 isacadavid9 Woody Perenne Citrus sinensis, Citrus paradisi, Citrus unshiu, Citrus x tangelo Root||| Leaf All North America Field Biotic Bacteria||| Archea 16S rDNA V4 NO None Grind the tissue Others Illumina Sequencing QIIME Greengenes database None read filtering None YES Effect of liberibacter spp. symtom severity, geographic location, citrus cultivar, season, time Cytoscape 2017 bacteria Infection changed core community structur that may be a precondition to disease or symptom progession Defining the Core Citrus Leaf- and Root-Associated Microbiota Factors Associated with Community Structure and Implications for Managing Huanglongbing (Citrus Greening) Disease.pdf

13632295 https://www.sysrev.com/p/104267/article/13632295 carolinesalvati Woody Perenne Vitis Vinifera Fruit||| Leaf Epyphitic Europe Farm None Bacteria||| Fungi 16S rDNA V4 NO ITS2 Wash the tissue Others Illumina Sequencing Others Ribosomal Database Project None None None NO Plant genotype|||Geography|||Season None 2018 Genotypes have an impact in microbial assemblahge of fruits and leaves in the same geography Genotype-Environment Interaction Shapes the Microbial Assemblage in Grapevine_s Phyllosphere and Carposphere An NGS Approach.pdf Singh, Prashant; Santoni, Sylvain; This, Patrice; Péros, Jean-Pierre

13632296 https://www.sysrev.com/p/104267/article/13632296 isacadavid9 Herbaceous Annual Hordeum vulgare Seed Epyphitic North America Farm None Fungi none NO ITS Wash the tissue In-house method Roche 454 Sequencing MOTHUR||| QIIME UNITE database None None None NO Geographic location, agronomical practices, two seasons None 2016 Geographic location was the main factor determining epiphytic fungal communities, agronomical practices had a significant impact Geography and agronomical practices drive diversification of the epiphytic mycoflora associated with barley and its malt end product.pdf Chen, Wen; Turkington, T Kelly.; Lévesque, C André.; Bamforth, Janice M.; Patrick, Susan K.; Lewis, Christopher T.; Chapados, Julie T.; Tittlemier, Sheryl A.; Macleod, Aaron; Gräfenhan, Tom

13632297 https://www.sysrev.com/p/104267/article/13632297 isacadavid9 Herbaceous Annual||| Perenne Arrhenatherum elatius, Galium album Leaf Epyphitic||| Endophitic Europe Field heat Bacteria 16S rDNA V3 - V4 NO None Wash the tissue|||Grind the tissue Others Illumina Sequencing CD-HIT SILVA database None blocking oligonucleotide None NO Heat stress treatment None 2020 Changes of the plant physiology and anatomy (stomata, metabolites) may have been responsible for the changes in composition and diversity of the studied leaf microbiota. Global warming shifts the composition of the abundant bacterial phyllosphere microbiota as indicated by a cultivation-dependent and -independent study of the grassland phyllosphere of a long-term warming field experiment.pdf Aydogan, Ebru L.; Budich, Olga; Hardt, Martin; Choi, Young Hae.; Jansen-Willems, Anne B.; Moser, Gerald; Müller, Christoph; Kämpfer, Peter; Glaeser, Stefanie P.

13632298 https://www.sysrev.com/p/104267/article/13632298 carolinesalvati Woody Perenne platanus acerifolia Leaf Epyphitic Europe Field||| Garden Chemicals Bacteria 16S rDNA V4 NO None Wash the tissue Others Illumina Sequencing Others Ribosomal Database Project||| Greengenes database None read filtering None NO Geography|||Exposure to air pollution None 2019 Air pollution exposure did not link with akoha diversity but it correlated with shifts in PBC composition in form of turnover (an equilivrium of taxa gain and taxa loss). Landspace composition sorrounding tress leads to taxa loss, expressed in lower phyllosphere alpha diversity Green infrastructure and atmospheric pollution shape diversity and composition of phyllosphere bacterial communities in an urban landscape.pdf

13632299 https://www.sysrev.com/p/104267/article/13632299 isacadavid9 Woody Perenne Picea glauca Leaf All North America Field None Fungi none NO ITS Grind the tissue Others Illumina Sequencing QIIME||| USEARCH UNITE database None None None NO environmental conditions, host genotype, phenological host traits, None 2016 local habitat conditions and phenotypic tree traits influence in inhabiting fungi, genotype did not influence fungal composition Habitat conditions and phenological tree traits overrule the influence of tree genotype.pdf Eusemann, Pascal; Schnittler, Martin; Nilsson, R Henrik.; Jumpponen, Ari; Dahl, Mathilde B.; W€ Urth, David G.; Buras, Allan; Wilmking, Martin; Unterseher, Martin

13632300 https://www.sysrev.com/p/104267/article/13632300 carolinesalvati Herbaceous Perenne Leymus chinesis|||Agropyron cristatum|||Stipa grandis Leaf Epyphitic Asia Field None Bacteria||| Archea||| Fungi 16S rDNA V3 - V4 NO ITS1 Wash the tissue PowerSoil DNA Isolation Kit Illumina Sequencing MOTHUR||| USEARCH||| QIIME SILVA database||| UNITE database None read filtering None YES Sampling habitat|||Geography Others 2019 Few microrganisms coexist in different samples, but they account for 60% of the total sequences, indicating their ability to adapt to variable environments. Habitat filtering shapes the differential structure of microbial communities in the Xilingol grassland.pdf Yang, Jie; Wang, Yanfen; Xiaoyong Cui; Xue, Kai; Zhang, Yiming; Yu, Zhisheng

13632302 https://www.sysrev.com/p/104267/article/13632302 carolinesalvati Herbaceous Annual Lactuca sativa var.capitata|||Lactuca sativa var.longifolia|||Spinacia oleracea Leaf||| Bulk soil||| other Epyphitic North America Farm None Bacteria 16S rDNA V5 - V7 NO None Wash the tissue PowerSoil DNA Isolation Kit Roche 454 Sequencing QIIME Greengenes database None Microorganism specific primers None NO Plant species|||Sampling method None 2016 The cultured communities from leaves are highly representative of the culture-independent communities, with over 85% of the prevalent OTUs recovered. High-Level Culturability of Epiphytic Bacteria and Frequency of Biosurfactant Producers on Leaves.pdf Burch, Adrien Y.; Do, Paulina T.; Sbodio, Adrian; Suslow, Trevor V.; Lindow, Steven E.

13632303 https://www.sysrev.com/p/104267/article/13632303 carolinesalvati Herbaceous Annual Triticum aestivum|||Hordeum vulgare|||Avena sativa|||Secale cereale|||Triticale (Triticum x Secale) Leaf All Europe Farm Chemicals Fungi none NO ITS1 Grind the tissue Others Roche 454 Sequencing QIIME UNITE database||| NCBI_fungi ITS database None Microorganism specific primers None NO host genotype|||fungicide treatment|||location None 2015 Host genotype at both the species and cultivar level is important in shaping phyllosphere fungal communities, whereas fungicide treatment and location have minor effects. Host genotype is an important determinant of the cereal phyllosphere mycobiome.pdf Sapkota, Rumakanta; Knorr, Kamilla; Jørgensen, Lise Nistrup.; O'hanlon, Karen A.; Nicolaisen, Mogens

13632304 https://www.sysrev.com/p/104267/article/13632304 isacadavid9 Woody Perenne Prunus persica Flower||| Leaf||| Stem|||Root||| Rhizosphere Endophitic Asia Farm None Fungi none NO ITS1 Grind the tissue In-house method||| Others Illumina Sequencing MOTHUR UNITE database None None None NO Plant cultivar, Plant tissues, None 2019 Root had the highest fungal richness and diversity, folloed by flowers, leaves the lowest, fungal communities of the cultivars are different, correlated with soil properties, and fruit properties Organs, Cultivars, Soil, and Fruit Properties Affect Structure of Endophytic Mycobiota of Pinggu Peach Trees.pdf Ren, Fei; Dong, Wei; Yan, Dong-Hui

13632305 https://www.sysrev.com/p/104267/article/13632305 carolinesalvati Herbaceous Perenne Artemisia argyi Leaf Epyphitic Asia Field Chemicals Bacteria none Yes None Others In-house method Illumina Sequencing Others Ribosomal Database Project None None None NO Stress|||Geography None 2021 structure and function of phyllosphere microbiota are globally impacted by haze, while primary and secondary metabolites responsible for haze tolerance were considerably increased. Pathological Impact on the Phyllosphere Microbiota of Artemisia argyi by Haze.pdf Zhang, Yu-Zhu; Jiang, De-Yu; Zhang, Chi; Yang, Kun; Wang, Huai-Fu; Xia, Xiu-Wen; Ding, Wei-Jun

13632307 https://www.sysrev.com/p/104267/article/13632307 carolinesalvati Woody Perenne Populus tremula|||Populus alba Rhizosphere||| Stem|||Root||| Leaf Endophitic Europe Farm None Bacteria 16S rDNA V5 - V7||| 16S rDNA V3 - V4||| 16S rDNA V1 - V4||| 16S rDNA V1 - V3 NO None Grind the tissue PowerSoil DNA Isolation Kit Roche 454 Sequencing MOTHUR SILVA database None Microorganism specific primers None NO 16S Primers|||Plant Species Others 2016 Primer pair 799F-1391R outperforms all other primer pairs in our study in the elimination of non-target DNA and retrieval of bacterial OTUs Performance of 16s rDNA Primer Pairs in the Study of Rhizosphere and Endosphere Bacterial Microbiomes in Metabarcoding Studies.pdf Gourion, Benjamin; Hervé, Sanguin; Mondy, Samuel; Op, Michiel; Beeck, De; Beckers, Bram; De Beeck, Michiel Op.; Thijs, Sofie; Truyens, Sascha; Weyens, Nele; Boerjan, Wout; Vangronsveld, Jaco

13632308 https://www.sysrev.com/p/104267/article/13632308 carolinesalvati Woody Perenne Citrus sinensis L.Osbeck Leaf Epyphitic||| Endophitic South America Farm Drought Bacteria 16S rDNA V3 - V4 NO None Wash the tissue|||Grind the tissue In-house method Illumina Sequencing QIIME SILVA database None read filtering None NO Management Systems|||Season None 2020 high copper concentrations may have influenced bacterial abundance, having a relevant impact on the differences observed Phyllosphere bacterial assembly in citrus crop under conventional and ecological management.pdf Carvalho, Carolinne R.; Dias, Armando Cf.; Homma, Sérgio K.; Cardoso, Elke Jbn.; Tiessen, Axel

13632309 https://www.sysrev.com/p/104267/article/13632309 carolinesalvati Herbaceous Perenne Wolffia australiana Leaf Epyphitic Asia Greenhouse None Bacteria 16S rDNA V3 NO None Wash the tissue FastDNA Spin Kit for Soil Illumina Sequencing QIIME Ribosomal Database Project||| Greengenes database None read filtering None NO Origin of paddy soil None 2015 The compositional structures of the phyllosphere bacterial communities were modulated predominantly by water physicochemical properties, while the initial soil bacterial communities had limited impact Phyllosphere Bacterial Community of Floating Macrophytes in Paddy Soil Environments as Revealed by Illumina High-Throughput Sequencing.pdf

13632310 https://www.sysrev.com/p/104267/article/13632310 carolinesalvati Woody Perenne Aegiceras corniculatum|||Avicennia marina|||Bruguiera gymnorrhiza|||Kandelia candel|||Rhizophora stylosa|||Excoecaria agallocha Leaf Epyphitic||| Endophitic Asia Field None Fungi none NO ITS2 Wash the tissue|||Grind the tissue In-house method Illumina Sequencing QIIME||| MOTHUR||| USEARCH UNITE database None None None YES Plant genotype|||Plant compartment Cytoscape 2019 This study reveals that the phyllosphere epiphytic and endophytic fungal communities differ, and plant identity has a greater effect on the endophytic fungi than on epiphytic fungi. Phyllosphere epiphytic and endophytic fungal community and network structures differ in a tropical mangrove ecosystem.pdf Yao, Hui; Sun, Xiang; He, Chao; Maitra, Pulak; Li, Xing-Chun; Guo, Liang-Dong; Bruguiera Gymnorrhiza Rhizophora Stylosa Kandelia; Fdr <, P

13632311 https://www.sysrev.com/p/104267/article/13632311 isacadavid9 Woody Perenne Prunus domestica Leaf||| Fruit Epyphitic Europe Farm Biotic Fungi none NO ITS2 Wash the tissue Others Illumina Sequencing QIIME SILVA database None None None NO Different cultivars, Different tissues, Different seasons None 2019 The composition and diversity of fungal communities varied between cultivars and sampling seasons Phyllosphere Fungal Communities of Plum and Antifungal Activity of Indigenous Phenazine-Producing Pseudomonas synxantha Against Monilinia laxa.pdf Janisiewicz, Wojciech Jerzy.; Beri, Tanja; Janakiev, Tamara; Dimki, Ivica; Unkovi, Nikola; Ljaljevi Ć Grbi, Milica; Opsenica, Dejan; Gaši, Uroš; Stankovi, Slaviša

13632313 https://www.sysrev.com/p/104267/article/13632313 isacadavid9 Herbaceous Perenne Agave tequilana, Agave salmiana, Agave deserti Rhizosphere||| Leaf|||Root||| Bulk soil Endophitic|||Epyphitic Central America||| North America Field||| Farm None Bacteria||| Fungi||| Archea 16S rDNA V4 NO ITS2 Wash the tissue|||Grind the tissue In-house method||| PowerSoil DNA Isolation Kit Illumina Sequencing USEARCH Ribosomal Database Project None PNA None NO Plant compartment, Plant biogeography, cultivated and native plant None 2016 Prokaryotic communities was determined by plant compartment, fungal communities was mainly influenced by biogeography of host species, Cultivated A. tequilana exhibited lower levels of prokaryotic diversity compared with native agaves Plant compartment and biogeography affect microbiome composition in cultivated and native Agave species.pdf Coleman-Derr, Devin; Desgarennes, Damaris; Fonseca-Garcia, Citlali; Gross, Stephen; Clingenpeel, Scott; Woyke, Tanja; North, Gretchen; Visel, Axel; Partida-Martinez, Laila P.; Tringe, Susannah G.

13632314 https://www.sysrev.com/p/104267/article/13632314 isacadavid9 Herbaceous Annual Gossypium hirsutum Leaf Epyphitic Asia Greenhouse None Fungi none NO 18S rRNA Wash the tissue FastDNA Spin Kit for Soil Roche 454 Sequencing MOTHUR SILVA database None None None NO Plant developmental stage, transgenic bt cotton, non-transgenic cotton None 2019 Developmental stage rather than Cry1Ac expression was the key factor shaping the phyllosphere mycobiome in transgenic cotton Developmental stage has a greater effect than Cry1Ac expression in transgenic cotton on the phyllosphere mycobiome.pdf Pan, Jiangang; Lv, Xin; Jin, Decai; Bai, Zhihui; Qi, Hongyan; Zhang, Hongxun; Zhuang, Guoqiang

13632315 https://www.sysrev.com/p/104267/article/13632315 isacadavid9 Woody Perenne Ilex anomala, Melicope clusiifolia, Broussaicia arguta, Metrosideros polymorpha, Leaf Epyphitic North America Field None Fungi none NO ITS1 Wash the tissue PowerSoil DNA Isolation Kit Illumina Sequencing USEARCH||| MOTHUR UNITE database None None None NO Snail host plant species, Feaces of snaill, geographic distance None 2016 composition of fungal communities in feces differed by proportion from what was available on leaves, Community dissimilarity was significantly correlated with geographic distance Diet selection at three spatial scales Implications for conservation of an endangered Hawaiian tree snail.pdf Price, Melissa R.; O'rorke, Richard; Amend, Anthony S.; Hadfield, Michael G.

13632316 https://www.sysrev.com/p/104267/article/13632316 isacadavid9 Herbaceous Annual Raphanus sativus Seed Epyphitic Europe Greenhouse Biotic Bacteria none Yes None Wash the tissue PowerSoil DNA Isolation Kit Illumina Sequencing||| PacBio sequencing Kraken NCBI None read filtering Prokka NO Influence of two phytopathogenic agents on seed microbiome None 2019 No shift in structure and function of the seed microbiome was observed after phytopathogen inoculation Differences in resource use lead to coexistence of seed-transmitted microbial populations.pdf

13632317 https://www.sysrev.com/p/104267/article/13632317 isacadavid9 Woody Perenne Pisonia umbellifera, Pisonia sandwicensis, Pittosporum glabra, Pouteria sandwicensis, Psychotria sp., Schinus terebinthifolius, Smilax sp., Psidium cattleianum, Urera glabra, Urera kaalae, Pisonia brunoniana, Pipturus albidus, Perrottetia sandwicensis, Perrottetia sp, Nestegis sandwicensis, Myrsine sp., Myrsine lessertiana, Metrosideros polymorpha, Melicope oahuensis, Ilex anomala, Freycinetia arborea, Hedyotis terminalis, Alyxia oliviformis, Antidesma sp., Claoxylon sp., Broussaisia arguta, Coprosma longiflora, Dianella sandwicensis, Diospyros sandwicensis, Leaf Epyphitic North America Field None Fungi|||Bacteria 16S rDNA V4 NO ITS1 Wash the tissue PowerSoil DNA Isolation Kit Illumina Sequencing QIIME||| USEARCH||| MOTHUR Greengenes database||| UNITE database None None None NO Snail host plant species, Feces of snail None 2014 Location, sample type (feces or leaf) and host plant explained the microbial community composition Dining local the microbial diet of a snail that grazes microbial communities is geographically structured.pdf O'rorke, Richard; Cobian, Gerald M.; Holland, Brenden S.; Price, Melissa R.; Costello, Vincent; Amend, Anthony S.

13632318 https://www.sysrev.com/p/104267/article/13632318 isacadavid9 Woody Perenne Mussaenda pubescens Leaf All Asia Field None Fungi none NO ITS2 Grind the tissue In-house method Illumina Sequencing QIIME||| USEARCH UNITE database None None None NO host plant genotype, geographic distance None 2018 Fungal community composition was significantly structured by host genotype, and less by geographic distance Host genotype strongly influences phyllosphere fungal communities associated with Mussaenda pubescens var. alba (Rubiaceae).pdf Qian, Xin; Duan, Tingting; Sun, Xiang; Zheng, Yong; Wang, Yonglong; Hu, Meiling; Yao, Hui; Ji, Niuniu; Lv, Pengpeng; Chen, Liang; Shi, Miaomiao; Guo, Liangdong; Zhang, Dianxiang; Boddy

13632319 https://www.sysrev.com/p/104267/article/13632319 isacadavid9 Woody Perenne Aegiceras corniculatum, Avicennia marina, Bruguiera gymnorrhiza, Kandelia candel, Rhizophora stylosa, Excoecaria agallocha Leaf Epyphitic||| Endophitic Asia Field None Bacteria 16S rDNA V4 NO None Wash the tissue|||Grind the tissue In-house method Illumina Sequencing MOTHUR||| USEARCH Ribosomal Database Project None read filtering None YES Plant compartment, plant species Cytoscape||| Modular 2020 phyllosphere epiphytic and endophytic bacterial community structures differ and plant identity has a greater effect Host identity is more important in structuring bacterial epiphytes than endophytes in a tropical mangrove forest.pdf Yao, Hui; Sun, Xiang; He, Chao; Li, Xing-Chun; Guo, Liang-Dong; Garbeva, Paolina

13632320 https://www.sysrev.com/p/104267/article/13632320 isacadavid9 Herbaceous Perenne Aechmea aquilega, Aechmea mertensii Root||| Seed Endophitic South America Greenhouse None Fungi none NO ITS1 Grind the tissue Neasy Plant Mini Kit Illumina Sequencing MOTHUR||| USEARCH Others None None None NO Fungal in seed and seedling, in two species, Fungal fffect in seed germination and survival None 2019 Fungi are similar in seed and seedling then they are transmitted vertically How significant are endophytic fungi in bromeliad seeds and seedlings Effects on germination, survival and performance of two epiphyt.pdf Eline Leroy, C; Maes, Arthur Quymanh.; Louisanna, Eliane; Ejalon-Delmas, Nathalie S.

13632321 https://www.sysrev.com/p/104267/article/13632321 isacadavid9 Woody Perenne Coffea Leaf All Central America Field Biotic Fungi none NO ITS Grind the tissue Neasy Plant Mini Kit PacBio sequencing MOTHUR UNITE database None None None NO Hemileia vastatrix infection, Geography locations None 2016 Control samples tended to have more OTUs than samples from infected leaves, Geography was shown to be a greater determinant of fungal community structure than infection status Identification of Putative Coffee Rust Mycoparasites via Single-Molecule DNA Sequencing of Infected Pustules.pdfJames, Timothy Y.; Marino, John A.; Perfecto, Ivette; Vandermeer, John

13632322 https://www.sysrev.com/p/104267/article/13632322 isacadavid9 Herbaceous Annual Lactuca sativa Leaf||| Rhizosphere|||Root||| Bulk soil Epyphitic||| Endophitic North America Greenhouse None Bacteria none Yes None Wash the tissue|||Grind the tissue PowerSoil DNA Isolation Kit Illumina Sequencing MetaPhlAn ChocoPhlAn database None None None NO Soil fertilization with manure None 2021 Manure application affects the microbiome and resistome of both soil and lettuce Transmission Routes of the Microbiome and Resistome from Manure to Soil and Lettuce.pdf Sun, Yuepeng; Snow, Daniel; Walia, Harkamal; Li, Xu

13632324 https://www.sysrev.com/p/104267/article/13632324 isacadavid9 Woody Perenne Acer saccharum, Acer rubrum, Betula papyrifera, Abiesbalsamea, Picea glauca Leaf Epyphitic North America Field None Bacteria 16S rDNA V4 - V5 NO None Wash the tissue PowerSoil DNA Isolation Kit Illumina Sequencing QIIME||| USEARCH Greengenes database None Microorganism specific primers None NO Bacteria diversity and composition in different individual trees, species, and canopy strata None 2016 There is intra-individual and inter-specific variation, canopy location was not significant Tree phyllosphere bacterial communities exploring the magnitude of intra- and inter-individual variation among host species.pdf Laforest-Lapointe, Isabelle; Messier, Christian; Kembel, Steven W.

13632325 https://www.sysrev.com/p/104267/article/13632325 isacadavid9 Woody Perenne Clermontia kakeana, Clermontia calophylla, Clermontia kohalae, Clermontia clermoniotides, Clermontia peleana ssp, Clermontia singulariflora, Clermontia fauriei, Clermontia oblongifolia, Clermontia arborescens Leaf All North America Field None Fungi none NO ITS1 Grind the tissue In-house method Illumina Sequencing QIIME UNITE database None read filtering None NO Fungal diversity associated with eight Clermontia species None 2017 Biological collections are usefule to study microbial diversity Uncovering unseen fungal diversity from plant DNA banks.pdf Datlof, Erin M.; Amend, Anthony S.; Earl, Kamala; Hayward, Jeremy; Morden, Clifford W.; Wade, Rachael; Zahn, Geoffrey; Hynson, Nicole A.

13632327 https://www.sysrev.com/p/104267/article/13632327 isacadavid9 Herbaceous Perenne Medicago sativa Leaf Endophitic|||Epyphitic North America Field None Fungi||| Protist none NO 18S rRNA Grind the tissue||| Wash the tissue PowerSoil DNA Isolation Kit Illumina Sequencing Others SILVA database||| Protist Ribosomal Reference database None PNA None NO Effect of m16S, p16S and 18S PNA, to decrease host DNA amplification None 2020 gPNA increased microbial eukaryotic ASV richness within the M. sativa Using the Microbiome Amplification Preference Tool (MAPT) to Reveal Medicago sativa-Associated Eukaryotic Microbes.pdf Moccia, Katherine; Papoulis, Spiridon; Willems, Andrew; Marion, Zachary; Fordyce, James A.; Lebeis, Sarah L.

13632328 https://www.sysrev.com/p/104267/article/13632328 isacadavid9 Herbaceous Annual Zea mays, Oryza sativa, Triticum aestivum, Hordeum vulgare, Sorghum bicolor, Secale cereale, Avena sativa Rhizosphere Epyphitic North America Field None Fungi||| Protist||| Animal none NO 18S rRNA Wash the tissue PowerSoil DNA Isolation Kit Illumina Sequencing MOTHUR Protist Ribosomal Reference database||| SILVA database None PNA None NO Effect of PNA clamp for reducing plant reads and increase eukaryotic phytobiome diversity None 2020 PoacV9_01 reduced the relative abundance of five crop species reads Validation of a PNA Clamping Method for Reducing Host DNA Amplification and Increasing Eukaryotic Diversity in Rhizosphere Microbiome Studies.pdf Taerum, Stephen J.; Steven, Blaire; Gage, Daniel J.; Triplett, Lindsay R.

13632329 https://www.sysrev.com/p/104267/article/13632329 isacadavid9 Woody Perenne Acer saccharum Root||| Leaf||| Rhizosphere Epyphitic||| Endophitic North America Field None Bacteria||| Fungi 16S rDNA V5 - V6 NO ITS Wash the tissue PowerSoil DNA Isolation Kit Illumina Sequencing QIIME||| USEARCH Ribosomal Database Project None Microorganism specific primers None NO Tissue, Compartment, Plant elevational range, None 2018 Variation in microbial communities differed among plant components, Variation in the leaf and root microbiome of sugar maple (Acer saccharum) at an elevational range limit.pdf Wallace, Jessica; Laforest-Lapointe, Isabelle; Kembel, Steven W.

13632330 https://www.sysrev.com/p/104267/article/13632330 isacadavid9 Herbaceous Annual Cucumis sativus Leaf Epyphitic Asia Greenhouse Biotic Bacteria||| Fungi 16S rDNA V5 - V6 NO ITS Wash the tissue FastDNA Spin Kit for Soil Illumina Sequencing USEARCH Ribosomal Database Project||| UNITE database None None None YES Pseudomonas syringae pv. lachrymans treatment effect in microbial community Cytoscape 2019 the overall bacterial community tends toward mutualism from the competition OTUs gITS7 (Ihrmark et al. 2012) and ITS4 (White et al. 1990) Variations in phyllosphere microbial community along with the development of angular leaf-spot of cucumber.pdf Luo, Luyun; Zhang, Zhuo; Wang, Pei; Han, Yongqin; Jin, Decai; Tan, Xinqiu; Zhang, Deyong; Muhammad-Rizwan, Hamid; Lu, Xiangyang; Liu, Yong

13632331 https://www.sysrev.com/p/104267/article/13632331 isacadavid9 Woody Perenne Sequoia sempervirens Leaf Epyphitic||| Endophitic North America Field None Fungi none NO ITS1 Grind the tissue Others Illumina Sequencing USEARCH NCBI_fungi ITS database None None None NO Vertical stratification in the fungal community None 2016 Different portions of the tree crown harbored different assemblages of fungi Vertical stratification of the foliar fungal community in the world_s tallest trees.pdf Harrison, Joshua G.; Forister, Matthew L.; Parchman, Thomas L.; Koch, George W.; Premise

13632332 https://www.sysrev.com/p/104267/article/13632332 isacadavid9 Herbaceous Perenne Panax ginseng Rhizosphere Epyphitic Asia Field None Bacteria 16S rDNA V3 - V4 NO None Grind the tissue PowerSoil DNA Isolation Kit Illumina Sequencing QIIME Greengenes database||| Ribosomal Database Project None None None NO Effect of PGPB on the rhiziospheric bacteria None 2018 PGPB increased plant biomass, amount of ginsenosides and abundance of specific bacteria. Plant growth promoting bacteria increases biomass, effective constituent, and modifies rhizosphere bacterial communities of Panax.pdf Jin, Zhengxun; Li, Hulin; Ji, Wenxiu; Leng, Xue

13632333 https://www.sysrev.com/p/104267/article/13632333 carolinesalvati Woody Annual Alseis blackiana|||Desmopsis panamensis|||Heisteria concinna|||Sorocea affinis|||Tetragastris panamensis Leaf Endophitic Central America Field Chemicals Bacteria 16S rDNA V5 - V6 NO None Grind the tissue PowerSoil DNA Isolation Kit Illumina Sequencing QIIME Greengenes database None Microorganism specific primers None NO Plant Species|||Occurance of core microbiome|||High levels of macronutrients (N,P, K)|||Antibiotic effect None 2019 Antibiotics explained more endophy te variation than all nutrient addition combinations combined and t wice the variation explained by host identity for all five tree specie Plant host identity and soil macronutrients explain little variation in sapling endophyte community composition Is disturbance an alternative explanation.pdf Griffin, Eric A.; Harrison, Joshua G.; Kembel, Steven W.; Carrell, Alyssa A.; Wright, | S Joseph.; Carson, Walter P.; De Vries, Franciska; Wright, S Joseph.

13632334 https://www.sysrev.com/p/104267/article/13632334 carolinesalvati Herbaceous Annual Actinidia chinensis|||Actinidia deliciosa Leaf||| Flower Epyphitic Europe Farm Biotic Bacteria 16S rDNA V3 - V4 NO None Wash the tissue Others Illumina Sequencing MOTHUR||| Others SILVA database||| Ribosomal Database Project None read filtering None NO Infection with Pseudomonas syringae None 2018 Psa infection affected the phyllosphere microbiome structures in both species, however, its impact was more pronounced onA. deliciosaleaves, where a drastic drop in microbial biodiversity was observed. t Psa was always present with Pseudomonas syringaepv. syringae and Pseudomonas viridiflava Plant Microbiome and Its Link to Plant Health Host Species, Organs and Pseudomonas syringae pv. actinidiae Infection Shaping Bacteria.pdf Ntoukakis, Vardis; Arnold, Dawn; Kvitko, Brian H.; Spinelli, Francesco; Purahong, Witoon; Orrù, Luigi; Donati, Irene; Perpetuini, Giorgia; Cellini, Antonio; Lamontanara, Antonella; Michelotti, Vania; Tacconi, Gianni

13632335 https://www.sysrev.com/p/104267/article/13632335 isacadavid9 Woody Perenne Picea likiangensis var. rubescens, Picea smithiana, Picea abies, Picea crassifolia, Picea koraiensis, Picea wilsonii Leaf||| Rhizosphere||| Stem Epyphitic Asia Garden None Bacteria||| Archea||| Fungi 16S rDNA V4 NO ITS Wash the tissue PowerSoil DNA Isolation Kit Illumina Sequencing QIIME||| USEARCH SILVA database||| UNITE database None None None YES Plant phenotype traits, Plant species Cytoscape 2018 Microbiota and host plant phenotypic character are correlatedment of plant phenotypes Plant Phenotypic Traits Eventually Shape Its Microbiota A Common Garden Test.pdf Da Costa, Pedro Beschoren.; Li, Yunshi; Wu, Xiukun; Chen, Tuo; Wang, Wanfu; Liu, Guangxiu; Zhang, Wei; Li, Shiweng; Wang, Minghao; Zhao, Changming; Zhou, Huaizhe; Zhang, Gaosen

13632336 https://www.sysrev.com/p/104267/article/13632336 carolinesalvati Woody Perenne Hopea ferrera|||Tarenna sp.|||Memecylon ovatum|||Walsura trichostemon|||Strychnos kerrii|||Hydnocarpus ilicifolius|||Vatica cinerea|||Luvunga scandens|||Melodorum sp.|||Walsura robusta|||Shorea henryana|||Schefflera bengalensis|||Dialium cochinchinense|||Grewia paniculata|||Memecylon caeruleum|||Ancistrocladus tectorius|||Alchornea rugosa|||Aglaia pirifera|||Dehaasia candolleana|||Lygodium sp.|||Chaetocarpus castanocarpus|||Melodinus cambodiensis|||Fagerlindia sinensis|||Prismatomeris tetrandra|||Melodorum rutecensis|||Grewia panicurata|||Unindentified Leaf Endophitic Asia Field None Fungi none NO ITS1 Grind the tissue In-house method Ion Torrent Sequencing QIIME||| USEARCH NCBI_fungi ITS database None read filtering None NO Plant Height None 2016 Species diversity of phyllosphere fungi was the highest in the lowestr layers closest to the forest floor, decreased with increasing height, and lowest in the canopy. The fungal diversity in the canopy consisted of similar OTUs across plant individuals Vertical Structure of Phyllosphere Fungal Communities in a Tropical Forest in Thailand Uncovered by High-Throughput Sequencing.pdf

13632338 https://www.sysrev.com/p/104267/article/13632338 carolinesalvati Woody Perenne Fagus sylvatica Leaf Endophitic Europe Farm None Fungi none NO ITS1 Grind the tissue In-house method||| Others Illumina Sequencing||| Sanger Sequencing QIIME Others||| UNITE database None None None NO Methods None 2017 Undeniable advantages of HTS over cultivation in terms of revealing a good representation of the major functional guilds, rare taxa and biodiversity signals of leaf-inhabiting fungi. What do we learn from cultures in the omics age High-throughput sequencing and cultivation of leaf-inhabiting endophytes from beech.pdf

93777959 https://www.sysrev.com/p/104267/article/93777959 erika_frydrych Woody Perenne Hevea brasiliensis Leaf Epyphitic||| Endophitic Asia Field None Bacteria||| Fungi 16S rDNA V4 NO ITS1 Others FastDNA Spin Kit for Soil Illumina Sequencing QIIME||| R packages Others None read filtering None NO plant compartment|||geographical location|||seasonal changes None 2022 main fac- tor influencing endophytic bacteria were internal factors. External factors were the main driving force for epiphytic bacteria community assembly OTUs Phyllosphere fungal communities of rubber trees exhibited biogeographical patterns, but not bacteria.pdf Siddique, Abu Bakar.; Khokon, Anis Mahmud.; Unterseher, Martin; Schmitt, I

93777960 https://www.sysrev.com/p/104267/article/93777960 isacadavid9 Herbaceous Annual rice Leaf||| Seed Epyphitic Asia Field None Bacteria 16S rDNA NO None Wash the tissue In-house method||| Others Illumina Sequencing QIIME NCBI||| Ribosomal Database Project||| SILVA database None None None YES tissue, plant genotype Others 2023 The microbiome profiles were nearly identical on the aromatic and non-aromatic rice Fine-scale mapping of the microbiome on phylloplane and spermoplane of aromatic and non-aromatic rice genotypes.pdf

93777961 https://www.sysrev.com/p/104267/article/93777961 isacadavid9 Woody Perenne Platanus acerifolia Leaf||| Branches||| Trunk Epyphitic Europe Field None Bacteria 16S rDNA V4 NO None Wash the tissue QIAmp Powerfecal DNA Kit Illumina Sequencing R packages EzTaxon-e database None None None NO Plant tissue, Time point, sample location None 2022 Phyllosphere community composition was most strongly determined by tree compartment., Only the com- munities on the emerging leaves showed changing dynamics over time ASVs Bacterial Succession and Community Dynamics of the Emerging Leaf Phyllosphere in Spring.pdf

93777962 https://www.sysrev.com/p/104267/article/93777962 isacadavid9 Woody||| Herbaceous Perenne Calocedrus decurrens,Pinus ponderosa,Pinus lambertiana, Quercus kelloggii, Pteridium aquilinum Leaf Endophitic North America Field None Fungi none NO ITS1 Grind the tissue In-house method Illumina Sequencing R packages NCBI_fungi ITS database None read filtering None NO Plant species None 2022 abundant plant species harbored endophytes that occupied fewer plant species (higher structural specificity) and were consistently found in the same plant species across the landscape (higher beta-specificity). ASVs Plant abundance, but not plant evolutionary history, shapes patterns of host specificity in foliar fungal endophytes.pdf

93777963 https://www.sysrev.com/p/104267/article/93777963 isacadavid9 Herbaceous Annual Arabidopsis thaliana, Pisum sativum,Helianthus annuus, Coffea arabica, Glycine max, Manihot esculenta, Solanum lycopersicum, Phaseolus vulgaris, Saccharum officinarum, Hordeum vulgar, Brachypodium distachyon, Oryza sativa, Triticum aestivum, Panicum virgatum, Zea mays, Sorghum bicolor, Brachiaria decumbens, Rhizosphere|||Root||| Leaf||| Seed||| Stem Endophitic|||Epyphitic North America||| South America Greenhouse None Bacteria||| Fungi 16S rDNA V3 - V4 NO ITS Wash the tissue Neasy Plant Mini Kit Illumina Sequencing USEARCH||| Others Ribosomal Database Project None PNA None NO Plant species, Plant tissue, sterile sand, field soil None 2022 ars. Seeds and spermospheres contained some uncommon/abundant bacteria and many fungi, suggesting at least some of the rare microbiome is vertically transm OTUs Stochastic Inoculum, Biotic Filtering and Species-Specific Seed Transmission Shape the Rare Microbiome of Plants.pdf

93777964 https://www.sysrev.com/p/104267/article/93777964 erika_frydrych Woody Perenne Abies concolor Branches Endophitic Europe Field None Fungi none NO ITS2 Wash the tissue In-house method PacBio sequencing Others Others None None None NO . None 2022 . OTUs . Cytospora friesii and Sydowia polyspora are associated.pdf

93777965 https://www.sysrev.com/p/104267/article/93777965 erika_frydrych Woody Perenne Fagus sylvatica Root||| Leaf||| Rhizome Epyphitic Europe Nurseries Biotic Bacteria 16S rDNA V3 - V4 NO None Wash the tissue Others Illumina Sequencing MOTHUR SILVA database None None None NO Biotic None 2022 aphid infestation OTUs . Woolly beech aphid infestation reduces soil organic carbon availability and alters phyllosphere and rhizosphere bacterial microbiomes.pdf

93777966 https://www.sysrev.com/p/104267/article/93777966 isacadavid9 Herbaceous Perenne Azolla imbricata, Azolla pinnata, Azolla filiculoides, Azolla mexicana, Azolla caroliniana. Leaf Endophitic Asia Greenhouse None Bacteria 16S rDNA V5 - V7 NO None Grind the tissue Others Illumina Sequencing QIIME||| R packages Ribosomal Database Project None None None NO Plant species None 2022 the alpha diversity of bacteria was ordered as Azolla imbricata > A. pinnata > A. filiculoides > A. mexicana > A. caroliniana, The dominant bacteria of Azolla mainly belonged to the phylum of Proteobacteria OTUs Comparative analysis of the endophytic bacteria inhabiting the phyllosphere of aquatic fern Azolla species by high-throughput sequencing.pdf

93777967 https://www.sysrev.com/p/104267/article/93777967 erika_frydrych Herbaceous Annual Spinacia oleracea|||Lactuca sativa|||Allium sativum|||Coriandrum sativum|||Amaranthus tricolor Leaf Endophitic Asia Farm None Bacteria 16S rDNA V4 NO None Grind the tissue FastDNA Spin Kit for Soil Illumina Sequencing R packages||| Others Ribosomal Database Project||| SILVA database None None None NO . None 2021 . ASVs . Diverse antibiotic resistance genes and potential pathogens inhabit in the.pdf

93777968 https://www.sysrev.com/p/104267/article/93777968 erika_frydrych Herbaceous Annual Astragalus sinicus|||Vicia villosa Flower||| Leaf|||Root||| other Endophitic Asia Field None Fungi 16S rDNA V4 NO None Grind the tissue In-house method Illumina Sequencing QIIME||| VSEARCH||| Others SILVA database None None FUNGuild NO Mycobiome of green manure crops using a culture-dependent method None 2023 Most species and genera recorded from the High Throughput Sequencing approach were not obtained in the culture-dependent method OTUs Endophytic fungi in green manure crops; friends or foe?.pdf

93777969 https://www.sysrev.com/p/104267/article/93777969 erika_frydrych Herbaceous Perenne Leymus secalinus Root||| Leaf||| Stem Endophitic Asia Field None Bacteria 16S rDNA V5 - V6||| 16S rDNA V3 - V4 NO None Wash the tissue PowerSoil DNA Isolation Kit Illumina Sequencing R packages||| VSEARCH Ribosomal Database Project||| SILVA database None None PICRUSt NO ; None 2023 ; OTUs ; The Zoige pioneer plant Leymus secalinus has different endophytic bacterial community structures to adapt to environmental conditions.pdf

93777970 https://www.sysrev.com/p/104267/article/93777970 isacadavid9 Woody Perenne Psidium guajava, Passiflora edulis, Carica papaya, Eriobotrya japonica Fruit||| Flower Epyphitic South America Field None Bacteria||| Archea||| Fungi none Yes None Wash the tissue In-house method||| Qiagen DNeasy Blood & Tissue Kit Ion Torrent Sequencing Kraken||| R packages NCBI None read filtering None NO Plant species, different ripening stages, None 2022 largedifferencesinmicrobialcommunity composition occurred between the different tropical fruit sample Genome Microbiomes Associated With the Surfaces of Northern Argentinian Fruits Show a Wide Species Diversity.pdf

93777971 https://www.sysrev.com/p/104267/article/93777971 isacadavid9 Herbaceous Annual Phaseolus vulgaris, Raphanus sativus Seed||| Flower All|||Epyphitic Europe Field None Bacteria 16S rDNA V4 NO None Grind the tissue||| Wash the tissue Others Illumina Sequencing R packages SILVA database None Microorganism specific primers None NO Plant development stage None 2022 changes in the RA of some specific ASVs in seeds that are correlated with abnormal seedlings ASVs Single Seed Microbiota: Assembly and Transmission from Parent Plant to Seedling.pdf

93777972 https://www.sysrev.com/p/104267/article/93777972 erika_frydrych Herbaceous Perenne Polygonum hydropiper|||Polygonum lapathifolium L Leaf||| Stem|||Root Endophitic Asia Other None Fungi none NO ITS Grind the tissue FastDNA Spin Kit for Soil Illumina Sequencing QIIME||| Others||| MOTHUR||| R packages Ribosomal Database Project||| UNITE database None Microorganism specific primers FUNGuild YES characteristics of fungi in Polygonum hydropipe L. and Polygonum lapathifolium L.|||and its effect on the content of active ingredients Cytoscape 2022 the community fromCercosporasignificantly interferes with recruiting fungal communities inP. hydropiperand affects the accumulation of secondary metabolites in the host plant OTUs Colonization characteristics of fungi in Polygonum hydropipe L. and Polygonum lapathifolium L. and its effect on the content of active ingredients.pdf

93777975 https://www.sysrev.com/p/104267/article/93777975 erika_frydrych Herbaceous Perenne Leontopodium nanum|||Stipa purpurea Leaf||| Rhizosphere|||Root||| Bulk soil Epyphitic Asia Field None Fungi none NO 18S rRNA Wash the tissue PowerSoil DNA Isolation Kit Illumina Sequencing QIIME||| R packages Others None Others FUNGuild YES two dominant grass species|||different graphical distributions Others 2022 , fungal alpha diversit y on both leaf and root sur faces increased with precipitatio OTUs Different patterns and drivers of fungal communities between phyllosphere and rhizosphere in alpine grasslands.pdf

93777976 https://www.sysrev.com/p/104267/article/93777976 isacadavid9 Herbaceous Perenne Tradescantia zebrina, native trees of atlantic forest Leaf All South America Field Biotic Fungi none NO ITS1 Grind the tissue Neasy Plant Mini Kit Illumina Sequencing QIIME||| R packages UNITE database None None FUNGuild NO plant invaded field, plant non-invaded field None 2022 The invaded sample showed significantly higher richness and uniformity than the non-invaded litter and a difference in the species dominance ASVs ITS-86F and ITS-4R Turenne et al. 1999 Structural and functional changes in the fungal community of plant detritus in an invaded Atlantic Forest.pdf

93777977 https://www.sysrev.com/p/104267/article/93777977 erika_frydrych Herbaceous Annual Rice Leaf||| Stem Endophitic Asia Greenhouse None Bacteria 16S rDNA V3 - V4 NO None Grind the tissue In-house method Ion Torrent Sequencing QIIME Others None None None NO Bacterial community associated with three rice landraces using leaf blade and stem samples. None 2022 The prokaryotic inhabitants in rice plants is pre- dominantly determined by rice plant organs. ASVs Genotype and organ effect on the occupancy of phyllosphere prokaryotes in different rice landraces.pdf

93777978 https://www.sysrev.com/p/104267/article/93777978 erika_frydrych Woody Perenne Fraxinus excelsior Leaf Endophitic Europe Field None Fungi none NO ITS1 Grind the tissue Neasy Plant Mini Kit Illumina Sequencing VSEARCH||| QIIME||| R packages UNITE database None None None NO . None 2023 . OTUs . Fungal succession in decomposing ash leaves colonized by the ash dieback pathogen Hymenoscyphus fraxineus or its harmless relative Hymenoscyphus albidus.pdf

93777979 https://www.sysrev.com/p/104267/article/93777979 fickgustavo Woody||| Herbaceous Perenne Zea mays|||Pinus taeda|||Spartina alternifora Leaf Endophitic North America Field None Bacteria 16S rDNA V5 - V6 NO None Grind the tissue Others Illumina Sequencing QIIME Greengenes database||| Ribosomal Database Project None PNA||| blocking oligonucleotide None NO eficiência dos primers em diferentes regiões|||diferentes plantas None 2020 t, after the removal of plant-affiliated reads, the number of bacterial endo- phytic reads generated by all assays ranged from 53±36 to 2323±1290 per sample OTUs none Vallisneria natans decreased CH4 fluxes in wetlands: Interactions among.pdf

93777980 https://www.sysrev.com/p/104267/article/93777980 isacadavid9 Herbaceous Annual Triticum aestivum Seed All Europe Field None Fungi none NO ITS1 Grind the tissue Neasy Plant Mini Kit Illumina Sequencing MOTHUR||| R packages UNITE database||| Ribosomal Database Project None None None NO Sample location None 2023 All three regions differed in their microbial composition, Different biomarker were found for every region OTUs Environmental Filtering Drives Fungal Phyllosphere Community in Regional Agricultural Landscapes.pdf

93777981 https://www.sysrev.com/p/104267/article/93777981 isacadavid9 Woody Perenne Pinus radiata Leaf Endophitic||| All Oceania Field None Bacteria||| Fungi 16S rDNA V4 - V5 NO ITS Others DNeasy® PowerPlant® Pro KiIllumina Sequencing R packages UNITE database||| Ribosomal Database Project None Microorganism specific primers None NO Plant height, compartment, cardinal direc- tion, and needle age None 2023 microbiome vary acroos plant height, bottom is more diverse than canopy top ASVs What matters most? Assessment of within-canopy factors influencing the needle microbiome of the model conifer, Pinus radiata.pdf

93777982 https://www.sysrev.com/p/104267/article/93777982 erika_frydrych Herbaceous Annual Lactuca sativa Leaf||| Bulk soil Endophitic Asia Farm None Bacteria 16S rDNA V4 - V5 NO None Grind the tissue FastDNA Spin Kit for Soil Illumina Sequencing QIIME||| R packages Ribosomal Database Project||| SILVA database None Others None NO Different lime dosages|||The transmission of ARGs from soil to vegetables is the concern of this study None 2021 lime application decreased the number and abundance of ARGs and slowed down the spread of manure- derived ARGs in the soil-plant system. Lime addition had a negative effect on ARG diversity OTUs Liming mitigates the spread of antibiotic resistance genes in an acid black soil.pdf

93777983 https://www.sysrev.com/p/104267/article/93777983 erika_frydrych Herbaceous Perenne Seven different cultivars of banana Leaf|||Root Endophitic North America Farm None Fungi none Yes None Wash the tissue Qiagen DNeasy Blood & Tissue Kit Illumina Sequencing PEAR||| Others||| R packages NCBI||| Others None read filtering None NO 7 different cultivars and species of banana None 2023 Metapangenomics revealed key taxa and protypeotective functions that appeared to be driven by genotipe OTUs None Metapangenomics of wild and cultivated banana microbiome reveals a plethora of host-associated protective functions.pdf

93777984 https://www.sysrev.com/p/104267/article/93777984 erika_frydrych Herbaceous Perenne Guarianthe skinneri Leaf||| other|||Root Endophitic North America Garden Biotic Fungi none NO ITS Grind the tissue Others Illumina Sequencing QIIME Others None Microorganism specific primers FUNGuild NO asymptomatic and symptomati

conditions None 2023 endophytic fungi fulfill a wide range of functions in epiphytic orchids, with the potential for a range of applications. ASVs ITS5 and ITS4 (Schoch et al. 201 Community Richness and Diversity of Endophytic Fungi Associated with the Orchid Guarianthe skinneri Infested with "Black Blotch" in the Soconusco Region, Chiapas, Mexico.pdf

93777985 https://www.sysrev.com/p/104267/article/93777985 erika_frydrych Woody Perenne Ulmus glabra Leaf|||Root Endophitic Europe Field Biotic Fungi none NO ITS2 Grind the tissue In-house method PacBio sequencing Others||| R packages Others None Microorganism specific primers None NO whether and how the diversity and composition of fungal communities change in leaves and roots|||Healthy-Looking and Diseased tree None 2022 The detected richness of fungal taxa was higher in samples collected from healthy-looking trees than from diseased ones OTUs Fungal Communities in Leaves and Roots of Healthy-Looking and Diseased Ulmus glabra.pdf

93777986 https://www.sysrev.com/p/104267/article/93777986 isacadavid9 Herbaceous Annual Solanum Lycopersicum, Fragaria x ananassa Leaf Epyphitic Europe Greenhouse None Bacteria 16S rDNA V4 NO None Wash the tissue QIAmp Powerfecal DNA Kit Illumina Sequencing R packages Others None PNA None NO Plant specie, sample location, sample time None 2022 microbiome of both crops was low in diversity and abundance and varied considerably over time and space ASVs The Greenhouse Phyllosphere Microbiome and Associations with Introduced Bumblebees and Predatory Mites.pdf

93777987 https://www.sysrev.com/p/104267/article/93777987 fickgustavo Woody Perenne Casuarina equisetifolia Seed Epiphytic, Endophytic Asia Field None Bacteria||| Fungi 16S rDNA V3 - V4 NO ITS Wash the tissue|||Grind the tissue Others Illumina Sequencing MOTHUR RDP None read filtering None NO Allelopathic effects positive or negative None 2022 the fermentation broth of fungal generaMycosphaerellasp. andPestalotiopsis sp., and bacterial generaBacillus amyloliquefaciens,Burkholderia- Paraburkholderia, andPantoea ananatishad the strongest allelopathic effect onC. equisetifolia seeds. OTUs Is allelochemical synthesis in Casuarina equisetifolia plantation related to litter microorganisms?.pdf

93777988 https://www.sysrev.com/p/104267/article/93777988 erika_frydrych Woody Perenne Populus nigra|||P. simonii Leaf Epyphitic Asia Field None Fungi|||Bacteria 16S rDNA V3 - V4 NO ITS1 Grind the tissue FastDNA Spin Kit for Soil Illumina Sequencing R packages Others None read filtering None NO poplar hybrid offspring and their parents None 2022 Differentiation of phyllosphere bacterial and fungal communities between parents and hybrid offspring. OTUs tudy on the differences of phyllosphere microorganisms between poplar hybrid offspring and their parents.pdf

93777989 https://www.sysrev.com/p/104267/article/93777989 fickgustavo Herbaceous Annual Arabidopsis thaliana Leaf Epiphitic North America Greenhouse Chemical Bacteria functional genes||| 16S rDNA V4 NO None Wash the tissue Powersoil Illumina Sequencing Qiime Greengenes None none None NO . None 2023 A aplicação de 187R teve pouco impacto na composição e função dos componentes da comunidade microbiana comparado ao antibiótico estreptomicina OTUs . Genetic and Environmental Investigation of a Novel.pdf

93777990 https://www.sysrev.com/p/104267/article/93777990 erika_frydrych Woody Perenne 20 tree genera Leaf||| Bulk soil Endophitic Asia Field None Fungi none NO ITS1 Grind the tissue Others||| FastDNA Spin Kit for Soil Illumina Sequencing QIIME UNITE database None None FUNGuild YES between aboveground and belowground habitats Modular 2023 rovide novel insights into cross-kingdom (plant-fungus) species co-occurrence at large spatial sca OTUs Plant and fungal species interactions differ between aboveground and belowground habitats in mountain forests of eastern China.pdf

93777991 https://www.sysrev.com/p/104267/article/93777991 erika_frydrych Woody Perenne 55 species of woody plants Leaf Endophitic Asia Field None Fungi none NO ITS1 Grind the tissue FastDNA Spin Kit for Soil||| Others Illumina Sequencing QIIME UNITE database None Others FUNGuild YES differences in topological properties between aboveground and belowground Modular 2022 Community shifts of trees due to climate change or human activities will impair aboveground and belowground forest fungal diversity in different ways OTUs Scale-Dependent Effects of Growth Stage and Elevational Gradient on Rice Phyllosphere Bacterial and Fungal Microbial Patterns in the Terrace Field.pdf

93777992 https://www.sysrev.com/p/104267/article/93777992 erika_frydrych Herbaceous Perenne Oryza officinalis Root||| Stem||| Leaf Endophitic Asia Greenhouse None Bacteria 16S rDNA V5 - V7 NO None Grind the tissue Others Illumina Sequencing QIIME||| R packages Others None read filtering PICRUSt YES 3 different tissues None 2023 Endophytic bacterial microbiomes are tissue-specific ASVs Endophytic bacterial communities in wild rice (Oryza officinalis) and their plant growth-promoting effects on perennial rice.pdf

93777993 https://www.sysrev.com/p/104267/article/93777993 erika_frydrych Herbaceous Perenne Prunus laurocerasus Leaf Endophitic Europe Field None Bacteria||| Archea 16S rDNA V4 NO None Grind the tissue PowerSoil DNA Isolation Kit Illumina Sequencing QIIME||| R packages Ribosomal Database Project None read filtering None NO Temporal variation None 2022 We identified biomarker taxa for late winter, mid spring, and late spring collection dates. This study is the first one to report on the diversity and composition of bacterial endophytes in the leaves of cherry laurel and its shifts across the dormancy-to-vegetative seasonal transition. ASVs Changes of Endophytic Bacterial Community in Mature Leaves of Prunus laurocerasus L. during the Seasonal Transition from Winter Dormancy to Vegetative Growth.pdf

93777994 https://www.sysrev.com/p/104267/article/93777994 erika_frydrych Herbaceous Perenne Mentha longifolia Leaf|||Root Endophitic Asia Field Salt Bacteria 16S rDNA V3 - V4 NO None Grind the tissue Zymobiomics DNA kit Illumina Sequencing QIIME||| R packages None None None NO two different types of irrigation|||sampling location None 2022 The dynamics in the number, type and distribution of the bacterial endophytic population between the two areas varies in response to the type of stress to which the plant is exposed OTUs influence of Irrigation Water on the Diversity and Distribution.pdf

93777995 https://www.sysrev.com/p/104267/article/93777995 erika_frydrych Woody Annual male Idesia polycarpa Leaf Endophitic Asia Field None Bacteria||| Fungi 16S rDNA V3 - V4 NO ITS1 Grind the tissue E.Z.N.A. Soil DNA Kit Illumina Sequencing QIIME Others None None None NO differences in bacterial diversity between male and female;|||the richness of the fungi of male plants at the flowering and fruit maturity stages None 2022 This study revealed the changes in substances and microorganisms in the leaves of male and female plants in their reproductive stages OTUs Diversity Analysis of Leaf Nutrient Endophytes and Metabolites in Dioecious Idesia polycarpa Maxim Leaves during Reproductive Stages.pdf

93777996 https://www.sysrev.com/p/104267/article/93777996 erika_frydrych Woody Perenne Cordia dodecandra Rhizosphere||| Leaf Endophitic North America Garden|||Field None Bacteria||| Fungi 16S rDNA V1 - V2 NO ITS1 Grind the tissue Zymobiomics DNA kit Illumina Sequencing QIIME||| R packages SILVA database||| UNITE database None read filtering None NO we evaluated the microbiota's alpha and beta diversity per compartment and per population None 2022 y. The alpha diversity of bacteria and fungi was highly variable among samples and was similar among compartments and populations OTUs ITS1F and ITS2aR (Gardes, M et al 1993; White, T. et al 1990) Hidden Tenants: Microbiota of the Rhizosphere and Phyllosphere of Cordia dodecandra Trees in Mayan Forests and Homegardens.pdf

93777997 https://www.sysrev.com/p/104267/article/93777997 isacadavid9 Herbaceous Annual Zea mays, Helianthus annuus Leaf|||Root||| Stem Endophitic Africa Field None Bacteria 16S rDNA V3 - V4 NO None Others Others Illumina Sequencing Others||| R packages Greengenes database None None None NO Incubation time, Plant tissue, culture medium None 2023 differential in vitro culturability of bacterial communities in response to cross cultivation on homologous/heterologous culture media OTUs Cross Cultivation on HomologousHeterologous Plant-Based Culture Media Empowers Host-Specific and Real Time In Vitro Signature of Plant Microbiota.pdf

93777998 https://www.sysrev.com/p/104267/article/93777998 erika_frydrych Herbaceous Annual||| Perenne Aspilia grazielae Branches|||Root||| Leaf Endophitic South America Field Nutrient Fungi Other NO ITS Grind the tissue In-house method Illumina Sequencing Others NCBI_fungi ITS database None read filtering None NO tissue (root and leaves) None 2023 owever, the abundance and wealth of endophytic fungal communities inA. grazielaefrom RCA were evidence that could explain their high resilience to environmental disturbances and the source-sink dynamics of fungal propagu OTUs ITS4 (White, T.J 1990) Impact of Iron Mining Activity on the Endophytic Fungal Community of Aspilia grazielae.pdf

93777999 https://www.sysrev.com/p/104267/article/93777999 erika_frydrych Woody Perenne Quercus aliena Root||| Stem||| Leaf Endophitic Asia Farm Biotic Bacteria||| Fungi 16S rDNA V3 - V4 NO 18S rRNA Wash the tissue In-house method Illumina Sequencing QIIME Ribosomal Database Project None read filtering None NO Effects ofHericium erinaceus Hedgehog mushroom None 2022 The endophytic microbial community structure and dominant species varied in Q. aliena mycorrhized with H. erinaceus OTUs Effects ofHericium erinaceus Hedgehog mushroomon the endophytic microbial community of the host plant.pdf

93778000 https://www.sysrev.com/p/104267/article/93778000 erika_frydrych Herbaceous Perenne Epimedium koreanum Leaf||| Rhizosphere Endophitic Asia Greenhouse None Fungi none NO 16S rRNA Wash the tissue E.Z.N.A. Soil DNA Kit Illumina Sequencing PEAR||| R packages||| MOTHUR Ribosomal Database Project||| UNITE database None Microorganism specific primers

None NO tissue comparison|||growth stage None 2022 The relative abundance of soil fungi in the rhizosphere stage was higher than that of leaf endophytic fungi in the early growth stage. OTUs Diversity analysis of leaf endophytic fungi and rhizosphere soil fungi of Korean Epimedium at different growth stages.pdf

93778001 https://www.sysrev.com/p/104267/article/93778001 isacadavid9 Herbaceous Perenne Saccharum officinarum, S. barberi, S. sinense S. spontaneum, S. robustum, Root||| Stem||| Leaf Endophitic Asia Farm None Bacteria Other NO None Grind the tissue QIAamp DNA Stool Mini Kit Illumina Sequencing MOTHUR||| R packages Ribosomal Database Project||| SILVA database||| UNITE database None Microorganism specific primers None NO plant species, plant tissues None 2022 bacterial microbiomes associated with root tissues differed significantly from stem and leaf tissues of sugarcane, OTUs Unraveling Nitrogen Fixing Potential of Endophytic Diazotrophs of Different Saccharum Species for Sustainable Sugarcane Growth.pdf

93778002 https://www.sysrev.com/p/104267/article/93778002 isacadavid9 Herbaceous Annual Raphanus sativus Rhizosphere||| Leaf|||Root Endophitic Asia Greenhouse|||Field None Bacteria 16S rDNA V5 - V6 NO None Wash the tissue FastDNA Spin Kit for Soil Illumina Sequencing MOTHUR||| QIIME SILVA database||| Ribosomal Database Project None Microorganism specific primers None YES Growth contidions, tissue compartments Others 2022 bacterial richness and diversity values of rhizosphere bacteria were higher than those of endophytes in different compartments OTUs Bacterial Communities in the Endophyte and Rhizosphere of White Radish (Raphanus sativus) in Different Compartments and Growth Conditions.pdf

93778003 https://www.sysrev.com/p/104267/article/93778003 isacadavid9 Woody Perenne Hevea brasiliensis Leaf Epyphitic||| Endophitic Asia Field None Bacteria||| Fungi 16S rDNA V4 NO ITS1 Grind the tissue FastDNA Spin Kit for Soil Illumina Sequencing QIIME||| R packages Ribosomal Database Project None None None NO seasons, geographical location, plant compartment None 2022 fungal com- munities showed a geographical pattern, Significant differences of phyllosphere bacterial communities were detected in plant compartments, Most of the differences in taxa com- position came from Firmicutesspp OTUs hyllosphere fungal communities of rubber trees exhibited biogeographical patterns, but not bacteria.pdf

93778004 https://www.sysrev.com/p/104267/article/93778004 isacadavid9 Woody Perenne Vitis vinifera Leaf Epyphitic||| All Europe Greenhouse Nutrient Fungi none NO ITS1 Grind the tissue||| Wash the tissue Others Illumina Sequencing R packages UNITE database None Microorganism specific primers None NO sample processing (grind or wash), sampling time point, nutrient (sulphur and copper) treatment, None 2022 leaf wash samples were shown to be superior to leaf disks in terms of diversity and ASV number, if endophytes do not have to be included in the analysis ASVs Evaluation of Different Phyllosphere Sample Types for ParallelMetabarcoding of Fungi and Oomycetes inVitis vinifera.pdf

93778005 https://www.sysrev.com/p/104267/article/93778005 isacadavid9 Woody Perenne Vitis amurensi Stem||| Leaf||| Seed||| Fruit Endophitic Asia Field None Fungi none NO ITS1 Grind the tissue Zymobiomics DNA kit Illumina Sequencing QIIME||| R packages UNITE database None None None NO Plant organs, environmental conditions None 2022 The highest number of fungal isolates and sequences were detected in the grape leaves, t lower temperatures and increased precipitation favored the number and diversity of endophytic fungi OTUs The Diversity of Fungal Endophytes from Wild Grape Vitis amurensis Rupr.pdf

93778006 https://www.sysrev.com/p/104267/article/93778006 isacadavid9 Woody Perenne Sophora alopecuroides Root||| Stem||| Leaf||| Seed Endophitic Asia Field None Fungi none NO ITS1 Grind the tissue FastDNA Spin Kit for Soil Illumina Sequencing MOTHUR UNITE database None None FUNGuild NO Plant tissue None 2022 s. The roots had the highest fungal richness and diversity, while the stems had the highest evenness and pedigree diversity, The organ was the main factor affecting the community structure OTUs The Endophytic Fungi Diversity, Community Structure, and Ecological Function Prediction of Sophora alopecuroides in Ningxia, China.pdf

93778007 https://www.sysrev.com/p/104267/article/93778007 isacadavid9 Woody Perenne Paulownia elongata×fortunei Leaf Endophitic Europe Farm None Bacteria 16S rDNA V5 - V7 NO None Grind the tissue FastDNA Spin Kit for Soil Illumina Sequencing Others Ribosomal Database Project None Microorganism specific primers||| read filtering None NO Season, location None 2022 A core microbiome could be found in leaf-associated endophytic communities in trees growing in different location, ASVs Microbial Community, Metabolic Potential and Seasonality of Endosphere Microbiota Associated with Leaves of the Bioenergy Tree Paulownia elongata × fortunei.pdf

93778008 https://www.sysrev.com/p/104267/article/93778008 isacadavid9 Herbaceous Perenne Asplenium delavayi Leaf Endophitic Asia Field||| Garden None Bacteria 16S rDNA V5 - V7 NO None Grind the tissue E.Z.N.A. Soil DNA Kit Illumina Sequencing MOTHUR SILVA database||| Ribosomal Database Project None Microorganism specific primers None NO plant

accesions, localization, cultivation conditions None 2022 bacterial communty composition of fern OTUs shared among accesions are described Bacterial Microbiome in the Phyllo-Endosphere of Highly Specialized Rock Spleenwort.pdf

93778009 https://www.sysrev.com/p/104267/article/93778009 isacadavid9 Woody Perenne Olea europaea Leaf Endophitic Europe Greenhouse Salt Bacteria 16S rDNA V3 - V4 NO None Grind the tissue In-house method Illumina Sequencing QIIME SILVA database None PNA None NO Plant genotype, None 2022 Different salt concentrations affected the leaf endophytic bacterial composition, the enrichment of a peculiar endophytic community could play a significant role in the ability of olive genotypes to withstand salt stress Salt stress in olive tree shapes resident endophytic microbiota.pdf

93778010 https://www.sysrev.com/p/104267/article/93778010 isacadavid9 Woody Perenne Citrus sinensis x Poncirus trifoliata L.Root||| Rhizosphere||| Bulk soil||| Flower||| Leaf Epyphitic||| Endophitic North America Farm None Bacteria||| Fungi 16S rDNA V4 NO ITS Grind the tissue||| Wash the tissue Zymobiomics DNA kit Illumina Sequencing Others SILVA database||| UNITE database None PNA||| read filtering None NO Plant tissue, tissue compartment None 2023 trus microbiome is composed of core taxonomic groups that are mainly of soil origin and that can systemically colonize trees, There is also evidence of a microbial niche compartmentalization ASVs Microbiome diversity, composition and assembly in a California citrus orchard.pdf

93778011 https://www.sysrev.com/p/104267/article/93778011 isacadavid9 Herbaceous Perenne Sinningia magnifica, Sinningia schiffneri, Sinningia speciosa Leaf Endophitic South America Greenhouse None Bacteria||| Fungi 16S rDNA V5 - V7 NO ITS Grind the tissue Zymobiomics DNA kit Illumina Sequencing QIIME SILVA database||| UNITE database None Microorganism specific primers||| read filtering None NO Plant species, year of collection None 2023 Diversity indices confirm richness in the endophytic communities of bacteria and fungi associated with the leaf blades of Sinningia, Comparing the three years of study, the richness of the genera, over time, was decreasing as a effect of environmental change from field to greenhouse ASVs Diversity of bacterial and fungal endophytic communities presents in the.pdf

93778012 https://www.sysrev.com/p/104267/article/93778012 isacadavid9 Woody Perenne Prunus dulcis Branches Endophitic Europe Farm Biotic Bacteria||| Fungi 16S rDNA V5 - V6 NO ITS2 Grind the tissue In-house method Illumina Sequencing Others SILVA database||| UNITE database None None None YES Xylella fastiodiosa infected plants, healthy plants Cytoscape 2022 Bacteria community was affected by pathogen infection, but not fungal community, Xylella fastidiosa Infection Reshapes Microbial Composition and Network Associations in the Xylem of Almond Trees.pdf

93778013 https://www.sysrev.com/p/104267/article/93778013 isacadavid9 Herbaceous Perenne Spartina alterniflora Leaf Epyphitic Asia Field None Bacteria 16S rDNA V4 - V5 NO None Wash the tissue FastDNA Spin Kit for Soil Illumina Sequencing QIIME SILVA database None None None NO Seasons, leaf sample type None 2022 bacterial community diversity and functional guilds varied greatly with variations in leaf locations and seasons Discovering the Characteristics of Community Structures and Functional Properties of Epiphytic Bacteria on Spartina alterniflora in the Coastal Salt Marsh Area.pdf

93778014 https://www.sysrev.com/p/104267/article/93778014 isacadavid9 Herbaceous Perenne Nicotiana tabacum Leaf Epyphitic||| Endophitic Asia Field Biotic, Chemical Bacteria||| Fungi 16S rDNA V4 NO ITS1 Others FastDNA Spin Kit for Soil Illumina Sequencing QIIME SILVA database||| UNITE database None None None YES before and after fungicide application Others 2022 spraying, the fungal community diversity was significantly reduced in symptomatic leaves. The bacterial community diversity did not change significantly. Variations in leaf phyllosphere microbial communities and development of tobacco brown spot before and after fungicide application.pdf

93778015 https://www.sysrev.com/p/104267/article/93778015 isacadavid9 Herbaceous Perenne Coptis chinensis Root||| Leaf||| Rhizosphere Endophitic Asia Field Biotic Bacteria||| Fungi 16S rDNA V3 - V4 NO 18S rRNA Grind the tissue PowerSoil DNA Isolation Kit Illumina Sequencing QIIME Greengenes database||| UNITE database None None None NO infected plant, healthy plant None 2023 nfection with root rot would destroy the ecological balance of the microbiomes in the rhizosphere soil, rhizome and leaf sample Coptischinensis Franch root rot infection disrupts microecological balance of rhizosphere soil and endophytic microbiomes.pdf

93778016 https://www.sysrev.com/p/104267/article/93778016 isacadavid9 Herbaceous Perenne Dicoma anomala Leaf|||Root Endophitic Africa Field None Bacteria 16S rDNA V3 - V4 NO None Grind the tissue In-house method Illumina Sequencing MOTHUR SILVA database None read filtering None NO Seasons, Geography, plant tissue None 2022 Some

endophytic bacteria were found to be tis- sue specific, Some endophytic bacteria were found to be tis- sue specific, diversity differed among seasons Data on metagenomic profiles of bacterial endophyte communities associated with Dicoma anomala.pdf

93778017 https://www.sysrev.com/p/104267/article/93778017 isacadavid9 Herbaceous Perenne Achnatherum inebrians Leaf Epyphitic||| Endophitic Asia Field Biotic Bacteria||| Fungi 16S rDNA V3 - V4 NO ITS1 Wash the tissue|||Grind the tissue Others Illumina Sequencing MOTHUR||| QIIME||| USEARCH SILVA database||| UNITE database None None None NO Epichloë-infected, no-infected plants None 2022 Epichloë altered the composition and diversity of phyllosphere microbial communities, There were correlations between metabolites and microbial phyla The effect of Epichloe¨ endophyte on phyllosphere.pdf

93778018 https://www.sysrev.com/p/104267/article/93778018 isacadavid9 Herbaceous Annual Lactuca sativa Leaf Epyphitic Africa Greenhouse Biotic Bacteria 16S rDNA V3 - V4 NO None Wash the tissue Zymobiomics DNA kit Illumina Sequencing MOTHUR SILVA database||| NCBI None read filtering None NO Presence or absence of Bacillus probiotics None 2022 e epiphytic bacterial community structure can be modulated by the addition of a commercial probiotic Determination of Phylloplane Associated Bacteria of Lettuce from a Small-Scale Aquaponic System via 16S rRNA Gene Amplicon Sequence Analysis.pdf

93778019 https://www.sysrev.com/p/104267/article/93778019 isacadavid9 Woody Perenne Vitis vinifera Fruit||| Branches||| other Epyphitic Europe Farm None Bacteria||| Fungi 16S rDNA V4 NO ITS2 Wash the tissue Others Ion Torrent Sequencing Others SILVA database None None None NO Plant genotype, Phenological stage None 2022 Microbiome structure was different among the different tissues/developmental stages and among the grapevine genotypes and cultivar Comparative Analysis of Grapevine Epiphytic Microbiomes.pdf

93778020 https://www.sysrev.com/p/104267/article/93778020 isacadavid9 Herbaceous Perenne Colobanthus quitensis Leaf Endophitic Antartica Field heat Bacteria||| Fungi 16S rDNA V5 - V7 NO ITS2 Grind the tissue Nucleospin Plant II ki Illumina Sequencing Others SILVA database||| UNITE database None Microorganism specific primers None NO Collection site, Temperature None 2022 the taxonomic structure of microbiome was shape by the collection site and simulation of global warming Simulated global warming affects endophytic bacterial and fungal communities of Antarctic pearlwort leaves and some bacterial isolates support plant growth at low temperatures.pdf

93778021 https://www.sysrev.com/p/104267/article/93778021 isacadavid9 Herbaceous Annual Vigna unguiculata Leaf|||Root||| Stem Endophitic Africa Farm None Fungi none NO ITS2 Grind the tissue Nucleospin Plant II ki Illumina Sequencing QIIME UNITE database None None None NO Tissue None 2022 Mycobiome differed among tissues Characterization of the Endophytic Mycobiome in Cowpea (Vigna unguiculata) from a Single Location Using Illumina Sequencing.pdf

93778022 https://www.sysrev.com/p/104267/article/93778022 erika_frydrych Woody Perenne Populus tremula Leaf Endophitic Europe Garden None Fungi none NO ITS2||| ITS Grind the tissue E.Z.N.A. Plant DNA Kit||| Others Illumina Sequencing Others Others None Microorganism specific primers None NO Two extraction kits were compared None 2022 The optimized protocol allowed us to successfully prepare an amplicon library in order to subject the intended 380 environmental samples to HTS. ASVs Optimization of Protocol for Construction of Fungal ITS Amplicon Library for High-Throughput Illumina Sequencing to Study the Mycobiome of Aspen Leaves.pdf

93778023 https://www.sysrev.com/p/104267/article/93778023 isacadavid9 Woody Perenne Coffea canephora Fruit|||Root||| Rhizosphere||| Bulk soil All South America Farm None Bacteria 16S rDNA V4 NO None Grind the tissue PowerSoil DNA Isolation Kit Illumina Sequencing MOTHUR SILVA database None None None NO Coffe variety, fermentation None 2023 e microbial community may vary depending on the coffee variety and play an essential role in fermentation Characterization of the Rhizosphere Bacterial Microbiome and Coffee Bean Fermentation in the Castillo-Tambo and Bourbon Varieties in the Popayán-Colombia Plateau.pdf

96830542 https://www.sysrev.com/p/104267/article/96830542 isacadavid9 Woody Perenne Cornus florida Leaf Endophitic North America Field None Fungi none Yes ITS Grind the tissue Neasy Plant Mini Kit Illumina Sequencing QIIME Ribosomal Database Project||| UNITE database None read filtering None NO Tissue with or without antracnose None 2024 None ASVs None Metagenomic study reveals hidden relationships among fungal diversity, variation of plant disease, and genetic distance in Cornus florida (Cornaceae).pdf Pais, Andrew; Ristaino, Jean; Whetten, Ross; Xiang, Jenny; Gonzalez-Rodriguez, Antonio; Cestaro, Alessandro; Zitlalpopoca Hernandez, Guadalupe

96830543 https://www.sysrev.com/p/104267/article/96830543 isacadavid9 Herbaceous Perenne Rheum spiciforme, Eriophyton wallichii, Rhizosphere|||Root||| Leaf Endophitic Asia Field None Fungi none NO ITS2 Grind the tissue PowerSoil DNA Isolation Kit Illumina Sequencing R packages UNITE database||| Ribosomal Database Project None None None YES Plant species, plant compartment Gephi 2024 variation in diversity and composition of the fungal community was predominantly shaped by plant compartment niche rather than plant species. OTUs fITS7 and ITS4 Plant compartment niche is more important in structuring the fungal community associated with alpine herbs in the subnival belt of the Qiangyong glacier than plant species.pdf

96830545 https://www.sysrev.com/p/104267/article/96830545 isacadavid9 Herbaceous Annual Oryza sativa Leaf All Asia Farm None Bacteria 16S rDNA V4||| 16S rDNA NO None Grind the tissue In-house method Illumina Sequencing QIIME SILVA database None None None NO . None 2024 . OTUs None Uncovering microbiomes of the rice phyllosphere using long-read metagenomic sequencing.pdf Masuda, Sachiko; Gan, Pamela; Kiguchi, Yuya; Anda, Mizue; Sasaki, Kazuhiro; Shibata, Arisa; Iwasaki, Wataru; Suda, Wataru; Shirasu, Ken

96830546 https://www.sysrev.com/p/104267/article/96830546 isacadavid9 Herbaceous Perenne Stipa capillata,Agropyron cristatum,Leymus chinensis,Cleisto- genes squarrosa,Koeleria macrantha,Neotrinia splendens,Po t e n - tilla acaulis,Potentilla bifurca,Potentilla tanacetifolia,Potentilla betonicifolia,Potentilla anserina,Potentilla multifida,Artemisia frigida,Aster altaicus,Medicago ruthenica,Astragalus galactites, Carex korshinskyi,Allium bidentatum, Stellera chamaejasme Leaf All Asia Field None Bacteria, Fungi 16S rDNA V5 - V7 NO ITS2 Grind the tissue FastDNA Spin Kit for Soil Illumina Sequencing Others SILVA database||| UNITE database None read filtering None YES Plant species Cytoscape 2024 Plant identity exerted significant impacts onα-diversities of both bacterial and fungal communities. OTUs ITS3F/ITS4R (White et al. 1990) Host Identity Determines the Bacterial and Fungal Community and Network Structures in the Phyllosphere of Plant Species in a Temperate Steppe.pdf Guo, Chunyan; Yang, An; Zhang, Wen-Hao

96830547 https://www.sysrev.com/p/104267/article/96830547 isacadavid9 Woody Perenne Camellia oleifera Leaf Epyphitic||| Endophitic Asia Field None Bacteria 16S rDNA V4 - V5 NO None Wash the tissue PowerSoil DNA Isolation Kit Illumina Sequencing Others SILVA database None None None NO Cultivar, sample location, tissue compartment None 2024 diversity and species richness of endophytic microbial community in leaves were significantly higher than those of microbial community in the epiphytic. OTUs None Epiphytic and endophytic bacteria on Camellia oleifera phyllosphere: exploring region and cultivar effect.pdf Chen, Xiaolin; Li, Lili; He, Yuanhao

96830548 https://www.sysrev.com/p/104267/article/96830548 isacadavid9 Woody Perenne Vitis vinifera Fruit Epyphitic Europe Farm None Fungi none NO ITS2 Wash the tissue Others Illumina Sequencing QIIME||| R packages UNITE database None None None NO cultivars None 2024 None ASVs s 2024F and 2409R (White et al., 1990; Zhang et al., 201 Diversity of fungal communities on Cabernet and Aglianico grapes from vineyards located in Southern Italy.pdf Tofalo, Rosanna; Capece, Angela; Fragasso, Mariagiovanna; Succi, Mariantonietta; Pannella, Gianfranco; Iorizzo, Massimo; Bagnoli, Diletta; Vergalito, Franca; Testa, Bruno; Tremonte, Patrizio; Letizia, Francesco; Albanese, Gianluca; Lombardi, Silvia Jane.; Coppola, Raffaele

96830550 https://www.sysrev.com/p/104267/article/96830550 isacadavid9 Herbaceous Perenne Arachis hypogaea) Seed Endophitic Asia Greenhouse Biotic Bacteria 16S rDNA V3 - V4 NO None Grind the tissue Others Illumina Sequencing Others Others None None None NO with and without syncom None 2024 . OTUs None Seed-borne bacterial synthetic community resists seed pathogenic fungi and promotes plant growth.pdf Luo, De-Lin; Huang, Shi-Yi; Ma, Chen-Y U; Zhang, Xiang-Y U; Sun, Kai; Zhang, Wei; Dai, Chuan-Chao

96830552 https://www.sysrev.com/p/104267/article/96830552 isacadavid9 Herbaceous Annual Nicotiana tabacum Leaf All Asia Field Chemicals Bacteria||| Fungi 16S rDNA V4 NO ITS1 Grind the tissue In-house method Illumina Sequencing MOTHUR||| QIIME||| R packages UNITE database||| SILVA database None None PICRUSt NO fungicide treatment effect, n environmental facto None 2024 none OTUs ers ITS5- 1F-F and ITS1-1F-R (Wang et al., 2020) Response of microbial communities in the tobacco phyllosphere under the stress of validamycin.pdf Guo, Moyan; Hu, Jingrong; Jiang, Chaoying; Zhang, Yi; Wang, Hancheng; Xinghong Zhang; Hsiang, Tom; Shi, Caihua; Wang, Qing; Wang, Feng; Hossain, Md Motaher.; Sheikh, Bangabandhu; Rahman, Mujibur; Solanki, Manoj Kumar.

96830553 https://www.sysrev.com/p/104267/article/96830553 isacadavid9 Herbaceous Annual Triticum aestivum Seed||| Stem All North America Other None Fungi none NO ITS2 Grind the tissue Others Illumina Sequencing Others Others None None None NO . None 2024 . OTUs . Wheat fungal endophyte communities are inseparable from the host and influence plant development.pdf Sharon, Or; Kagan-Trushina, Naomi; Sharon, Amir

96830581 https://www.sysrev.com/p/104267/article/96830581 erika_frydrych Herbaceous Annual Zea mays|||Helianthus annuus Leaf||| Stem|||Root Endophitic Africa Field None Bacteria 16S rDNA V3 - V4 NO None Others Others Illumina Sequencing Others Greengenes database None None None NO . None 2023 . OTUs . Cross

Cultivation on Homologous_Heterologous Plant-Based Culture Media Empowers Host-Specific and Real Time In Vitro Signature of Plant Microbiota.pdf Elsawey, Hend ;.; Nour, Ema H ;.; Elsayed, Tar R ;.; Nemr; Nemr, Rahma A.; Yousse, Hanan H.; Hamza, Mervat A.; Abbas, Mohamed; El-Tahan, Mahmoud; Fayez, Mohamed; Ruppel, Silke; Hegazi, Nabil A.

96830582 https://www.sysrev.com/p/104267/article/96830582 erika_frydrych Woody Annual Vitis vinifera other Epyphitic Europe Field None Bacteria||| Fungi 16S rDNA V4 NO ITS2 Others Nucleospin Plant II ki Ion torrent Others SILVA database None None None NO . None 2022 . OTUs . Comparative Analysis of Grapevine Epiphytic Microbiomes among Different Varieties, Tissues, and Developmental Stages in the Same Terroir.pdf Awad, Murad; Giannopoulos, Georgios; Mylona, Photini V.; Polidoros, Alexios N.

96830583 https://www.sysrev.com/p/104267/article/96830583 erika_frydrych Herbaceous Annual Avena sativa Root||| Leaf Endophitic Asia Field None Fungi none NO ITS Grind the tissue Others Illumina Sequencing Others UNITE database None None FUNGuild NO . None 2023 . OTUs . COMMUNITY STRUCTURE OF ENDOPHYTIC FUNGI IN ROOTS AND LEAVES OF Fagopyrum mill AND Avena sativa IN A CHINESE NORTHERN COLD REGION.pdf Gao, Yamei; Li, Zhiwen; Han, Yiqiang; Han, Z

96830585 https://www.sysrev.com/p/104267/article/96830585 erika_frydrych Woody Perenne Malus domestica Leaf Endophitic Europe Nurseries None Bacteria||| Fungi 16S rDNA V5 - V7 NO ITS1 Others Neasy Plant Mini Kit Illumina Sequencing USEARCH||| R packages Ribosomal Database Project None PNA others NO . None 2023 . OTUs . Relative contribution of season, site, scion and rootstock genotype, and susceptibility to European canker to the variability in bacterial and fungal communities in apple leaf scar tissues.pdf Papp-Rupar, Matevz; Deakin, Greg; Olivieri, Leone; Robinson-Boyer, Louisa; Xu, Xiangming

96830586 https://www.sysrev.com/p/104267/article/96830586 erika_frydrych Herbaceous Perenne tobacco Leaf None Asia Field Metals Bacteria||| Fungi 16S rDNA V4 NO ITS Others In-house method Ion Torrent Sequencing QIIME||| R packages UNITE database||| SILVA database None None PICRUSt||| FUNGuild YES . Cytoscape 2023 . OTUs . Response of microbial communities in the phyllosphere ecosystem of tobacco exposed to the broad-spectrum copper hydroxide.pdf Feng, Ruichao; Wang, Hancheng; Liu, Tingting; Wang, Feng; Cai, Liuti; Chen, Xingjiang; Zhang, Songbai; Sofo, Adriano; Chuanqing, Zhang; Zhang, Chao; Zhang, Feng

96830587 https://www.sysrev.com/p/104267/article/96830587 erika_frydrych Herbaceous Perenne Picrorhiza kurrooa Leaf|||Root Endophitic Asia Field None Bacteria Other NO None Others FastDNA Spin Kit for Soil Illumina Sequencing QIIME Greengenes database None None PICRUSt NO . None 2023 . OTUs . Endomicrobiome of in vitro and natural plants deciphering the endophytes-associated secondary metabolite biosynthesis in Picrorhiza kurrooa, a Himalayan medicinal herb.pdf Tamang, Anish; Swarnkar, Mohit; Kumar, Pawan; Kumar, Dinesh; Shiv, Shanker; Pandey; Hallan, Vipin

96830588 https://www.sysrev.com/p/104267/article/96830588 erika_frydrych Herbaceous|||Woody Annual ive mangrove species Root||| Leaf||| Branches Endophitic Asia Field None Bacteria 16S rDNA V5 - V7 NO None Others Others Illumina Sequencing R packages Others None None None NO . None 2023 . OTUs . Community structures of mangrove endophytic and rhizosphere bacteria in Zhangjiangkou National Mangrove Nature Reserve.pdf Yuan, Zongsheng; Zeng, Zhihao; Liu, Fang

96830589 https://www.sysrev.com/p/104267/article/96830589 erika_frydrych Herbaceous Annual||| Perenne Mirabilis himalaica Root||| Stem||| Leaf Endophitic Asia Other None Bacteria 16S rDNA V5 - V7 NO None Others Others Illumina Sequencing MOTHUR||| USEARCH||| QIIME SILVA database None None PICRUSt NO . None 2023 . OTUs . Endophytic bacterial community structure and diversity of the medicinal plant Mirabilis himalaica from different locations.pdf Zhang, Erhao; Lu, Yazh; Zhao, Rundong; Yin, Xiu; Zhang, Jie; Yu, Benxia; Yao, Min; Liao, Zhihua; Lan, Xiaozhong; Tavora, Caio; Da Costa Rachid, Coelho

96830590 https://www.sysrev.com/p/104267/article/96830590 erika_frydrych Woody||| Herbaceous Perenne Picea abies|||Alnus incana|||Calamagrostis epigejos|||Vaccinium myrtillus|||Nuphar lutea|||Myriophyllum spicatum|||Acorus calamus|||E. fluviatile Root||| Leaf None Europe Field None Fungi none NO ITS Wash the tissue Others PacBio sequencing MOTHUR||| VSEARCH UNITE database None PNA None NO . None 2023 . OTUs . Structure of plant-associated microeukaryotes in roots and leaves of aquatic and terrestrial plants revealed by blocking peptide-nucleic acid (PNA) amplification.pdf Azadnia, Avid; Mikryukov, Vladimir; Anslan, Sten; Hagh-Doust, Niloufar; Rahimlou, Saleh; Tamm, Heidi; Tedersoo, Leho

96830591 https://www.sysrev.com/p/104267/article/96830591 erika_frydrych Herbaceous Perenne Ardisia crenata Leaf|||Root Endophitic Asia Field None Bacteria||| Fungi 16S rDNA V4 NO ITS1 Others Others Illumina Sequencing VSEARCH NCBI_fungi ITS database None None FUNGuild NO . None 2023 . OTUs . Leaf, root, and soil microbiomes of an invasive plant, Ardisia crenata, differ between its native and exotic ranges.pdf Nakamura, Naoto; Toju, Hirokazu; Kitajima, Kaoru; Ruotsalainen, Anna Liisa.

96830592 https://www.sysrev.com/p/104267/article/96830592 erika_frydrych Herbaceous Annual Oryza sativa Seed All Africa Field None Bacteria 16S rDNA V3 - V4 NO None Grind the tissue Neasy Plant Mini Kit Illumina Sequencing QIIME Others None None PICRUSt NO . None 2023 . ASVs . Exploiting the microbiome associated with normal and abnormal sprouting rice (Oryza sativa L.) seed phenotypes through a metabarcoding approach%0A.pdf Nguefack, Julienne; Musonerimana, Samson; Giovanardi, Davide; Dongmo Nanfack, Albert; China, Salvatore La.; Stefani, Emilio

96830593 https://www.sysrev.com/p/104267/article/96830593 erika_frydrych Herbaceous Perenne Phyllostachys edulis|||Bambusa rigida|||Pleioblastus amarus Leaf Endophitic Asia Field None Fungi none NO ITS1 Others Others Illumina Sequencing Others Others None None FUNGuild NO n None 2023 n OTUs m Comparative analysis of endophytic fungal communities in bamboo species Phyllostachys edulis, Bambusa rigida, and Pleioblastus amarus.pdf Yan, Kuan; Zhang, Jian; Cai, Yu; Cao, Guiling; Meng, Lina; Soaud, Salma A.; Heakel, Rania M Y.; Ihtisham, Muhammad; Zhao, Xianming; Wei, Qin; Dai, Tainfei; Abbas, Manzar; El-Sappah, Ahmed H.

96830594 https://www.sysrev.com/p/104267/article/96830594 erika_frydrych Herbaceous Perenne|||Annual Potentilla fruticosa Rhizosphere|||Root||| Leaf Endophitic Asia Field None Bacteria 16S rDNA V5 - V7 NO None Grind the tissue Others Illumina Sequencing USEARCH SILVA database||| Ribosomal Database Project None None None NO n None 2023 n OTUs n Endophytic bacteria in the periglacial plant Potentilla fruticosa var. albicans are influenced by habitat type.pdf Sonam, Wangchen; Liu, Yongqin; Guo, Liangdong

96830595 https://www.sysrev.com/p/104267/article/96830595 erika_frydrych Herbaceous Annual||| Perenne Nicotiana tabacum Leaf None Asia Field Biotic Bacteria||| Fungi 16S rDNA V5 - V7 NO ITS Grind the tissue E.Z.N.A. Soil DNA Kit Illumina Sequencing Others UNITE database||| Ribosomal Database Project||| SILVA database None None None YES n Gephi 2023 n OTUs n Microbial community and chemical composition of cigar tobacco (Nicotiana tabacum L.) leaves altered by tobacco wildfire disease.pdf Si, Hongyang; Cui, Bing; Liu, Fang; Zhao, Mingqin

96830596 https://www.sysrev.com/p/104267/article/96830596 erika_frydrych Herbaceous Perenne Ipomoea batatas Root||| Stem||| Leaf Endophitic Asia Field Biotic Bacteria||| Fungi 16S rDNA V5 - V7 NO ITS Others FastDNA Spin Kit for Soil Illumina Sequencing Others SILVA database||| Ribosomal Database Project None None None NO Scab Disease Caused byElsinoë batatas None 2023 none OTUs none Structure of Endophytes in the Root, Stem, and Leaf Tissues of Sweetpotato and Their Response to Sweetpotato Scab Disease Caused by Elsinoë batatas.pdf Wang, Shixin; Ma, Tingti; Yao, Xiaojian; Yao, Zhufang; Wang, Zhangying; Dong, Zhangyong; Luo, Mei; Huang, Lifei

96830960 https://www.sysrev.com/p/104267/article/96830960 erika_frydrych Herbaceous Perenne Hydrilla verticillata Leaf Epyphitic Asia Greenhouse Chemicals Bacteria 16S rDNA V4 NO None Others Power Biofilm DNA isolation kit Illumina Sequencing QIIME||| R packages Others None None None NO Uso de norfloxacin None 2023 . ASVs . Coupling of submerged macrophytes and epiphytic biofilms reduced methane emissions from wetlands Evidenced by an antibiotic inhibition experiment.pdf Lu, Jianhui; Mu, Xiaoying; Zhang, Songhe; Song, Yingying; Ma, Yu; Luo, Min; Duan, Rufei; Gan, Jay

96830961 https://www.sysrev.com/p/104267/article/96830961 erika_frydrych Herbaceous Annual Vallisneria natans Leaf Epyphitic Asia Other None Bacteria 16S rDNA V4 - V5 NO None Others Power Biofilm DNA isolation kit Illumina Sequencing Others SILVA database||| Ribosomal Database Project None None None NO . None 2023 . OTUs . Vallisneria natans decreased CH4 fluxes in wetlands Interactions among.pdf Yang, Liu; Zhang, Songhe; Lv, Xin; Liu, Yuansi; Guo, Shaozhuang; Hu, Xiuren; Manirakiza, Benjamin; Wang, Aijie

99840726 https://www.sysrev.com/p/104267/article/99840726 erika_frydrych Herbaceous Annual Lactuca sativa Leaf Endophitic South America Field None Bacteria 16S rDNA V3 - V4 NO None Grind the tissue PowerSoil DNA Isolation Kit Illumina Sequencing USEARCH||| VSEARCH||| R packages||| PEAR SILVA database None read filtering None NO . None 2024 . OTUs . Characterization of bacterial diversity in rhizospheric soils, irrigation water, and lettuce crops in municipalities near the Bogotá river, Colombia Echeverry-Gallego, Rodrigo A.; Martínez-Pachón, Diana; Arenas, Nelson Enrique.; Franco, Diego C.; Moncayo-Lasso, Alejandro; Vanegas, Javier

99840727 https://www.sysrev.com/p/104267/article/99840727 erika_frydrych Woody Perenne Rhododendron rubiginosum|||Rhododendron sikangense|||Rhododendron pubicostatum Rhizosphere||| other|||Root||| Leaf Endophitic Asia Field None Fungi none NO ITS Wash the tissue FastDNA Spin Kit for Soil Illumina Sequencing QIIME||| R packages Ribosomal Database Project None read filtering FUNGuild NO . None 2024 . OTUs ITS1F and TS2R Composition and Assembly of the Endophytic Fungal Community of Alpine Rhododendron Hosts Along Elevation Gradients Zhang, Rui; Zhou, Xiong-Li; Yang, Liu; Long, Bo; Shen, Shi-Kang

99840728 https://www.sysrev.com/p/104267/article/99840728 erika_frydrych Woody Perenne Aquilaria sinensis Leaf||| Branches|||Root||| Seed||| Flower||| Trunk Endophitic Asia Field None Bacteria||| Fungi 16S rDNA V5 - V7 NO ITS1||| ITS2 Grind the tissue Others Illumina Sequencing USEARCH||| R packages UNITE database||| SILVA database None None None NO . None 2024 . OTUs . Deciphering the roles of bacterial and fungal communities in the formation and quality of agarwood Fu, Chen-Chen; Huang, Bao-Xing; Wang, Shan-Shan; Song, Yu-Chen; Metok, Dolkar; Tan, Yu-Xiang; Fan, Tai-Ping; Fernie, Alisdair R.; Zargar, Meisam; Wang, Yan; Chen, Mo-Xian; Yu, Liang-Wen; Zhu, Yuan; Xu, Jin-Rong

99840729 https://www.sysrev.com/p/104267/article/99840729 erika_frydrych Woody Annual Theobroma grandiflorum Leaf Endophitic South America Farm None Bacteria Other NO None Grind the tissue In-house method PacBio sequencing Others Others None read filtering None NO . None 2024 . Genome . Diversity and potential functional role of phyllosphere-associated actinomycetota isolated from cupuassu (Theobroma grandiflorum) leaves: implications for ecosystem dynamics and plant defense strategies Pereira De Matos, Jéssica; Fagundes, Dilson; Collart, Martine; Karla Da Silva, Ana; Henriques De Paula, Camila; Ferreira Cordeiro, Isabella; Gracyelle De Carvalho Lemes, Camila; Bianchini Sanchez, Angélica; Cachuite, Lorrana; Rocha, Mendes; Machado, Camila Carrião.; Almeida, Nalv; Alves, Rafael Moyses.; De Abreu, Vinicius A C.; Varani, Alessandro M.; Moreira, Leandro Marcio.

99840731 https://www.sysrev.com/p/104267/article/99840731 erika_frydrych Woody Perenne Anacardium occidentale Leaf||| Flower||| Fruit Epyphitic||| Endophitic Africa Field None Fungi|||Bacteria none NO ITS1||| 28S rRNA Grind the tissue DNeasy® PowerPlant® Pro KiIllumina Sequencing USEARCH||| VSEARCH||| R packages UNITE database None read filtering FUNGuild YES . Others 2024 . OTUs ITS1/ITS4a nd NL1/NL4 Endophytic and epiphytic metabarcoding reveals fungal communities on cashew phyllosphere in Kenya Mukhebi, Dennis Wamalabe.; Colletah, Rhoda; Musangi; Moraa Isoe, Everlyne; Neondo, Johnstone Omukhulu.; Mbinda, Wilton Mwema.

99840732 https://www.sysrev.com/p/104267/article/99840732 erika_frydrych Herbaceous Perenne Polygonum cuspidatum Root||| Leaf||| Stem||| Flower||| Seed Endophitic Asia Field None Bacteria||| Fungi 16S rDNA NO ITS1 Grind the tissue In-house method Illumina Sequencing QIIME||| R packages SILVA database||| UNITE database None read filtering None NO . None 2024 . OTUs . Endophytic Bacteria and Fungi Associated with Polygonum cuspidatum in the Russian Far East Aleynova, Olga A.; Ananev, Alex A.; Nityagovsk, Nikolay N.; Suprun, Andrey R.; Zh, Nursaule; Zhanbyrshina; Beresh, Alina A.; Ogneva, Zlata V.; Tyunin, Alexey P.; Kiselev, Konstantin V.

99840733 https://www.sysrev.com/p/104267/article/99840733 erika_frydrych Herbaceous Annual Oryza eichingeri Root||| Stem||| Leaf Endophitic Asia Greenhouse None Bacteria 16S rDNA NO None Grind the tissue Others Illumina Sequencing Others Others None None others NO . None 2024 . Genome . Endophytic Bacterial Communities in Wild Rice (Oryza eichingeri) and Their Effects on Cultivated Rice Growth Xie, Liang; Li, Jinlu; Xiao, Suqin; Jiang, Hao; Liu, Li; Zhong, Qiaofang; Chen, Ling; Kan, Wang; Yin, Fuyou; Yu, Tengqiong; Zhang, Yun; Wang, Bo; Jiang, Cong; Xing, Jiaxin; Cheng, Zaiquan

99840734 https://www.sysrev.com/p/104267/article/99840734 erika_frydrych Herbaceous Annual Oryza sativa Leaf||| other|||Root Endophitic South America Greenhouse None Bacteria functional genes NO None Grind the tissue Others Roche 454 Sequencing USEARCH||| QIIME Ribosomal Database Project None None None NO . None 2024 . OTUs . Endophytic diazotrophic communities from rice roots are diverse and weakly associated with soil diazotrophic community composition and soil properties Err Ando, Lucía F.; Rariz, Gastón; Ea Martínez-Per Eyra, Andr; Fernández-Scavino, Ana

99840735 https://www.sysrev.com/p/104267/article/99840735 erika_frydrych Woody Perenne Theobroma cacao|||Dipteryx sp.|||Lacmellea panamensis|||Apeiba membranacea|||Heisteria concinna|||Chrysophyllum cainito|||Cordia alliodora Leaf Endophitic Central America Greenhouse Biotic Fungi none NO ITS Grind the tissue DNeasy® PowerPlant® Pro KiIllumina Sequencing VSEARCH UNITE database None None None NO . None 2025 . OTUs ITS1f and ITS4 Evaluating endophyte-rich leaves and leaf functional traits for protection of tropical trees against natural enemies Aponte, Bolívar; Arnold, | A Elizabeth.; Mareli, |; Juliá, Sánchez; Van Bael, Sunshine A.; Rasmann, Sergio; Arnold, A Elizabeth.; Sánchez, Mareli

99840737 https://www.sysrev.com/p/104267/article/99840737 erika_frydrych Woody Annual Quercus ilex Leaf Epyphitic||| Endophitic Europe Greenhouse None Bacteria||| Fungi 16S rDNA V5 - V6 NO ITS1 Grind the tissue FastDNA Spin Kit for Soil Illumina Sequencing R packages SILVA database||| Ribosomal Database Project||| UNITE database None None None NO . None 2024 . ASVs . Microbiomes of urban trees: unveiling contributions to atmospheric pollution mitigation Gandolfi, Isabella; Canedoli, Claudia; Rosatelli, Asia; Covino, Stefano; Cappelletti, David; Sebastiani, Bartolomeo; Tatangelo, Valeria; Corengia, Davide; Pittino, Francesca; Padoa-Schioppa, Emilio; Báez-Matus, Ximena; Hernández, Lisette; Seeger, Michael; Saati-Santamaría, Zaki; García-Fraile, Paula; López-Mondéjar, Rubén; Ambrosini, Roberto; Papacchini, Maddalena; Franzetti, Andrea; Puglia, Anna Maria.; Borin, Sara; Mapelli, Francesca

99840742 https://www.sysrev.com/p/104267/article/99840742 erika_frydrych Woody Perenne Avicennia marina Root||| Leaf||| Bulk soil Endophitic Asia Field Salt Bacteria 16S rDNA V5 - V7||| 16S rDNA NO None Grind the tissue||| Wash the tissue Others Illumina Sequencing QIIME Others None None PICRUSt YES . Gephi 2024 . ASVs . of the Avicennia marina ecosystem for enhancing plant resilience to saline conditions Hirt, Heribert; Saad, Maged M.; Khalaf, Amal; Parween, Sabiha

99840743 https://www.sysrev.com/p/104267/article/99840743 erika_frydrych Woody None urban trees Leaf||| other Epyphitic Asia Other None Bacteria 16S rDNA V3 - V4 NO None Grind the tissue E.Z.N.A. Soil DNA Kit Illumina Sequencing QIIME||| R packages||| VSEARCH SILVA database None None None NO . None 2024 . OTUs||| ASVs . Urban greenspace types and climate factors jointly drive the microbial community structure and co-occurrence network Wang, Huan; Feng, Yilong; Zhang, Qiaoyong; Zou, Min; Li, Ting; Ai, Lijiao; Wang, Haiyang

99840744 https://www.sysrev.com/p/104267/article/99840744 erika_frydrych Woody Perenne Vitis amurensis Leaf Endophitic Europe None Biotic Bacteria 16S rDNA NO None Grind the tissue In-house method Others Others Others None None None NO . None 2024 . Genome . Whole Genome Sequencing of Bacillus velezensis AMR25, an Effective Antagonist Strain against Plant Pathogens Ananev, Alexey A.; Ogneva, Zla V.; Nityagovsky, Nikolay N.; Suprun, Andrey R.; Kiselev, Konstantin V.; Aleynova, Olga A.
