# Supplementary material for: Phyllosphere microbiomes uncovered: Research trends, geographic disparities, and key microbial players
Source: Genet Mol Biol. 2026 Jan 23;49(Suppl 1):e20250083. doi: 10.1590/1678-4685-GMB-2025-0083 (PMC12893196; doi:10.1590/1678-4685-GMB-2025-0083)
Supplement: Table S3 - [file 1415-4757-GMB-49-s1-e20250083-s3.pdf]

Genetics and Molecular Biology
https://doi.org/10.1590/1678-4685-GMB-2025-0083

Supplementary Material to: Phyllosphere microbiomes uncovered: Research trends, geographic disparities, and key microbial players

Table S3. Studied plants in terms of phyllosphere microbiome.

	Plants	Publications #
**Woody**	*Vitis*	19
	Picea	13
	Pinus	10
	Fagus	9
	Populus	8
	Quercus	9
	Citrus	6
	Olea europaea	6
	Prunus	5
	Nepenthes	5
	Coffea	4
	Castanea	3
	Malus domestica	2
	Olea europaea	2
**Herbaceous**	*Oryza sativa*	14
	Triticum	12
	Lactuca	12
	Zea mays	10
	Arabidopsis thaliana	7
	Solanum lycopersicum	7
	Glycine max	5
	Hordeum	5
	Spinacia	5
	Nicotiana	4
	Saccharum	4
	Phaseolus	4
	Brassica	3
	Avena	3
	Sorghum	3

Plants	Publications #
Manihot	2
Vicia	2
Pisum	2
Aloe	1
Cannabis	1
