# Supplementary material for: Phyllosphere microbiomes uncovered: Research trends, geographic disparities, and key microbial players
Source: Genet Mol Biol. 2026 Jan 23;49(Suppl 1):e20250083. doi: 10.1590/1678-4685-GMB-2025-0083 (PMC12893196; doi:10.1590/1678-4685-GMB-2025-0083)
Supplement: Figure S1 - [file 1415-4757-GMB-49-s1-e20250083-s4.pdf]

Genetics and Molecular Biology
https://doi.org/10.1590/1678-4685-GMB-2025-0083

Supplementary Material to: Phyllosphere microbiomes uncovered: Research trends, geographic disparities, and key microbial players

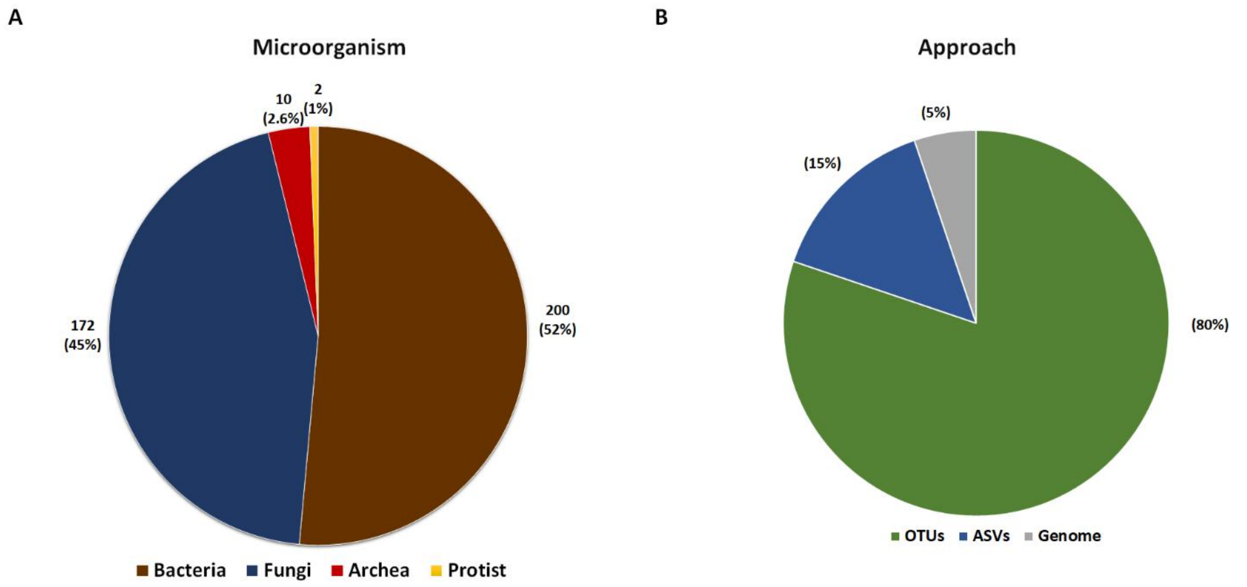

a. Proportion of studies targeting different microbial groups. **b.** Sequencing and analysis approaches applied. Percentages are based on the total number of reviewed studies.

Figure S1 - Distribution of studied microorganisms and analytical approaches in phyllosphere microbiome research.
