# Supplementary material for: Phyllosphere microbiomes uncovered: Research trends, geographic disparities, and key microbial players
Source: Genet Mol Biol. 2026 Jan 23;49(Suppl 1):e20250083. doi: 10.1590/1678-4685-GMB-2025-0083 (PMC12893196; doi:10.1590/1678-4685-GMB-2025-0083)
Supplement: Figure S2 - [file 1415-4757-GMB-49-s1-e20250083-s5.pdf]

Genetics and Molecular Biology
https://doi.org/10.1590/1678-4685-GMB-2025-0083

Supplementary Material to: Phyllosphere microbiomes uncovered: Research trends, geographic disparities, and key microbial players

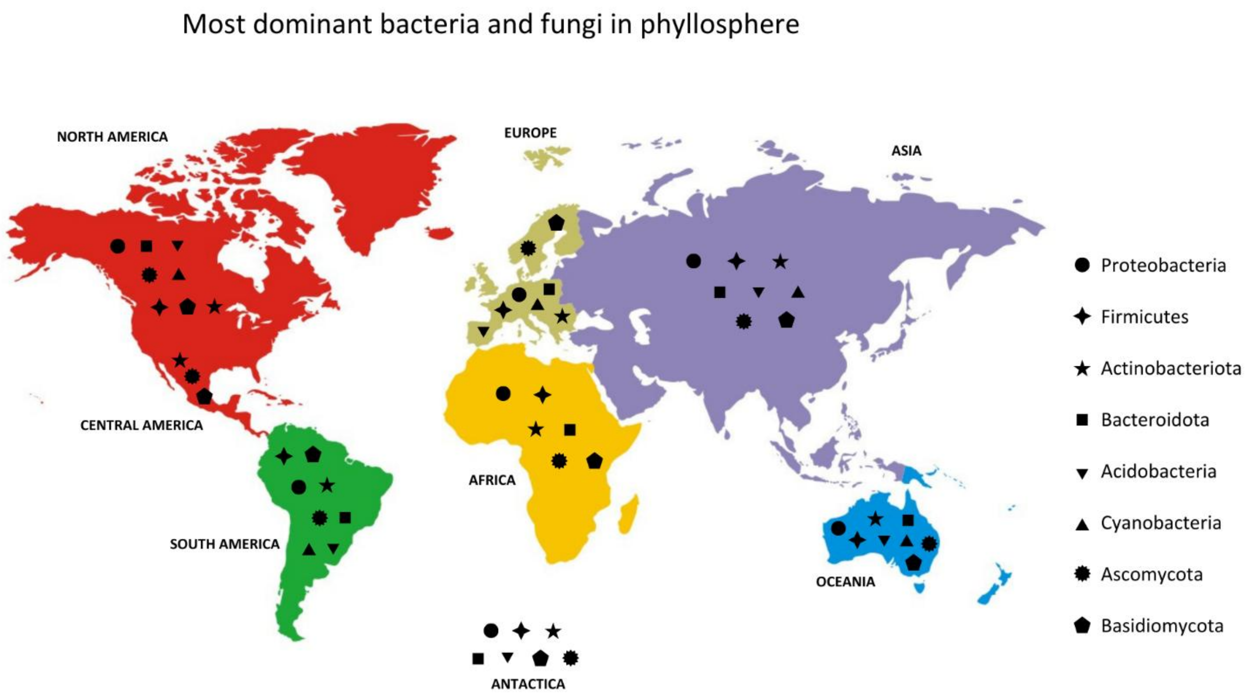

Map colors represent continents, with symbols indicating the most frequently reported dominant phyllosphere microbial groups in each region. Data are based on studies reporting predominant taxa from various plant species in different geographical regions.

Figure S2 -Global distribution of the most dominant bacterial and fungal taxa in the phyllosphere across continents.
